# Viral Infection Induces Expression of Novel Phased MicroRNAs from Conserved Cellular MicroRNA Precursors

**DOI:** 10.1371/journal.ppat.1002176

**Published:** 2011-08-25

**Authors:** Peng Du, Jianguo Wu, Jiayao Zhang, Shuqi Zhao, Hong Zheng, Ge Gao, Liping Wei, Yi Li

**Affiliations:** 1 Peking-Yale Joint Center for Plant Molecular Genetics and Agrobiotechnology, The State Key Laboratory of Protein and Plant Gene Research, College of Life Sciences, Peking University, Beijing, China; 2 Institute of Plant Virology, Fujian Agriculture and Forestry University, Fuzhou, Fujian, China; 3 School of Statistics, Renmin University of China, Beijing, China; 4 Center for Bioinformatics, The State Key Laboratory of Protein and Plant Gene Research, College of Life Sciences, Peking University, Beijing, China; University of California San Francisco, United States of America

## Abstract

RNA silencing, mediated by small RNAs including microRNAs (miRNAs) and small interfering RNAs (siRNAs), is a potent antiviral or antibacterial mechanism, besides regulating normal cellular gene expression critical for development and physiology. To gain insights into host small RNA metabolism under infections by different viruses, we used Solexa/Illumina deep sequencing to characterize the small RNA profiles of rice plants infected by two distinct viruses, *Rice dwarf virus* (RDV, dsRNA virus) and *Rice stripe virus* (RSV, a negative sense and ambisense RNA virus), respectively, as compared with those from non-infected plants. Our analyses showed that RSV infection enhanced the accumulation of some rice miRNA*s, but not their corresponding miRNAs, as well as accumulation of phased siRNAs from a particular precursor. Furthermore, RSV infection also induced the expression of novel miRNAs in a phased pattern from several conserved miRNA precursors. In comparison, no such changes in host small RNA expression was observed in RDV-infected rice plants. Significantly RSV infection elevated the expression levels of selective OsDCLs and OsAGOs, whereas RDV infection only affected the expression of certain OsRDRs. Our results provide a comparative analysis, via deep sequencing, of changes in the small RNA profiles and in the genes of RNA silencing machinery induced by different viruses in a natural and economically important crop host plant. They uncover new mechanisms and complexity of virus-host interactions that may have important implications for further studies on the evolution of cellular small RNA biogenesis that impact pathogen infection, pathogenesis, as well as organismal development.

## Author Summary

Small RNA-mediated RNA silencing is a widespread antiviral or antibacterial mechanism in different organisms. Although the host and pathogen factors involved in this mode of host defense and pathogen counter-defense have been extensively investigated, much less is known about how a pathogen alters the small RNA metabolism in a host. To help fill this knowledge gap, we first used deep sequencing to characterize the miRNA and siRNA profiles of rice plants infected by two distinct viruses, *Rice dwarf virus* (RDV) and *Rice stripe virus* (RSV), respectively. Our analyses showed that these two viruses had distinct impacts on rice small RNA metabolism. More significantly, RSV infection, but not RDV infection, enhanced the accumulation of some rice miRNA*s from conserved miRNA precursors and accumulation of phased siRNAs from a particular precursor. Furthermore, RSV infection also induced the expression of novel phased miRNAs from several conserved miRNA precursors. While RSV infection significantly elevated the expression of certain OsDCLs and OsAGOs, RDV infection only affected the expression of certain OsRDRs. These data uncover new mechanisms of virus-host interactions that affect host small RNA metabolism.

## Introduction

RNA-mediated gene silencing is a widespread mechanism of host defense against viral [1]–[6] and bacterial [7] infections. The 21–24 nt small RNAs, produced from DICER processing of double-stranded RNAs (dsRNAs) or RNA transcripts with stem-loop structures, are broadly defined as small interfering RNAs (siRNAs) and microRNAs (miRNAs), respectively [8]–[10]. They are incorporated into ARGONAUTES (AGOs) to form an RNA-INDUCED SILENCING COMPLEX (RISC). The RISC then recognizes its target RNA/DNA sequences through specific base pairing, to activate RNA cleavage or translation repression or DNA methylation [9], [11]–[14]. In plants, the miRNA precursors are processed into miRNA/miRNA* duplexes mostly by DICER-LIKE 1 (DCL1) with 2-nt 3′overhangs [9], [15], [16]. After methylation at the 3′ end, the miRNA sequences are preferentially incorporated into RISC to regulate gene expression, whereas the miRNA* sequences are usually degraded [17], [18].

Viruses encode dedicated proteins that function as viral suppressors of RNA silencing (VSRs), or other multi-functional proteins, to defend against host RNA silencing by interfering with distinct steps of the silencing pathways [19]. Previous studies, based on RNA gel blots, showed that transgenic expression of VSRs from many different plant viruses often caused reduced accumulation of conserved miRNAs [20]–[26]. The *Tobacco mosaic virus* movement and coat protein interactions also alter accumulation of tobacco miRNAs [27]. The biological function of this down-regulated miRNA accumulation for viral infection or plant defense remains to be understood.

The siRNAs are produced via processing of dsRNAs derived from distinct sources and are classified into three types: trans-acting siRNAs (ta-siRNAs), natural antisense transcript-derived siRNAs (nat-siRNAs) and repeat-associated siRNAs (ra-siRNAs). The ta-siRNAs are generated in a phased pattern through DCL4-processing of dsRNA substrates formed via the activity of RNA-DEPENDENT RNA POLYMERASE 6 (RDR6) [28]–[31]. The nat-sRNAs are produced from dsRNAs formed by natural antisense *cis*-transcript pairs [32], [33]. The ra-siRNAs are derived from transposons and repetitive elements [34], [35]. Transgenic expression of VSRs from some plant viruses also often led to reduced accumulation of siRNAs, likely as a means of dampening host silencing against viral infection [20], [23], [24].

There is evidence that some cellular miRNAs play anti-viral roles against animal viruses, although a particular miRNA is exploited to support viral infection [4], [5]. Many animal viruses cause generally down-regulated expression of host miRNAs as shown by microarrays [36]–[39] and quantitative real-time PCR [40]. Deep sequencing also revealed a similar pattern, and additionally identified a few new miRNAs induced only in virus-infected cells [41]–[43]. Some new miRNAs are induced in an organ-specific manner [41].

To gain further insights into viral interactions with the host RNA silencing pathways, we used deep sequencing to characterize the small RNA profiles of rice plants infected by two different RNA viruses together with microarray and quantitative RT-PCR analyses of the expression patterns of RNA silencing pathway genes. Rice is one of the most important crop plants and emerging models for RNA silencing studies [44]–[49]. *Rice dwarf virus* (RDV), which causes millions of dollars crop losses each year, is a member of *Phytoreovirus* whose genome consists of 12 dsRNAs that encode 12 proteins. The RDV non-structural protein Pns10 has been identified as a VSR, which has siRNA-duplex binding activities [50], [51]. *Rice stripe virus* (RSV), another devastating rice pathogen, is a member of *Tenuivirus* whose genome consists of four negative sense and ambisense single-stranded RNAs that encode seven proteins. The nonstructural protein NS3 functions as a VSR that also has siRNA-duplex binding activities [52]. Both viruses are transmitted via insect vector in a persistent manner and the eggs from viruliferous female adults also carry viruses and spread diseases. RSV and RDV are transmitted to rice plants by the small brown plant hopper (*Laodelphax striatellus*) and leafhopper (*Nephotettix cincticeps* or *Resilia dorsalis*), respectively.

Our analyses showed that RSV and RDV infections differentially affected rice small RNA profiles. RSV infection enhanced the accumulation of some rice miRNA*s, but not their corresponding miRNAs, as well as accumulation of phased siRNAs from a particular precursor. Significantly, RSV infection also induced the expression of novel miRNAs in a phased pattern from several conserved miRNA precursors. In comparison, no such changes in host small RNA expression was observed in RDV-infected rice plants. RSV infection significantly elevated the expression levels of selective OsDCLs and OsAGOs, whereas RDV infection only affected the expression of certain OsRDRs. Our results provide a comparative analysis, via deep sequencing, of changes in the small RNA profiles and in the genes of RNA silencing machinery induced by different viruses in a natural and economically important crop host plant. They uncover new mechanisms and complexity of virus-host interactions that may have important implications for further studies on the evolution of cellular small RNA biogenesis that impact pathogen infection, pathogenesis organismal development, and crop-protection technology development.

## Results

### Deep sequencing of small RNAs

We sequenced the small RNAs from rice plants infected with RDV and RSV, respectively, and from plants mock-inoculated controls by using the Solexa/Illumina deep sequencing method. The three-week-old rice seedlings were exposed, respectively, to viruliferous leafhopper, *N. cincticeps* (RDV), virus-free *N. cincticeps* (RDV, mock), viruliferous planthopper, *L. Striatellus* (RSV) and virus-free *L. Striatellus* (RSV, mock). After three weeks, when virus-induced symptoms appeared in the systemic leaves of virus-infected plants, the plants from each treatment (i.e., RDV-infected, RDV mock, RSV-infected and RSV mock) were pooled to prepare an RNA library for sequencing. We performed sequencing with three biological replicates for each treatment, with approximately 15 plants pooled in each replicate. (All the sequencing data can be available from the website: http://www.cbi.pku.edu.cn/download/rdsv/rdsv.html.) From each library, we obtained more than 50% of small RNA sequences that had at least one perfect match in the rice genome and no more than one mismatch in the virus genome (Table 1). The similar percentages of small RNA sequences matched to the rice and virus genomes in all replicates for a given treatment indicate similar quality of RNA preparation and sequencing. Because of the large volume of total sequence data and our primary focus in this study on learning about how the two viruses would affect small RNA profiles and the RNA silencing machinery in a common host, the virus-derived small RNA profiles and their biological implications will be analyzed and presented in a future report. For the rice small RNAs, we normalized the total sequence reads of each library to one million, and then used the average read value of unique sequences from all replicates in each treatment for further analysis.

**Table 1:** Summary of deep sequencing results of small RNAs from virus-infected and mock-inoculated rice small RNA libraries.

Libraries	Replicate 1				Replicate 2				Replicate 3			
	RDV^e^	Mock (RDV)^f^	RSV^g^	MocK (RSV)^h^	RDV^e^	Mock (RDV)^f^	RSV^g^	Mock (RSV)^h^	RDV^e^	Mock (RDV)^f^	RSV^g^	Mock (RSV)^h^
**Unique sequences** a	1332437	1465150	2407451	1677922	1950814	2063283	2949711	2146498	1631921	2098064	3136536	1803820
**Total sequences** b	3684420	3889709	6326816	5430579	5832235	5667068	5549056	5951022	4459388	5502449	5791793	5133329
**Unique sequences mapped to the rice genome** c	668381 (50.2)	831832 (56.8)	1496623 (62.1)	933907 (55.7)	1169075 (59.9)	1418070 (68.7)	2262510 (76.7)	1669607 (77.8)	955282 (58.5)	1664550 (79.3)	2412054 (76.9)	1427860 (79.2)
**Total sequences mapped to the rice genome**	2252948 (61.1%)	2792170 (71.8%)	4795509 (75.8%)	4057118 (74.4%)	2176965 (37.3%)	3179954 (56.1%)	3487733 (62.9%)	3137014 (52.7%)	1780344(39.9%)	2842475(51.7%)	3629722(62.6%)	2745570(53.4%)
**Unique sequences mapped to the virus genome** d	66510 (5.0%)	-	27146 (1.1%)	-	183087 (9.4%)	-	58678 (2.0%)	-	163225 (10.0%)	-	63344 (2.0%)	-
**Total sequences mapped to the virus genome**	408955 (11.1%)	-	79035 (1.2%)	-	1355885 (23.1%)	-	165244 (3.0%)	-	1094165(24.5%)	-	190237 (3.3%)	-

a Number of total sequences found within the set (18 nt< = Length< = 28nt).

b Total small RNA reads from each of the three replicates within the set.

c Sequences with perfect match to the rice genome, including those from tRNAs, rRNAs, snRNAs, or snoRNAs.

d Sequences with 1 mismatch to a virus genome.

e **RDV**: Small RNA library from RDV-infected rice.

f **Mock (RDV)**: Small RNA library from Mock (RDV)-inoculated rice.

g **RSV**: Small RNA library from RSV-infected rice.

h **Mock (RSV)**: Small RNA library from Mock (RSV)-inoculated rice.

### Infection by RSV, but not by RDV, led to altered expression of selective rice miRNAs

We compared the total reads of rice miRNAs among the four libraries, using pooled data from the three independent biological replicates. There were 4060, 4852, 8449 and 5014 unique sequences, with 89036, 91409, 118975 and 90214 reads, that match perfectly to miRNA precursors from RDV-infected, mock (RDV)-inoculated, RSV-infected, and mock (RSV)-inoculated rice plants, respectively (Table 2). Notably, the number of unique sequences mapped to miRNA precursors from the RSV-infected rice plants was nearly twice of those from each of the other three types of plants.

**Table 2:** Summary of small RNAs mapped to known rice miRNA precursors in virus-infected and mock-inoculated rice plants.

	RDV		Mock (RDV)		RSV		Mock (RSV)	
	Uniquesequences^c^	Reads/million^c^	Uniquesequences^c^	Reads/million^c^	Uniquesequences^c^	Reads/million^c^	Uniquesequences^c^	Reads/million^c^
**Precursor** a	4060	89036	4852	91409	8449	118975	5014	90214
**miRNA** b	818(20.1%)	84344(94.7%)	908(18.8%)	85112(93.1%)	1219(14.4%)	81975(68.9%)	929(18.5%)	82086(91.0%)
miRNA* b	131(3.2%)	1437(1.6%)	138(2.8%)	1813(2.0%)	287(3.4%)	32600^d^(27.4%)	143(2.9%)	1758(1.9%)
**Others**	3111(76.6%)	3255(3.7%)	3806(78.4%)	4484(4.9%)	6943(82.1%)	4400(3.7%)	3942(78.6%)	6370(7.1%)

a Perfect match to sense miRNA precursor sequences from the miRBase database (http://microrna.sanger.ac.uk/sequences, version 12.0).

b Encompasses the defined miRNA/miRNA* sequence ±2 nt on each side.

c Number of the unique and reads were the average values of each sample for the three replicates.

d The up-regulated small RNAs in RSV infected rice plants, but not in the other plants.

Of all the sequence reads mapped to miRNA precursors, 94.7% (84344), 93.1% (85112), 68.9% (81975) and 91.0% (82086) were mature miRNA sequences, and 1.6% (1437), 2.0% (1813), 27.4% (32600) and 1.9% (1758) were miRNA* sequences (Table 2). Interestingly, the reads of miRNA* sequences in the RSV-infected rice small RNA library were 15 times higher than those from the other three libraries, and was nearly half of the miRNA reads from the same library. The sequence data from the three biological replicates are presented in Supplemental Table S1, which reproducibly show more than about 10-times higher accumulation of miRNA* sequences in RSV-infected rice plants than in the other three groups of plants.

**Figure 1:**
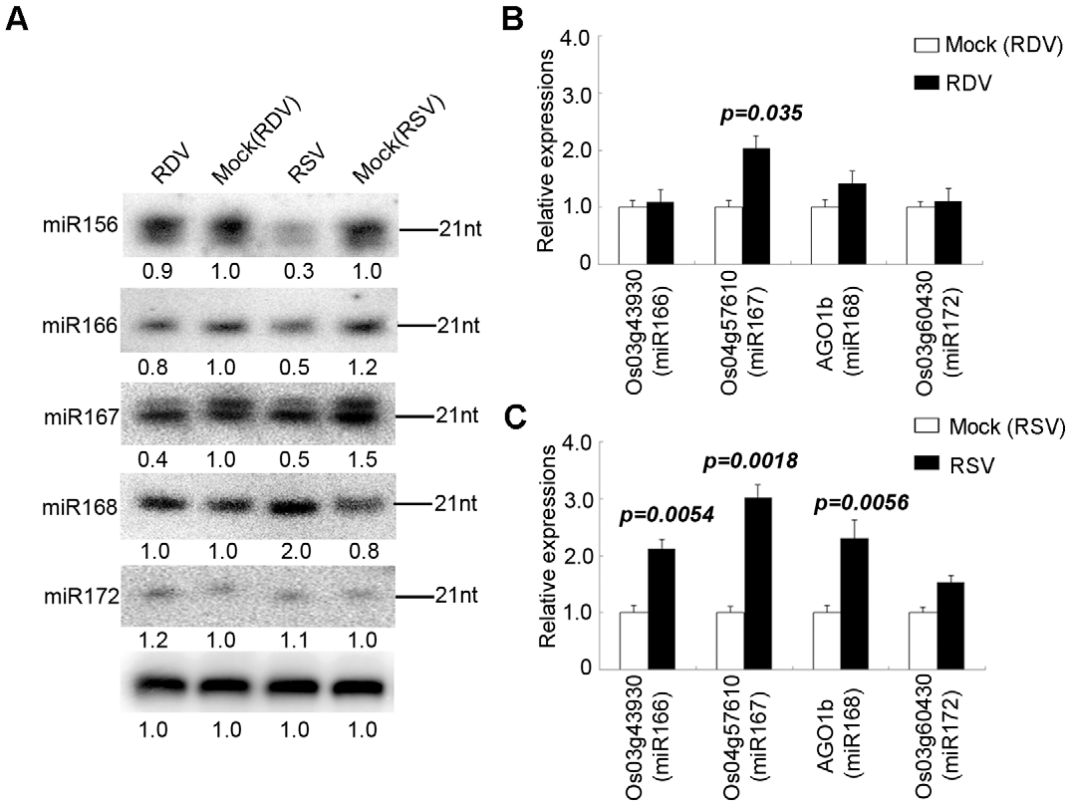
Confirmation of miRNA expression and analysis of miRNA target gene expression of in virus-infected plants. (**A**) RNA gel blots showing expression of miR156, miR164, miR166, miR167, miR168 and miR172 in virus infected rice plants. Rice U6 was reprobed as a control. The expression levels from mock (RDV)-inoculated plants are set to a value of 1.00 and the other three plants are expressed relative to this reference value. (**B**) Expression analysis of miRNA targets from RDV-infected plants and (**C**) from RSV-infected plants by quantitative real-time RT-PCR analysis. The expression levels of the assayed genes were normalized to the expression level of OsEF-1α.Os12g41680, Os03g43930, Os04g57610 and Os03g60430 are the targets of miR164, miR166, miR167 and miR172, respectively [54].

For many miRNAs there were no obvious differences in expression levels between RSV-infected and mock-inoculated rice plants. However, a number of miRNAs showed significant changes in expression in RSV-infected plants. These were defined as those having reads of 100 or more and showing at least two-fold changes in reads between RSV-infected and mock-inoculated plants. As shown in Table 3, expressions of miR156b, miR159a1, miR164a, miR166, miR167a, miR1884b, miR393b, miR396e and miR528 were down regulated, whereas miR535, miR390 and miR171 were up regulated in the infected plants. Although data from the three independent biological replicates were reproducible (Supplemental Table S1 and S2), we nonetheless used RNA gel blots to verify the altered expressions of some miRNAs with higher reads. As shown in Figure 1A, miR156, miR166 and miR167 were down regulated, whereas miR172 showed no obvious changes in accumulation. The expression of miR168 showed a two-fold increase on RNA gel blots, in near agreement with the sequence data (Table 3). By contrast, fewer miRNAs showed changes in accumulation levels in RDV-infected plant as compared to those in the mock-inoculated plants. Specifically, miR167a, miR171 and miR1863 were down regulated and only miR393 was induced in RDV-infected rice plants (Table 3). We confirmed the down-regulation of miR167 in RDV-infected rice in northern blots (Figure 1A). Thus, different viruses can differentially affect the expression of some miRNAs in a common host.

**Table 3:** Comparison of deep sequencing reads of known miRNAs and miRNA*s from virus-infected and mock-inoculated rice libraries.

miRNA family^a^	miRNA^b^					miRNA*^c^				
	Sequence (5′-3′)	RDV	Mock (RDV)	RSV	Mock (RSV)	Sequence (5′-3′)	RDV	Mock (RDV)	RSV	Mock (RSV)
miR1318	UCAGGAGAGAUGACACCGAC	25	27	30	21	CAGGUGUCAUCUCCCCUGAAC	2	1	50	1
miR1425	UAGGAUUCAAUCCUUGCUGCU	74	81	120	67	CAGCAAGAACUGGAUCUUAAU^e^	21	16	758	10
miR1432	AUCAGGAGAGAUGACACCGAC	14	12	38	9	AGGUGUCAUCUCCCCUGAACA	0	0	0	0
miR156b	UGACAGAAGAGAGUGAGCAC	8181	8445	1611	5510	GCUCACUCUCUAUCUGUCAGC	39	104	12	2
miR156c,g						GCUCACUUCUCUCUCUGUCAGC	40	99	119	93
miR156f						GCUCACUUCUCUUUCUGUCAGC	14	102	110	18
miR156h,j						GCUCGCUCCUCUUUCUGUCAGC	12	17	60	17
miR159a.1	UUUGGAUUGAAGGGAGCUCUG	7546	9633	1139	7857	GAGCUCCUUUCGGUCCAAAA	14	6	822	9
miR159a.2	UUGCAUGCCCCAGGAGCUGCA	5	3	3	4	AGCUGCUGGGUCAUGGAUC	7	3	181	8
miR160a,b	UGCCUGGCUCCCUGUAUGCCA	24	9	6	23	GCGUGCAAGGAGCCAAGCAUG	4	1	31	2
miR160c						GCGUGCACGGAGCCAAGCAUA^e^	1	1	240	1
miR160d						GCGUGCGAGGAGCCAAGCAUG	0	0	39	0
miR160e						GCGUGCGAGGUGCCAAGCAUG	0	0	12	0
miR160f	UGCCUGGCUCCCUGAAUGCCA	6	13	9	22	GCAUUGAGGGAGUCAUGCAGG	1	1	111	6
miR162a	UCGAUAAACCUCUGCAUCCAG	132	148	94	172	GGGCGCAGUGGUUUAUCGAUC	0	1	3	0
miR164a	UGGAGAAGCAGGGCACGUGCA	356	509	142	408	CACGUGGUCUCCUUCUCCAUC	0	0	1	0
miR164d	UGGAGAAGCAGGGCACGUGCU	14	33	12	12	CAUGUGCGCUCCUUCUCCAGC	0	0	2	0
miR164e	UGGAGAAGCAGGGCACGUGAG	12	6	5	16	CAUGUGUCCGUCUUCUCCACC	0	0	0	0
miR166a	UCGGACCAGGCUUCAUUCCCC	2031	1547	715	1566	GGAAUGUUGUCUGGUUCAAGG	12	8	336	7
miR166b						GGAAUGUUGUCUGGCUCGGGG^e^	5	2	103	3
miR166c						GGAAUGUUGUCUGGUCCGAG	0	0	22	0
miR166d						GGAAUGUUGUCUGGCUCGAGG^e^	6	5	99	7
miR166n						GAAUGACGUCCGGUCUGAAGA^e^	1	10	123	16
miR166e	TCGAACCAGGCTTCATTCCCC	2	1	1	2	GGAAUGUUGUCUGGUUCAAGG	12	8	336	7
miR166g	UCGGACCAGGCUUCAUUCCUC	263	224	101	160	AAUGGAGGCUGAUCCAAGAUC	2	1	140	2
miR166h						GGAAUGUUGGCUGGCUCGAGG	1	1	18	1
miR166i,j	UCUCGGAUCAGGCUUCAUUCC	28	45	19	15	AAUGCAGUUUGAUCCAAGAUC	0	3	20	1
miR166k	UCGGACCAGGCUUCAAUCCCU	184	174	124	126	GGUUUGUUGUCUGGCUCGAGG	1	0	26	0
miR166l							3	1	184	1
miR167a	UGAAGCUGCCAGCAUGAUCUA	369	771	189	786	GAUCAUGCAUGACAGCCUCAUU	0	0	15	2
miR167c						GGUCAUGCUGCGGCAGCCUCACU	0	0	20	0
miR167d	UGAAGCUGCCAGCAUGAUCUG	476	404	222	198	GAUCAUGCUGUGCAGUUUCAUC	3	6	106	14
miR167e,i						AGAUCAUGUUGCAGCUUCACU	4	3	131	10
miR167h						AGGUCAUGCUGUAGUUUCAUC	12	13	65	11
miR168a	UCGCUUGGUGCAGAUCGGGAC	38948	30643	51347	34152	CCCGCCUUGCACCAAGUGAAU	45	66	262	58
miR171c	UGAUUGAGCCGUGCCAAUAUC	49	144	243	78	GGAUAUUGGUGCGGUUCAAUC	0	0	16	1
miR171d						UGUUGGCCCGGCUCACUCAGA^e^	0	0	92	1
miR171e						UGUUGGCUCGGCUCACUCAGA^e^	2	1	510	1
miR171f						UGUUGGCAUGGUUCAAUCAAA^e^	4	24	187	22
miR171i	GGAUUGAGCCGCGUCAAUAUC	0	0	0	0	AGGUAUUGGCGUGCCUCAAUC	0	0	111	0
miR172d	AGAAUCUUGAUGAUGCUGCAU	502	611	337	349	GCAGCACCAUCAAGAUUCAC	1	1	14	0
miR1850.1	UGGAAAGUUGGGAGAUUGGGG	13	35	49	26	CCAAAUUCCCAACUUUUCAUC	0	0	1	0
miR1862d	ACUAGGUUUGUUUAUUUUGGGACG	43	65	23	43	UCCCAAAAUAAACAAAGCUAGUAC	0	0	2	0
miR1862e	CUAGAUUUGUUUAUUUUGGGACGG	41	56	60	41	AUCCCAAAAUGAAAAAAUCUAGUA	10	12	5	8
miR1863	AGCUCUGAUACCAUGUUAGAUUAG	149	465	135	247	AGUCUAAUAUGGUAUCCGAGCUUA	7	5	6	4
miR1867	UUUUUUUUCUAGGACAGAGGGAGU	75	77	42	86	UCCCUCUAUCCCAGAAAAAAACA	0	1	1	0
miR1868	UCACGGAAAACGAGGGAGCAGCCA	10	7	7	8	GCUACUUCCUCGUUUUCCGUAAAC	0	0	0	0
miR1870	UGCUGAAUUAGACCUAGUGGGCAU	30	43	99	56	GCCCUUUAGGGCUAAUUCAGCAUG				
miR1873	UCAACAUGGUAUCAGAGCUGGAAG	22	30	16	24	UCUAGCUCUGAUACCAUGUUGAGU	10	12	9	10
miR1878	ACUUAAUCUGGACACUAUAAAAGA	11	20	2	15	AUUUGUAGUGUUCAGAUUGAGUUU	36	65	12	57
miR1882e	AGAUUGCUUUCAAGGUCAUUUCUU	33	38	11	32	GAAAUGAUCUUGGACGUAAUCUAG	88	106	25	99
miR1883a	ACCUGUGACGGGCCGAGAAUGGAA	6	10	7	9	GGGUUCCAUUCUCGAUCCGUCACA	8	9	9	9
miR1884b	AAUGUAUGACGCUGUUGACUUUUA	70	124	43	152	AAAGUCAACGGUGUCAUAUAUUUA	11	21	18	9
miR390	AAGCUCAGGAGGGAUAGCGCC	24	30	149	20	CGCUAUCUAUCCUGAGCUCC	0	1	41	0
miR393b	UCCAAAGGGAUCGCAUUGAUC	171	65	24	483	UCAGUGCAAUCCCUUUGGAAU	569	834	212	969
miR396a,b	UUCCACAGCUUUCUUGAACUG	8	14	5	7	GUUCAAUAAAGCUGUGGGAA^e^	0	0	70	0
miR396c	UUCCACAGCUUUCUUGAACUU	406	722	313	970	GGUCAAGAAAGCUGUGGGAAG	22	7	1005	11
miR396e^e^	UCCACAGGCUUUCUUGAACUG	6465	8850	1459	8942	GUUCAAGAAAGCCCAUGGAA	15	11	2203	11
miR396f						GUUCAAGAAAGUCCUUGGAA^d^	245	117	22254	55
miR408	CUGCACUGCCUCUUCCCUGGC	3	6	7	22	CAGGGAUGAGGCAGAGCAUGG	5	10	50	25
miR444a.2	UGCAGUUGCUGCCUCAAGCUU	21	20	15	19	GCUAGAGGUGGCAACUGCAUA	5	6	11	6
miR444b.1	UGUUGUCUCAAGCUUGCUGCC	37	75	36	69	UGACAAGCUUGUGGCAGCAA	0	0	0	0
miR444c.1						CGGCAAGCUAGAGACAGCAAC	4	11	13	16
miR444b.2	UGCAGUUGUUGUCUCAAGCUU	241	270	191	153	GCUUGUGGCAGCAACUGCACA	51	77	335	54
miR528	UGGAAGGGGCAUGCAGAGGAG	241	376	204	493	CCUGUGCUUGCCUCUUCCAUU	0	0	16	3
miR535	UGACAACGAGAGAGAGCACGC	437	320	971	389	GUGCUUUCUCCCGUUGUCACU	7	5	38	10
miR810b.2	AAGUGAUUUAAUUAUGCCGUU	0	1	1	1	CGGCAUAAUUAGAUCACUUGAU	5	13	2	2

a Reads are the average values of each sample after being normalized to one million with the total sequence reads of each library.

b All miRNAs were reported in miRBase database, some of which were not sequenced in our libraries.

c The sequences are potential miRNA*s, some of which were not detected during our sequencing.

d The miR396f* is also reported as miR396f-3p(miRBase database: http://www.mirbase.org/).

e These sequences have 20-nt variants which have high reads in RSV-infected rice libraries.

Using quantitative real-time RT-PCR, we analyzed the expression of one target gene for each miRNA shown in Figure 1A. As shown in Figure 1B, the expression of AGO1b, a target of miR168, was induced during RSV infection (Figure 1C). This mimics the situation in *Arabidopsis thaliana* where the expressions of miR168 and AGO1 are transcriptionally co-regulated [53]. Os03g43930, a HD-Zip transcription factor and a target of miR166 [54], was up-regulated in agreement with the down-regulation of miR166 expression during RSV infection (Figure 1C). The expression of Os03g60430 (target of miR172) [55] showed no changes, whereas the expression of Os04g57610 (target of miR167) [54] increased in RSV infected rice (Figure 1C). These patterns correlated well with unaltered expression miR172 and down-regulated expression of miR167. Notably, none of these genes showed significant changes in expression levels, as did their cognitive miRNAs, in RDV-infected plants (Figure 1B). Using four different pairs of primers, we were unable to obtain conclusive data about the expression of potential miR156 targets (LOC_Os04g46580 and LOC_Os07g 32170).

### RSV infection enhanced accumulation of specific rice miRNA*

In RSV-infected rice plants, many miRNA*s accumulated to high levels, whereas their corresponding miRNA sequences did not show any obvious changes compared to the mock control (Table 3). These include miRNA*s for some members of the miR160 family (miR160a–f), miR166 family (miR166a–e, g–l and n), miR167 family (miR167a, c–e, h and i), miR171 family (miR171c–f and miR171i), miR396 family (miR396a–c, e and f) and miRNA* of miR1318, miR1425, miR159a, miR168, miR172d, miR390, miR444b.2 and miR528.

**Figure 2:**
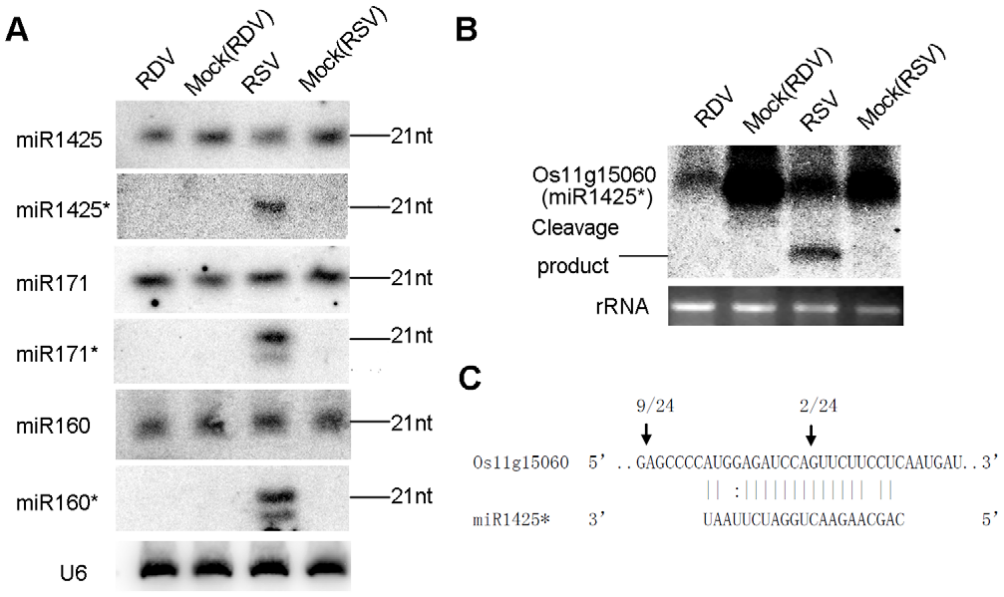
Predominant accumulation of selective miRNA*s in RSV-infected rice. (**A**) Expression analysis of miR1425/miR1425*, miR171/miR171* and miR160/miR160* in RDV infected, mock (RDV)-inoculated, RSV-infected as well as mock (RSV)-inoculated rice plants by RNA gel blots. Rice U6 was reprobed as a control. (**B**) RNA gel blot analysis of the expression of Os11g15060, a predicted target of miR1425*. The 28S rRNA stained with ethidium bromide was used as a loading control. (**C**) Mapping of the cleavage site in Os11g15060 by 5′-RACE. The numbers above the arrows indicate the frequencies of sequenced RACE clones corresponding to each inferred cleavage site.

These data were not attributed to sequencing bias. First, the vast majority of miRNA* in RSV-infected plants were present at low levels as in mock control plants. Second, the levels of both miRNA and miRNA* in RDV-infected plants were not different from those in the mock controls. Third, northern blots confirmed that miR1425*, miR160* and miR171* were significantly up regulated, whereas their corresponding miRNAs were not (Figure 2A). Thus, we concluded that RSV infection specifically enhanced the accumulation of certain miRNA* sequences, but not their corresponding miRNAs. We notice shorter sequences for miR160* and miR171* in RSV-infected rice (Figure 2A), which maybe 20-nt variants of miRNA* through sequencing data (Table 3; see also Table S2 for all sequencing data from three biological replicates).

We predicted the putative targets for some of these miRNAs*. Os11g15060, a SAM (S-adenosyl-L-Met)-dependent carboxyl methyltransferase, was predicted to be a target of miR1425*. Northern blots showed down-regulated expression of Os11g15060 in RSV-infected plants (Figure 2B). A cleavage product was detected (Figure 2B), and 5′-RACE (Rapid Amplification of 5′ Complementary DNA Ends) identified the cleavage site in the miR1425* binding region (Figure 2C), providing direct evidence that a plant miRNA* could direct the cleavage of its target mRNA. We also identified a second cleavage site outside the miR1425* binding region (Figure 2C). How this second site was derived is not clear, but many miRNA targets have more than one cleavage site as reported in other plants [56]–[58] and even in a green alga [59]. Whether this second cleavage results from the action of another yet-to-be identified small RNA induced by viral infection remains to be further investigated. Intriguingly, the expression of Os11g15060 was also down-regulated in RDV-infected plants (Figure 2B). However, absence of a cleavage product and failed 5′ RACE to identify a cleavage site (data not shown) suggests that this down-regulation in the RDV-infected plants was not caused by RNA silencing, or caused by partial silencing as well as another mechanism that remains to be identified. We also analyzed the expression of some potential targets of miR160* and miR171* including Os11g38140 and Os02g49240 (potential targets of miR160*), and Os03g38170 (potential target of miR171*). The expression of the three genes decreased in RSV-infected rice as compared with that in RDV-infected rice (Figure S1).

### RSV infection induced expression of new phased miRNAs from conserved precursors

**Figure 3:**
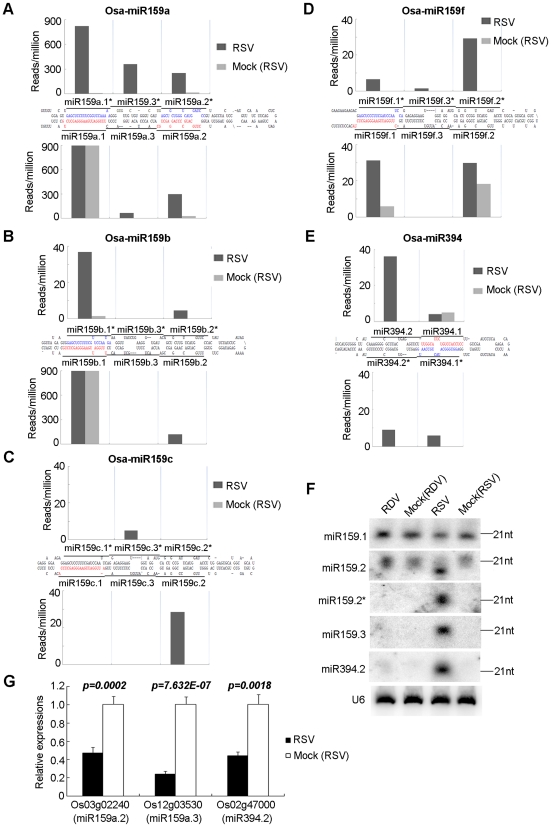
Phased miRNAs induced in RSV-infected rice. (**A**–**E**) Precursors of phased miRNAs. The sequences shown in red and blue are reported miRNAs and miRNA*s, respectively. The histograms indicate the reads of corresponding sequences on the precursors shown in black bars. (**F**) RNA gel blot showing the expression of phased miRNAs in RSV-infected rice. Rice U6 was reprobed as a control. (**G**) Expression analysis of the predicted targets of phased miRNAs by real time RT-PCR. Os03g02240 was previously validated as a target of miR159.2. [61]. Os12g03530 and Os02g4700 are predicted targets of miR159a.3 and miR394.2, respectively. The expression levels of the assayed genes were normalized to the expression level of OsEF-1α.

The small RNA libraries of RSV-infected rice contained many unique sequences, absent from the other three libraries, which are mapped to several conserved miRNA precursors (Table 2). Some of these belong to the miR159 family, whose precursors are approximately 200 nt in length with a stem structure of 80–90 nt (Figure 3). As shown in Figure 3A–D (see also Supplemental Table S3 for all sequence data), besides the reported miRNA/miRNA* pair for each precursor of the family (i.e., miR159a.1/miR159a.1*, miR159a.2/miR159a.2*, miR159b.1/miR159b.1*, miR159c.1/miR159c.1* and miR159f.1/miR159f.1*, labeled red and blue respectively for each pair), new pairs of miRNA/miRNA* were produced from each precursor. These included miR159a.3/miR159a.3*, miR159b.2/miR159b.2*, miR159b.3/miR159b.3*, miR159c.2/miR159c.2*, miR159c.3/miR159c.3*, miR159f.2/miR159f.2* and miR159f.3/miR159f.3*. These pairs were generated in a phased pattern and often detected at higher levels than the reported pairs. The phased miRNA-miRNA*s have the characteristics of 2 nt overhangs at the 3′ end. These observations, together with the observation that such phased production of new miRNA/miRNA* were absent from RDV-infected or any mock-infected plants, ruled out the possibility that they were degradation products or sequencing errors.

In addition to the above three-duplex phase forms of miRNA/miRNA*s, we also found two-duplex phase forms derived from some other precursors. The precursor of miR394 in the miRBase database (http://microrna.sanger.ac.uk/sequences, version 12.0) is about 100 nt in length. However, we found that the actual precursor is longer and contains a 27-nt extension from the 5′ and 3′ ends of the reported precursor, respectively (Figure 3E and Figure S2). In this longer precursor, a new miR394.2/394.2* duplex appeared at the distal end of the stem structure, in phase with the reported 394.1/394.1* (Figure 3E and Figure S2).

Northern blots confirmed the expression of miR159.2/miR159.2*, miR159.3 and miR394.2 (Figure 3F). The resolution of northern blots did not permit distinction between family members, so that each band could contain multiple members of a family of miRNAs/miRNA*s.

Using clustalW [60], we analyzed the conservation of precursor sequences of miR159, miR319, and miR394 in different plants (Figure S3). We found that, compared with the reported mature miRNA/miRNA* sequences, the newly identified phased miRNA/miRNA* sequences are less well conserved.

The target of miR159a.2 is Os03g02240, a GT2 transcription factor, which has been verified by 5′ RACE [61]. Quantitative real-time RT-PCR analysis showed that RSV infection down-regulated the expression of Os03g02240 (Figure 3G). Os12g03530 and Os02g47000, the predicted targets of miR159a.3 and miR394.2, respectively, were down-regulated in RSV-infected rice plants (Figure 3G). However, in RDV-infected rice plants, the expression of these genes was unchanged (data not shown).

### RSV infection enhanced the accumulation of distinct rice phased siRNA from a pre-existing RNA precursor

**Figure 4:**
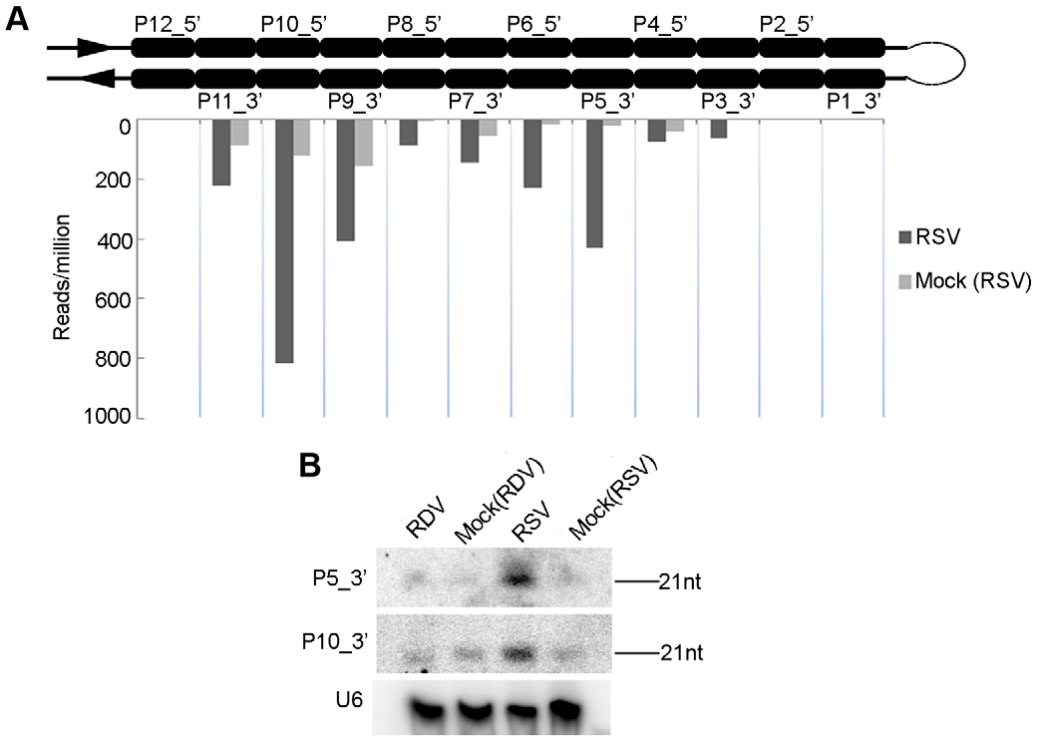
Induction of OsDCL4-dependent phased 21-nt siRNAs produced from the long hairpin of RNA encoded by the AK120922 locus in RSV-infected rice. (A) Accumulation of siRNAs in RSV-infected rice compared that in mock-inoculated rice. The upper structure illustrates the long RNA hairpin. The read numbers below the hairpin, which were each normalized to one million with total reads of the corresponding library, indicate the expression levels of siRNAs at corresponding positions in the 3′ arm of the hairpin. (B) RNA gel blots confirm accumulation of the highly induced P10_3′ and P5_3′, hybridized with the complementary ^32^P-labeled probes.

The transcripts encoded by the AK120922 locus of rice genome can fold into long inverted repeat structures, producing 12 21-nt phased small RNA duplexes [45], [62], [63]. From the stem region proximal to the terminal loop to the distal end of the RNA secondary structure, the 12 small RNA duplexes produced are named P1-12_5′ on the 5′ arm and P1-12_3′ on the 3′ arm (Figure 4A). One of these duplexes was initially characterized as miR436/miR436* duplex [62], but further studies demonstrated all small RNAs, including the so-called miR436, are DCL4-dependent siRNAs [45]. Analysis of our sequencing data showed that the reads of some AK120922-derived siRNAs in the RSV-infected rice plants were much higher than those in the mock-inoculated plants. In particular, the reads of P5_3′ increased by at least 100-fold. The higher expressions of these siRNAs were confirmed by northern blots (Figure 4B). No such changes were observed in RDV-infected plants (data not shown). Using realtime PCR, we analyzed the expression of the potential targets of P5_3′ and P10_3′. Surprisingly, we found that both of them were up-regulated in RSV-infected plants (Figure S4).

### RDV and RSV infections differentially modified the expression of rice RNA silencing pathway genes

To gain additional insights into the effects of RDV and RSV infection on the RNA silencing pathways/machinery, we characterized the expression profiles of various genes involved in the biogenesis/function of miRNAs/siRNAs by using the same plant materials as used for small RNA deep sequencing. These genes include 8 homologs of OsDCLs, five OsRDRs and 19 AGOs that have been identified in rice [44]–[49].

**Figure 5:**
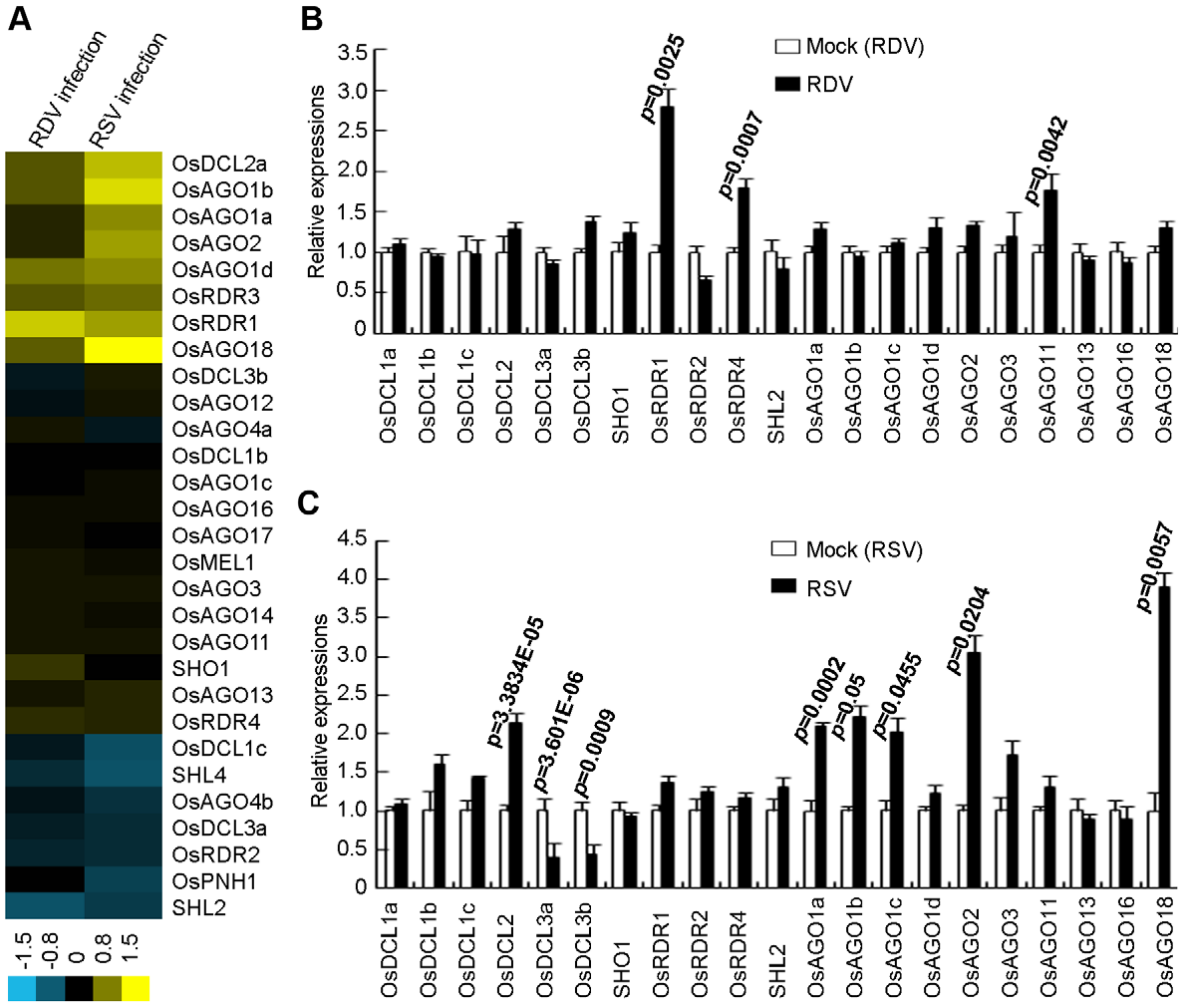
Expression analysis of OsDCLs, OsAGOs and OsRDRs of rice plants infected with RDV and RSV as compared to mock controls. (A) Expression profiles from microarrays. The bar on the bottom represents the scale of relative expression levels of genes (Log2). (B) Expression profiles from RDV-infected plants and (C) Expression profiles from RSV-infected plant based on quantitative real-time RT-PCR analysis. The expression levels of the assayed genes were normalized to the expression level of OsEF-1α. The expression level in the corresponding mock rice was set to 1.0. The nomenclature of these genes is based on Kapoor et al. (2008).

We first analyzed the expression profiles of OsDCLs, OsRDRs and OsAGOs, according to the annotations of Kapoor et al [48]. Figure 5A presents microarray data showing that RDV and RSV infections affected the expression of different members of OsDCL, OsRDR and OsAGO families. The microarray data were further verified by quantitative real-time RT-PCR measurements (Figure 5B and C). Of the 8 DCL homologs in the rice genome [48], OsDCL3a (LOC_Os01g68120) and OsDCL3b (LOC_Os10g34430) were significantly down-regulated, and OsDCL2 (LOC_Os03g38740) significantly up-regulated, in RSV-infected plants (Figure 5C). In contrast, RDV infection had almost no influence on the expression of OsDCLs in rice plants (Figure 5B).

In rice, there are four AGO1 homologs: OsAGO1a (LOC_Os02g45070), OsAGO1b (LOC_Os04g47870), OsAGO1c (LOC_Os02g58490) and OsAGO1d (LOC_Os06g51310) [32], [33]. The expression of OsAGO1a-c as well as OsAGO2 (LOC_Os04g52540) and OsAGO18 (LOC_Os07g28850) increased significantly in RSV-infected rice plants (Figure 5C). In RDV-infected plants, only the expression of OsAGO11 increased significantly (Figure 5B).

The expression of OsRDR1 (LOC_Os02g50330) and OsRDR4 (LOC_Os01g10140) was significantly enhanced in RDV-infected plants (Figure 5B), but not in RSV-infected plants (Figure 5C). OsRDR2 did not change expression levels in plants infected by either virus.

These data demonstrate that the two viruses have distinct effects on the expression of different genes of the RNA silencing pathways in the common host rice.

## Discussion

Recent studies used deep sequencing to obtain profiles of viral siRNAs [64]–[68] and viroid small RNAs [69], [70] in infected plants. Such data laid a foundation for further investigations on the biogenesis mechanisms and functions of viral and viroid small RNAs. Our present work provides the first deep sequencing analysis of plant small RNA profiles under viral infection conditions. This analysis not only filled a critical knowledge gap in RNA silencing-based virus-host interactions, but provided novel insights into the impact of viral infection on host small RNA biogenesis. Our results showed down-regulated as well as up-regulated accumulation of certain rice miRNAs, with up-regulation being more extensive. Most significantly, RSV infection, but not RDV infection, induced the expression of novel phased miRNAs from several families of conserved cellular miRNA precursors. Real-time RT-PCR experiments showed reduced accumulations of the predicted target mRNAs for these phased miRNAs (i.e., miR159.2 families, miR159.3 and miR394.2), indicating that the induced phased miRNAs have regulatory functions. How the reduced accumulation of these target mRNAs contributes to the establishment of viral infection and/or host defense will be an important focus of future research.

As compared to the short animal miRNA precursors, which are usually 70–80 nt, plant miRNA precursors are generally much longer with most known precursors to be 200–300 nt [15]. These long plant precursors make it possible to produce multiple miRNAs. Indeed, multiple, phased miRNAs are produced from some miRNA precursors in the single-cell alga, *Chlamydomonas reinhardtii* [59], [71] and in higher plants [72]. Some of these miRNAs are differentially regulated by bacterial infection in *A. thaliana* [72]. Our finding that new phased miRNAs are induced during the infection of a plant virus significantly broadens the landscape of phased miRNA biogenesis during pathogen infection. Further analyses of the host small RNA profiles involving different pathogens and hosts may lead to additional examples, and an understanding of the broad biological significance, of pathogen infection-induced expression of phased and other novel miRNAs.

The enhanced production of miRNA*s during RSV infection may be attributed to inhibition of RISC activity and miRNA/miRNA* unwinding by the RSV VSR, via direct or indirect interaction with the miRNA/miRNA* duplexes, as have been shown for VSRs of other viruses [22], [24]. If this were the case, one would expect elevated accumulations of miRNAs and miRNA*s at the same time. However, our deep sequencing showed that only the accumulations of miRNA*s, not the corresponding miRNAs from many miRNA precursors were enhanced. Such special accumulation of miRNA*, but not miRNAs, may be explained if RSV enhanced the activities of certain RISCs specially associated with miRNA*s or interfered with loading of some miRNAs into RISCs. Since we do not have data yet to support this postulation, we need to leave other alternative possibilities open for exploration. Schnettler et al. (2010) reported stabilization of miR171c/miR171c* complex by several tospoviruses in infected *N. benthamiana*, with elevated accumulation of miR171c* in the infected plants. This stabilization appears to be due to the activity of the viral NSs proteins [73].

The specific accumulation of miRNA*s of several miRNA families and the conservation of the miRNA* sequences within the families suggest that there may be certain base preferences among the miRNA*s accumulated during RSV infection. Using WebLogo [74], we found that there was an ‘A’ bias in the 19th nucleotide from the 5′ terminus (Figure S5). Considering that the *A. thaliana* AGO2 preferentially produces miRNA* sequences [75] and the up-regulation of OsAGO2 and OsAGO18 during RSV infection of rice, we propose that the ‘A’ bias may direct certain miRNA* sequences into these OsAGOs. Still we cannot exclude the potential influence of OsAGO1, as the ‘A’ bias in the 19th nucleotide from the 5′ terminus of miRNA* corresponds to the 5′ terminal ‘U’ of miRNA. In *Drosophila melanogaster*, miRNA*s were reported to have regulatory functions [76]. Here, by northern blots and 5′ RACE, we demonstrated that Os11g15060, a predicted target of miR1425*, was specifically down regulated by cleavage during RSV infection (Figure 2C). This is the first direct experimental demonstration, to the best of our knowledge, that a plant miRNA* can regulate the stability of its predicted target mRNA.

Although many siRNAs were produced in rice [77], our current analysis showed that RSV infection enhanced mainly the accumulation of some phased siRNAs derived from the AK120922 transcripts. The specificity of this enhancement was supported by the observation that RDV infection did not have such an effect. Previous studies showed that accumulation of siRNAs decreased in transgenic plants expressing the VSRs of several plant viruses [20], [23], [24]. The inhibitory effect of VSRs on siRNA accumulation, via binding of VSRs with siRNAs, is one of the mechanisms of viral counter-defense [1], [2], [19]. The enhanced accumulation of phased siRNAs from AK120922 during RSV infection, in contrast to the generally observed decrease in siRNA accumulations, may be biologically significant. Whether the VSR or other proteins encoded by RSV play a role in this enhancement is an outstanding mechanistic question to be answered in the future.

The induced expression of new phased miRNAs and the enhanced production of selective miRNA*s and phased siRNAs in rice plants infected by RSV, but not by RDV, indicate that such changes were not a general response to viral infection. Rather, they were caused by distinct virus-host interactions. One of the primary consequences of such interactions was conceivably altered expression and/or function of the RNA silencing machinery, which further leads to altered small RNA profiles. Indeed, our microarray and quantitative real-time RT-PCR analyses demonstrated that the expression files of OsDCLs, OsAGOs and OsRDRs were differentially altered in rice infected with the two viruses. During RDV infection, with the exception of OsRDR1 being enhanced, there were no significant changes in the expression of RNA silencing pathway genes. In contrast, during RSV infection, the expression levels of OsDCL3a and OsDCL3b were down-regulated, whereas the expression of OsDCL2 was enhanced. Three of the four OsAGO1 homologs, OsAGO1a, OsAGO1b and OsAGO1c, were up-regulated in RSV-infected plants. Whether the altered expression patterns of the OsDCLs, OsRDRs and OsAGOs are responsible for the altered miRNA/siRNA biogenesis/accumulations in the RSV- and RDV-infected rice plants remains to be determined. Current data indicate that OsDCL1 participates in the production of 21-nt miRNAs and DCL4 mainly produces some siRNAs [28], [29]. DCL3 was recently reported to produce 24-nt miRNAs [14]. The four small RNA libraries we generated comprised mainly 24-nt sequences. However, in the RSV-infected rice small RNA libraries, the number of 24-nt small RNAs was reduced to about three quarters of the other three libraries (Figure S6). This correlated with a decreased level of DCL3 expression in RSV-infected rice. These results suggested that OsDCL3 may be primarily responsible for producing 24 nt small RNAs, just as the *A. thaliana* homolog does (Figure 5) [78], [79]. The production of 21 nt and 24 nt phased miRNAs suggests involvement of DCL1 and potentially also DCL3.

In summary, our data indicate that at least some viruses may have evolved mechanisms to induce expression of phased miRNAs from well-conserved cellular miRNA precursors. Whether such new miRNAs play a role in host defense or in viral infection will be an important question to be investigated in the future. While this manuscript was under review, Hu et al. (2011) reported that *Oilseed rape mosaic tobamovirus* infection of *A. thaliana* also led to elevated accumulation of host siRNAs and some miRNA-like small RNAs (ml-sRNAs). These ml-sRNAs are derived in phase with known miRNAs from miRNA-precursors [80]. They are different from the sequences we report here. Altogether, as for many previous discoveries, virus-induced production of phased miRNAs and ml-sRNAs may provide yet another useful model system to study the molecular mechanisms underlying the evolution and biogenesis of miRNAs. It remains to be seen whether virus-induced biogenesis of phased miRNAs is more widespread in plants and other organisms. Another important question is how different viruses affect the production of different small RNAs and how such differences impact specific mechanisms of viral infection and host responses.

## Materials and Methods

### Plant growth, virus infection and small RNA sequencing

RDV Fujian isolate and RSV Jiangsu isolate, China, were maintained in “Oryza sativa spp. japonica” rice plants grown in greenhouses at 25±3°C, 55±5% RH and under natural sunlight. Insects (*Nephotettix cincticeps* and *Laodelphax striatellus*) were maintained in cages that contained rice seedlings in greenhouses at 25±3°C. To obtain high viruliferous insects, nymphs were reared on virus-infected rice plants for 1 week, and insects were maintained up to the adult stage with occasional replacement of seedlings by healthy rice seedlings. Rice seedlings were grown in a greenhouse at 25–28°C and 70±5% relative humidity under natural sunlight. Three-week-old seedlings were placed individually in single tubes of 4-cm in diameter and 25-cm in height that each contained 15–20 ml of liquid nutrient medium at the bottom. The viruliferous insects of *N. cincticeps* (carrying RDV), *L. Striatellus* (carrying RSV) as well as virus-free *N. cincticeps* (mock for RDV) and *L. Striatellus* (mock for RSV) were added to each tube. Each tube was then sealed with a nylon net. After 2 days in growth chambers with a 14-h/10-h light/dark cycle, 70±5% relative humidity and a temperature regime of 28°C (day)/25°C (night), the insects were removed and the rice seedlings were returned to the greenhouse to grow under the above greenhouse conditions. After approximately three weeks of growth, when the newly developed leaves started to show viral symptoms, the whole seedlings were harvested. With each sample, at least 15 rice seedlings were pooled for RNA extractions. Total RNAs were extracted using Trizol reagent (Invitrogen, Carlsbad, CA, USA) for RT-PCR, small RNA sequencing, microarray analysis, and northern blotting. RT-PCR was used to test individual rice seedlings for infection with RDV or RSV. All PCR primers are listed in Table S4. The small RNA library construction and Illumina 1G sequencing were carried out at BGI-Shenzhen (Shenzhen, Guangdong, China) using standard Solexa/Illumina protocols. Briefly, the total RNA was separated through 15% TBE urea denaturing PAGE gels and small RNAs of 15–30 nt were recovered. Then, 5′ and 3′ RNA adaptors were ligated to these small RNAs followed by reverse transcription into cDNAs. These cDNAs were finally amplified by PCR and subjected to Solexa/Illumina sequencing.

### Bioinformatics

After removing the adaptor sequences, small RNA sequences with 18–28 nt in length were used for further analysis. BOAT provided by CBI (http://boat.cbi.pku.edu.cn/) was used for mapping small RNAs to the *O. sativa* genome sequences (TIGR Rice Annotation Release 5.0, ftp://ftp.plantbiology.msu.edu/pub/data/Eukaryotic_Projects/o_sativa/annotation_dbs/) as well as RDV (ftp://ftp.ncbi.nih.gov/genomes/Viruses/Rice_dwarf_virus_uid14797/) and RSV (ftp://ftp.ncbi.nih.gov/genomes/Viruses/Rice_stripe_virus_uid14795/) Rice stripe virus) genome sequences. Small RNAs with perfect genomic matches were used for further analysis. The small RNAs were annotated with reference to the following databases: miRBase (http://microrna.sanger.ac.uk/sequences, version 12.0) for miRNA sequences, Rfam (http://www.sanger.ac.uk/Software/Rfam/) for noncoding RNA (rRNAs, tRNAs, snoRNAs, and snRNAs) sequences and Repbase (http://www.girinst.org) for transposons and repeats.

WebLogo [74] was used for analyzing of relative frequencies of nucleotides at each position of small the sRNAs, and the mfold program [81] was used for predicting the stem-loop structures. Target genes were predicted by using the miRU web server (http://bioinfo3.noble.org/miRNA/miRU.htm) and our own Perl script [59]. For submission to the web server, we chose the default parameters (score for each 20 nt≤3, G: U pairs≤6, indels≤1 and other mismatches ≤3) and the TIGR Rice Genome mRNA dataset (OSA1 release 5, 01/23/2007). For our own Perl script, based on the score standard mentioned before [59], targets with score ≤5 were chosen. Identical search results from both methods were considered potential targets of newly identified miRNAs or phased miRNAs. For miRNA*s induced during RSV infection, their targets were validated based on the microarray data. ClustalW was used for the alignment of miRNA precursors and miRNA* sequences.

### Microarray hybridization and data analysis

Hybridization of GeneChip rice genome array (Affymetrix), scanning and analysis were performed by the Affymetrix custom service (CapitalBio, Beijing, China) following standard protocols (http://www.affymetrix.com/support/technical/manual/expression_manual.affx). Three biological replicates were conducted for RNA from each type of plant samples. Data analysis and comparison of the samples was finished using the standard Affymetrix protocol (CapitalBio). Cluster3.0 software was used for cluster analysis. The expression profile of some mentioned gene through microarray analysis was shown in Table S5.

### Northern blot hybridization

Total RNA was used for mRNA and small RNA northern blot hybridization. For small RNA blots, 10–60 µg of total RNA (depending on the relative expression levels from deep sequencing) of each sample was separated on 15% polyacrylamide denaturing gels and then transferred to Hybond-N+ membranes (Amersham BioScience, Piscataway, NJ, USA). The membranes were cross-linked by UV transillumination and dried at 120°C for 30 min. DNA oligonucleotides complementary to small RNAs, which were labeled with γ-^32^P-ATP by T4 polynucleotide kinase (New England Biolabs, Beverly, MA, USA), were used as probes for hybridization. Membranes were prehybridized with buffer for 2 h followed by hybridization with the DNA probes overnight at 40°C in 5X SSC, 20 mM Na_2_HPO_4_ (PH 7.2), 7% SDS, 2X Denhardt's Solution, 100 µg/ml salmon sperm DNA. After washing twice at 40°C with 2X SSC and 0.1% SDS, the blots were imaged with a PhosphorImager (PerkinElmer, Shelton, CT, USA). The membranes were stripped with 0.1X SSC and 1% SDS at 100°C and reprobed. Probes complementary to U6 sequences were used as a loading control.

For mRNA blots, 15 µg of total RNA was separated by 1% formaldehyde agarose gel and transferred to Hybond-N+ membranes that were then cross-linked and dried as described above. Prehybridization and hybridization solution was 5X SSC, 1% SDS, 5X Denhardt's Solution, 100 µg/ml salmon sperm DNA and 50% formamide. The probes were labeled with α-^32^P-dCTP by DNA polymerase 1 large (Klenow) fragment (Promega, Madison, Wisconsin, USA) and were complementary to a 500-bp fragment corresponding to the 5′ partial sequence of Os11g15060 (target of miR1425*).

### Quantitative real-time RT-PCR

Total RNA was treated with RNase-free DNase I (TAKARA Biotechnology, Dalian, China) at 37°C for 30 min. After phenol/chloroform extraction and isopropanol precipitation, the RNA was quantified with a UV/ visible spectrophotometer (Amersham). Two µ\g of total RNA was reverse-transcribed using poly (T) adapter with SuperScript Reverse Transcriptase (Invitrogen). qPCR was performed using SYBR Green Realtime PCR Master Mix (Toyobo) OsEF-1a gene was used as an internal control, with primers CX1597 (59-GCACGCTCTTCTTGCTTTCACTCT-39) and CX1598 (59-AAAGGTCACCACCATACCAGGCTT-39) [45]. Three independent biological replicates were conducted. These data were further normalized with the normalized expression values obtained from qRT-PCRs and bar charts plotted by using Microsoft Excel and SPSS (Statistical Product and Service Solutions) software (IBM; Version.10.0). All the other primers used are listed in Supplemental Table S4.

### Target gene validation by 5′ RACE

Validation of target genes by mapping the cleavage sites was conducted with 5′ RACE by following the GeneRacer Kit (Invitrogen) protocols described previously [57]. Total RNA of RSV-infected rice was directly ligated to GeneRacer RNA Oligo adaptor without any modifications. RT-PCR was used to synthesize cDNAs using GeneRacer Oligo dT primer. GeneRacer 5′ Primer (5′CGACTGGAGCACGAGGACACTGA3′) and the target gene-specific outer primers (Table S4) were used for the first round of amplification. Then the GeneRacer 5′ Nested Primer (5′ GGACACTGACATGGACTGAAGGAGTA) and gene-specific inner primers (Table S4) were used for the second round of amplification. The PCR products were cloned and sequenced to identify the cleavage sites.

## Supporting Information

Figure S1 Realtime RT-PCR tested the expression of targets of miR160* and miR171* during RDV infection (**A**) and RSV infection (**B**). The expression levels of the assayed genes were normalized to the expression level of OsEF-1α. Os11g38140 and Os02g49240 were the potential targets of miR160*, and Os03g38170 was a potential target of miR171*.
(DOC)

Figure S2 Structure of miR394 precursor and small RNAs produced from miR394 precursor (A), The secondary structure of newly-identified miR394 precursor (165nt), which was done using Mfold. (B), Small RNAs generated from the new-identified miR394 precursors. The reported miRNA and miRNA* were shown in red and blue, and the new phased miRNA and miRNA* were shown in purple and indigo, respectively. The numbers following the small RNA sequences were the reads of the corresponding sequences in RSV-infected and mock (RSV) rice small RNA libraries of the first biological repeat.
(PDF)

Figure S3 Alignment of genomic sequences of phased miRNA precursors in different plants. The miRNA precursor sequences used for the alignment come from the miRBase database (http://microrna.sanger.ac.uk/sequences, version 12.0). Osa: Oryza sativa; sof: Saccharum officinarum; sbi: Sorghum bicolor; zma: Zea mays; ptc: Populus trichocarpa; ath: Arabidopsis thaliana; gma: Glycine max; vvi: Vitis vinifera; sly: Solanum lycopersicum; mtr: Medicago truncatula.
(PDF)

Figure S4 Realtime RT-PCR tested the expression of targets of P5_3′ and P10_3′ during RDV infection (**A**) and RSV infection (**B**). Os03g52630 was the potential targets of P5_3′, and Os04g51070 was a potential target of P10_3′. The expression levels of the assayed genes were normalized to the expression level of OsEF-1α.
(DOC)

Figure S5 The nucleotide bias of RSV induced miRNA* sequences calculated based on the unique sequences (A) and reads (B).
(DOC)

Figure S6 Size distribution of rice small RNAs in virus-infected and mock-inoculated rice. Proportion of unique sequences of different sizes in the total rice unique sequences of the four libraries.
(DOC)

Table S1 Summary of total small RNA reads mapped to known rice miRNA precursors in virus-infected and mock-inoculated rice plants. The footnotes of the table are as follows: ^a^ Reads were normalized to one million with the total reads of each library. ^b^ Perfect match to sense miRNA precursor sequences from the miRBase database (http://microrna.sanger.ac.uk/sequences, version 12.0). ^c^ Encompasses the defined miRNA/miRNA* sequence ±2 nt on each side. ^d^ Red indicates the up-regulated small RNAs in RSV infected rice plants, but not in the other plants.
(DOC)

Table S2 Sequencing reads of known miRNAs and miRNA*s in four libraries from three biological replicates. The footnotes of the table are as follows: ^a^ Reads were normalized to one million with the total sequence reads of each library. ^b^ The sequences are potential miRNA*s, some of which were not detected during our sequencing. ^c^ The miR396f* is also reported as miR396f-3p (miRBase database: http://www.mirbase.org/). ^d^ Red and green indicate miRNAs whose reads (> = 50 for miRNA in at least one sample) increased (red) or decreased (green) by at least two-fold in comparison with those from mock-inoculated plants in at least two biological replicates. They also indicate miRNA* sequences whose reads (> = 10 in at least one sample) increased (red) or decreased (green) by at least two-fold in virus-infected plants in comparison with those from mock-infected plants in at least two biological replicates. ^e^ These sequences have 20-nt variants which have high reads in RSV-infected rice libraries.
(DOC)

Table S3 Newly-identified miRNAs from the known miRNA precursors and their target genes. The footnotes of the table are as follows: ^a^ Reads was average values of three-repeat reads of each libraries, which normallized to 1 million with the total sequencing reads. ^b^ The precursor of miR394 is longer than the one in miRBase database, and miR394.2 have higher reads than miR394 only in RSV-infected rice library. ^c^ From the same position,other varient sequences with different length was produced. ^d^ The newly-identified miRNAs only have high reads in RSV-infected rice library, and form the phased-miRNA with the reported miRNA before . ^e^ The newly-identified miRNA are in tandem configuration with the reported miRNAs. ^f^ The sequences have much higher reads than the reported ones. ^g^ The miRNA sequences, reads number of which were outstanding shown in red and blue, were greatly accumulated and reduced only during RSV infection, respectively.
(DOC)

Table S4 Primers and probes used in this paper. * indicates the corresponding miRNA star sequences.
(DOC)

Table S5 Expressional analysis of RNA silencing pathway genes by microarray. The footnote of the table is as follows: ^a^ Fold change  =  (normalized signal intensity of virus-infected rice samples)/ (normalized signal intensity of mock-inoculated rice samples).
(DOC)
