# Supplementary figures and images for: Viral Infection Induces Expression of Novel Phased MicroRNAs from Conserved Cellular MicroRNA Precursors
Source: PLoS Pathog. 2011 Aug 25;7(8):e1002176. doi: 10.1371/journal.ppat.1002176 (PMC3161970; doi:10.1371/journal.ppat.1002176)

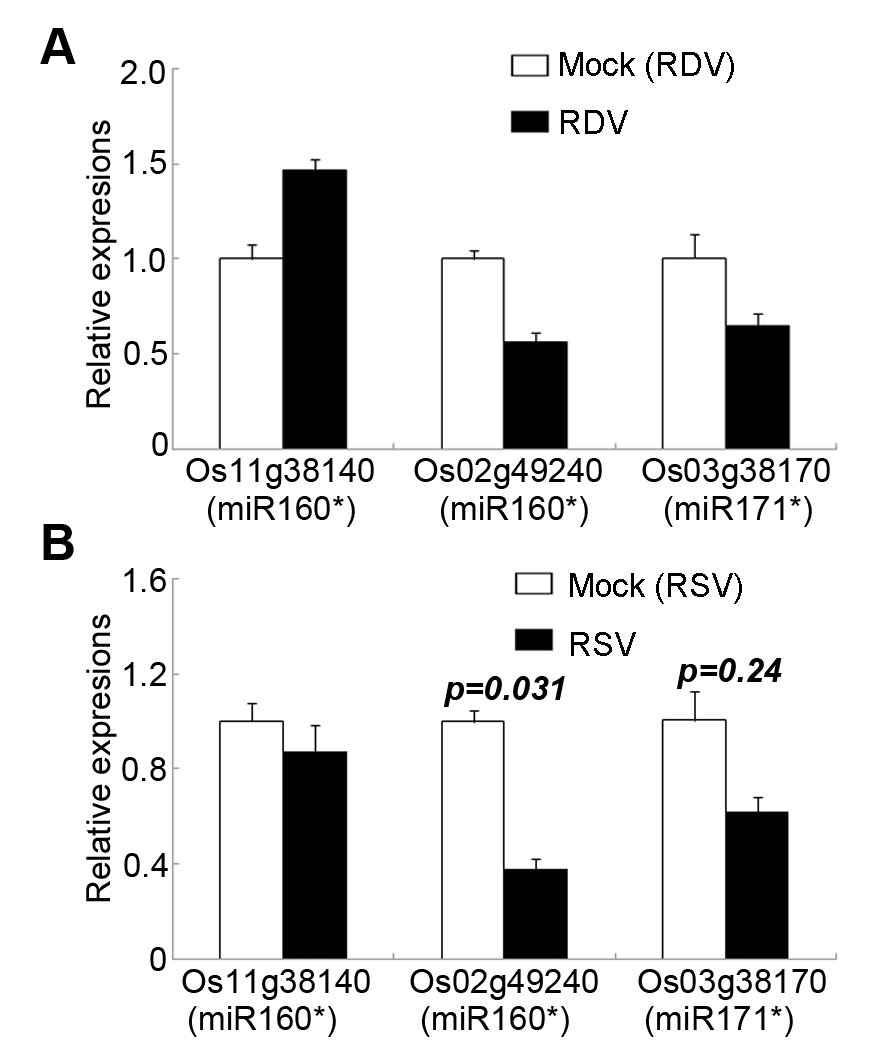

Supplement: Figure S1 — Realtime RT-PCR tested the expression of targets of miR160* and miR171* during RDV infection (A) and RSV infection (B). The expression levels of the assayed genes were normalized to the expression level of OsEF-1α. Os11g38140 and Os02g49240 were the potential targets of miR160*, and Os03g38170 was a potential target of miR171*. (DOC) [file ppat.1002176.s001.doc]

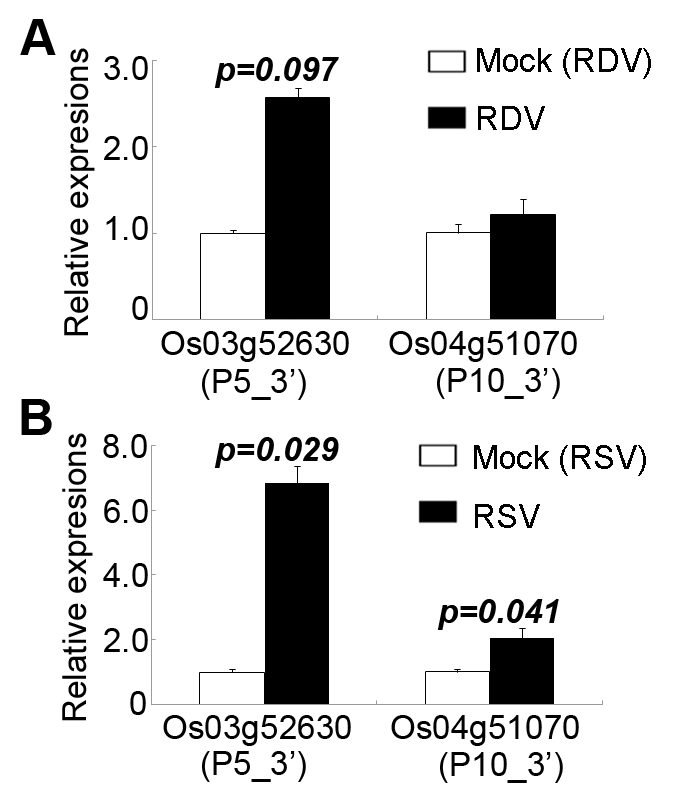

Supplement: Figure S4 — Realtime RT-PCR tested the expression of targets of P5_3′ and P10_3′ during RDV infection (A) and RSV infection (B). Os03g52630 was the potential targets of P5_3′, and Os04g51070 was a potential target of P10_3′. The expression levels of the assayed genes were normalized to the expression level of OsEF-1α. (DOC) [file ppat.1002176.s004.doc]

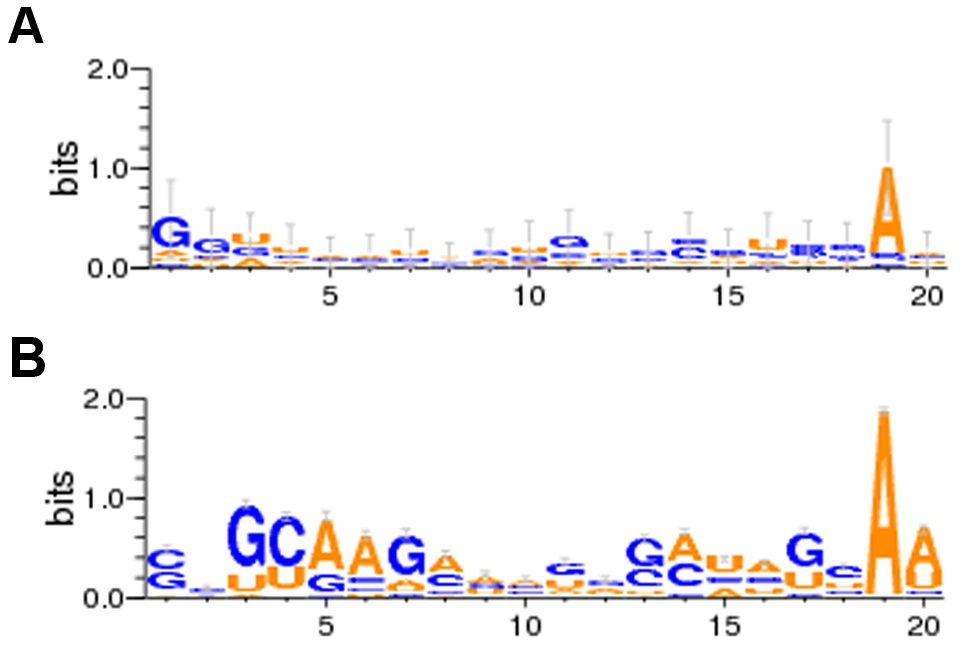

Supplement: Figure S5 — The nucleotide bias of RSV induced miRNA* sequences calculated based on the unique sequences (A) and reads (B). (DOC) [file ppat.1002176.s005.doc]

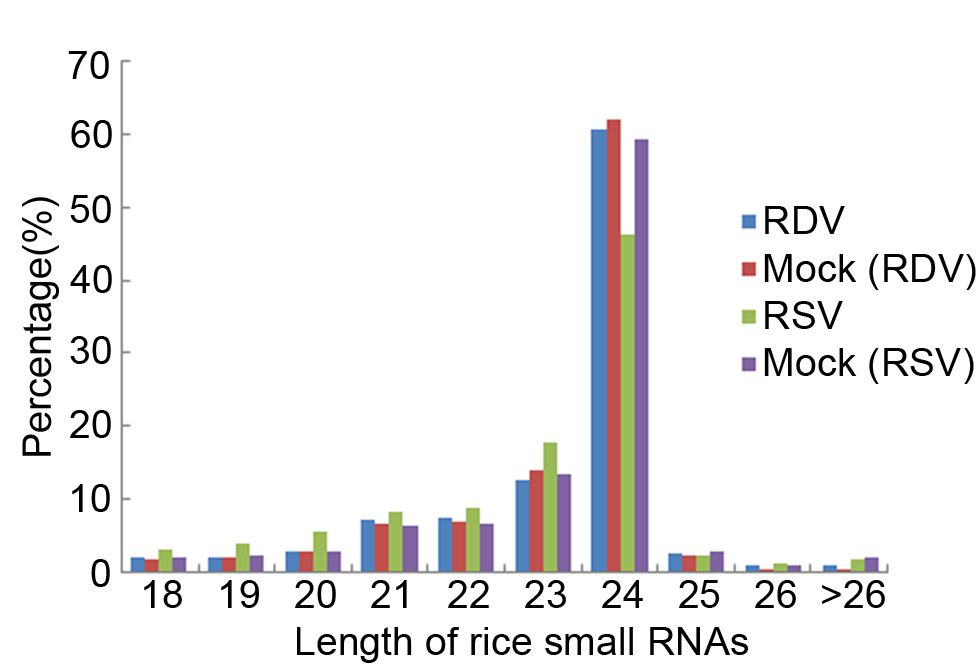

Supplement: Figure S6 — Size distribution of rice small RNAs in virus-infected and mock-inoculated rice. Proportion of unique sequences of different sizes in the total rice unique sequences of the four libraries. (DOC) [file ppat.1002176.s006.doc]
