# Supplementary material for: Viral Infection Induces Expression of Novel Phased MicroRNAs from Conserved Cellular MicroRNA Precursors
Source: PLoS Pathog. 2011 Aug 25;7(8):e1002176. doi: 10.1371/journal.ppat.1002176 (PMC3161970; doi:10.1371/journal.ppat.1002176)
Supplement: Figure S2 — Structure of miR394 precursor and small RNAs produced from miR394 precursor (A), The secondary structure of newly-identified miR394 precursor (165nt), which was done using Mfold. (B), Small RNAs generated from the new-identified miR394 precursors. The reported miRNA and miRNA* were shown in red and blue, and the new phased miRNA and miRNA* were shown in purple and indigo, respectively. The numbers following the small RNA sequences were the reads of the corresponding sequences in RSV-infected and mock (RSV) rice small RNA libraries of the first biological repeat. (PDF) [file ppat.1002176.s002.pdf]

A

```
10        20        30        40         50        60         70        80
|          CUUAU        C      UGAG      -      UUC           UU-     AUCCUCA    CA
GGUCAUGUGGG     CAAAGGGG GCUUAC    AGUUCU UUGGCA   UGUCCACCUCC   GUCGA       GAGA  G
UCAGUAUACCC     GUUUCCCU CGGAUG    UCGAGG AACCGU   ACGGGUGGAGG   UAGUU       CUCU  A
^          AAAAU        C      UG--      U      CAU           UUC     GUCUAUA    AA
  160       150       140         130       120       110       100        90
```

B

```
                                          UGGCAUUCUGUCCACCUCC 3,1
                                         UUGGCAUUCUGUCCACCUCC 27,28
                                         UUGGCAUUCUGUCCACCUC 3,1
                                        UUUGGCAUUCUGUCCACCUCC 3,2
                    GGGGCGCUUACUGAGAGU 1,0
                   AGGGGCGCUUACUGAGAGUUCU 1,0
                   AGGGGCGCUUACUGAGAGUUC 1,0
                   AGGGGCGCUUACUGAGAGUU 2,0
                   AGGGGCGCUUACUGAGAGU 4,0
                   AGGGGCGCUUACUGAGAG 1,0                                                                                       GCUGUGUAGGCCUCCCUUUG 1,0
                  AAGGGGCGCUUACUGAGAGUUCU 15,3                                                                                 AGCUGUGUAGGCCUCCCUUUG 5,0
                  AAGGGGCGCUUACUGAGAGUUC 28,0                                                                                  AGCUGUGUAGGCCUCCCUUU 9,0
                  AAGGGGCGCUUACUGAGAGUU 101,0                                                             AGGUGGGCAUACUGCCAAUGGA 1,0
                  AAGGGGCGCUUACUGAGAGU 290,0                                                              AGGUGGGCAUACUGCCAAUGG 7,0
                  AAGGGGCGCUUACUGAGAG 86,0                                                                AGGUGGGCAUACUGCCAAUG 25,0
                  AAGGGGCGCUUACUGAGA 4,0                                                                  AGGUGGGCAUACUGCCAAU 11,0
GGUCAUGUGGGCUUAUCAAAGGGGCGCUUACUGAGAGUUCUUUGGCAUUCUGUCCACCUCCUUGUCGAAUCCUCAGAGACAGAAAUCUCAUAUCUGUUGAUCUUGGAGGUGGGCAUACUGCCAAUGGAGCUGUGUAGGCCUCCCUUUGUAAAACCAUAUGACU
(((((((((((.....((((((((.((((((....((((((((((((...(((((((((((..(((((.......((((......)))).......)))))...)))))))))))...)))))).)))))).)))))).)))))))).....))))))))))) (-81.80)
```
