# Supplementary material for: Viral Infection Induces Expression of Novel Phased MicroRNAs from Conserved Cellular MicroRNA Precursors
Source: PLoS Pathog. 2011 Aug 25;7(8):e1002176. doi: 10.1371/journal.ppat.1002176 (PMC3161970; doi:10.1371/journal.ppat.1002176)
Supplement: Figure S3 — Alignment of genomic sequences of phased miRNA precursors in different plants. The miRNA precursor sequences used for the alignment come from the miRBase database (http://microrna.sanger.ac.uk/sequences, version 12.0). Osa: Oryza sativa; sof: Saccharum officinarum; sbi: Sorghum bicolor; zma: Zea mays; ptc: Populus trichocarpa; ath: Arabidopsis thaliana; gma: Glycine max; vvi: Vitis vinifera; sly: Solanum lycopersicum; mtr: Medicago truncatula. (PDF) [file ppat.1002176.s003.pdf]

3’ arms of precursors of miR159 family

```
                                            miR159.1              potential miR159.3             miR159.2
osa-miR159d         CCU--GCACUUGCAUGGGUUUGCAUGACCUGGGAGAUGAACCUGCC----------AUUGUGUUCCUCUAUU-GAUUGGAUUGAAGGGAGCUCC---GGCUACA-CCUA------
osa-miR159e         CCU--ACCCUUUCAUGGGUUUGCAUGACUCGGGAGAUGAACCCGCC----------AUUGUCUUCCUCUAUU-GAUUGGAUUGAAGGGAGCUCCUCUAGCUACA-U---------
osa-miR159c         CCU--GCACUUGCAUGGGUUUGCAUGACCCGGGAGAUGAACCCACC----------AUUGUCUUCCUCUAUU-GAUUGGAUUGAAGGGAGCUCCAC-AUCUCUC-UC--------
sof-miR159c         CCU--GCACUUGCAUGGGUGUGCAUGACCCGGGAGAUGAACCCACC----------AUCAUCUUUCCCUCGU-GCUUGGAUUGAAGGGAGCUCCUCUCUCUUUCCUCUCUCUCU-
sbi-miR159b         CCU--GCACUUGCAUGGGUGUGCAUGACCCGGGAGAUGAACCCACC----------AUCAUCUUUCCCUCGU-GCUUGGAUUGAAGGGAGCUCCUCUCUCUUUC-UCUCUUUCUC
zma-miR159d         CCU--GCACUUGCAUGGGUGUGCAUGACCCGGGAGACGAACCGACC----------AUCAUCUUUCCCUCGU-GCUUGGAUUGAAGGGAGCUCCUCUCUCUCUC-UCUCUCUUAU
zma-miR159c         CCUCUGCACUUGCAUGGGUGUGCAUGACCCGGGAGAUGAGCCCGCC----------AUCAUCUUUCCCUCGU-GCUUGGAUUGAAGGGAGCUCCUCUCUGUCUG-UCUGUCUGUC
ptc-miR159e         GCUGCGCAGGC-UAAGGGUCUGCAUGUGCUAGGAGAUGUGGU---U----------GCCUUGAUCUUUUUGG-UCUUGGGGUGAAGGGAGCUCCUAUA-GUCCCA----------
ptc-miR159f         GUUGCGCAGGC-UAAGGGUCUGCAUGACCUAGGAGACGUGGU---U----------ACCCUGACCCUUUUUG-UAUUGGAGUGAAGGGAGCUCG-AUG-GUCUUU----------
ptc-miR159d         GGGCUACAGCU-UAAGGGUUUGCAUGGCCAAGGAGACGUUGCC--U----------GCCUUCUCCUUUCGUU-UCUUGGAUUGAAGGGAGCUCCUACAUGUAUC-----------
osa-miR159f         ------CACUUGCAUGGGUUUGCAUGACCCGGGAGAUGAACCCACCAU-------UGUCUUCCUCUUAU----GCUUGGAUUGAAGGGAGCUCUACACCUCUCUC----------
ath-miR159c         AGC--ACACUUUAAGGGGAUUGCACGACCUCUUAGAUUCUCCCUCUUUCUCUACAUAUCAUUCUCUUCUCUUCGUUUGGAUUGAAGGGAGCUCCUUUUCUUCUUC----------
sof-miR159a         UUUGAGAUAGGCUUGUGGUUUGCAUGACCGAGGAGCUGCA--CCGUCCC----CUUGCUG------GCCGCU-CUUUGGAUUGAAGGGAGCUCUGCAUCCUGAUCCAUCCAUCC-
sof-miR159d         UAUGAGAUACGCUUGUGGUUUGCAUGACCGAUGAGCUGCA--CCGUCCC----CUUGCUG------GCCGCU-CUUUGGAUUGAAGGGAGCUCUGCAUCCUGAUCCAUCCAUCC-
sof-miR159b         UUUGAGAUAGGCUUGUGGUUUGCAUGACCGAGGAGCUGCA--CCGUCCC----CUUGCUG------GCCGCU-CUUUGGAUUGAAGGGAGCUCUGCAUCCUGAACCAUCCAUCC-
sof-miR159e         UUUGAGAUAGGCUUGUGGUUUGCAUGACCGAAGAGCUGCA--CCGUCCC----CUUGCUG------GCCGCU-CUUUGGAUUGAAAGGAGCUCUUGCAUCUGAUCCAUCCAUCC-
zma-miR159a         UUUGAGAUAGGCUUGUGGUUUGCAUGACCGAGGAGCUGCA--CCGCCCC----CUUGCUG------GCCGCU-CUUUGGAUUGAAGGGAGCUCUGCAUCCUGAUCCACCCCUCC-
osa-miR159b         ---GAGAUAGGCUUGUGGUUUGCAUGACCAAGGAGCCGAA--UCAACUC----CUUGCUG------ACCACU-CUUUGGAUUGAAGGGAGCUCUGCAUCUUGAUC----------
zma-miR159b         CUUGAGAUGGGCUUGUGGUUUGCAUGACCGAGGAGCUGCA--CGUCCCC----UUCGUUG------ACCGCU-GUUUGGAUUGAAGGGAGCUCUGCAUCUUGGUCCCAAGACUU-
ath-miR159a         -UUUGGUUAU----AUGGAUUGCAUAUCUCAGGAGCUUUAACUUGCCC-----UUUAAUGGCUUUUACUCUU-CUUUGGAUUGAAGGGAGCUCUAC------------------
ath-miR159b         -UAAGGUUUUUUUAUGGAUGCCAUAUCUCAGGAGCUUUCACUUACCCC----UUUAAUGGCUUC-ACUCUU-CUUUGGAUUGAAGGGAGCUCUUCAUCUCUC------------
ptc-miR159a         -UAGGUUUGC------GGCUUGCAUAUCUCAGGAGCUUUA--UUGCC--------UAAUG--UUAGAUCCCU-UUUUGGAUUGAAGGGAGCUCUAAACCCAUAA-----------
ptc-miR159c         -UAGGUUUGC------GGCUUGCAUAUCUCAGGAGCUUUA--UUGCC--------UAAUG--UUAGAUCCCU-UUUUGGAUUGAAGGGAGCUCUAAACCCAUAA-----------
ptc-miR159b         -UGGGCCUGC------GGCUUGCAUAUCUCAGGAGCUUUA--UUACC--------UAAUG--UUAGAUCUUU-UUUUGGAUUGAAGGGAGCUCUAAACCUUUGA-----------
gma-miR159a         -UAGUCUUGU------GGCUUCCAUAUUUGGGGAGCUUCA-UUUGCCUU----UAUAGUA----UUAACCUU-CUUUGGAUUGAAGGGAGCUCUACACCCUUCUCUUCUUUUCU-
osa-miR159a         GUUGGGAUAGGCUUAUGGCUUGCAUGCCCCAGGAGCUGCA--UCAACCC----UACAUGG-------ACCCU-CUUUGGAUUGAAGGGAGCUCUGCAUCUUUUGU----------
                                    **    **         **                                        ****  ****  *******
```

5’ arms of precursors of miR159 family

```
                     miR159.1*       potential miR159.3*     miR159.2*
osa-miR159d       GAGGAGCUCC-UUUCGAUCCAAUUCAGGAGAG--------GAAG---UGGUGGGAUGCAGCUGCCGGUUCAUGGAUACCUCUGCA-GUUCAU-GCCG
osa-miR159e       GAAGAGCUCCCUUUCGAUCCAAUUCAGGAGAG--------GAAG---UGGUAGGAUGCAGCUGCCGGUUCAUGGAUACCUCUGGA-GUGCAG-GGCA
osa-miR159c       GAGGAGCUCC-UUUCGAUCCAAUUCAGGAGAG--------GAAG---UGGUAGGAUGCAGCUGCCGAUUCAUGGAUACCUCUGGA-GUGCAU-GGCA
sof-miR159c       GAAGAGCUCC-CUUCGAUCCAAUCCAGGAGGG--------GAAG---UGGUCGGUUGCAGCUGCCGGUUCAUGGAUGCCUCUCUG-GUGCAGCAAUG
sbi-miR159b       AAAGAGCUCC-CUUCGAUCCAAUCCAGGAGGG--------GAAG---UGGUCGGUUGCAGCUGCCGGUUCAUGGAUGCCUCUCUG-GUGCAGCAAUG
zma-miR159d       GAAGAGCUCC-CUUCGAUCCAAUCCAGGAGGG--------AAAG---UGGUCGGCUGCAGCUGCCGGUUCAUGGAUACCUCUCUG-GUGCAGCAAUG
zma-miR159c       GAAGAGCUCC-CUUCGAUCCAAUCCAGGAGGG--------GAAG---UGGUCGGUUGCAGCUGCCGGUUCAUGGAUACCUCUCUG-GUGCAGCAAUG
ptc-miR159e       --AGGAGCUU-GCUCACUCCAGAUCUGAAAGG--------AGGU-GAUAGGAAACCACUUCUGCUACUUCAUGAAUACCCGUGGGUGUGCGCG--UA
ptc-miR159f       --GGAGCUCU-CCCCACUCCAUGCCUGAAAGG--------AGUUCGAUGGUAGACCAUGGCUGCUAGUUCAUGAAUACCCUUGGGUGCGCAGAAUUA
ptc-miR159d       UGGGAGCUUU-CUUUGGUUCAAAA-UGAGGAA--------AGAAAGUGGGUAUACU-CGUCUGCUUGUUCAUGGAUACCUCUGGGU-UGCGCAGGAU
osa-miR159f       GACGAGCUCC-CUUCGAUCCAAUCCAGGAGAG--------GAAGUGGUAGGAUGCAG---CUGCCGGUUCAUGGAUACCUCU---GCAGUGCAUGUC
ath-miR159c       AAGGAGCUCC-CUUCCUCCAAAACGAAGAGGACAAGAUUUGAGGAACUAAAAUGCAGAAUCUAAGAGUUCAUGUCUUCCUCAUAGAGAGUGCGCGGU
sof-miR159a       GCGGAGCUCC-UAUCAUUCCAAUGAAGGGCCG---------UUCUGAAGGG-UUGUUCCGCUGCUCGUUCAUGGUUCCCACUAUCCUAUCUCAUCAU
sof-miR159d       GCGGAGCUCC-UAUCAUUCCAAUGAAGGGCCG---------UUCUGAAGGG-UUGUUCCGCUGCUCGUUCAUGGUUCCCACUAUCCUAUCUCAUCAU
sof-miR159b       GCGGAGCUCC-UAUCAUUCCAAUGAAGGGCCG---------UUCUGAAGGG-UGGUUCCGCUGCUCGUUCAUGGUUCCCACUAUCCUAUCUCAUCAU
sof-miR159e       GCGGAGCUCC-UAUCAUUCCAAUGAAGGGCCG---------UUCUGAAGGG-UGGUUCCGCUGCUCGUUCAUGGUUCCCACUAUCCUAUCUCAUCAU
zma-miR159a       GCGGAGCUCC-UAUCAUUCCAAUGAAGGGUCG---------UUCCGAAGGGCUGGUUCCGCUGCUCGUUCAUGGUUCCCACUAUCCUAUCUCAUCAU
osa-miR159b       GUGGAGCUCC-UUUCGUUCCAAUGAAAGGUUU---------AUCUGAAGGG-UGAUACAGCUGCUUGUUCAUGGUUCCCACUAUUCUAUCUCAU---
zma-miR159b       GCGGUGCUCC-CUUCAAACCAAUAAACGGUCG---------AUCUGAUGGG-UGGUACAGCUGCUCGUUCAUGGUUCCCACUGUCCCAUCUCAUCA-
ath-miR159a       GUAGAGCUCC-UUAAAGUUCAAACAUGAGUUG---------AGCAGGGUAA--AGAAAAGCUGCUAAGCUAUGGAUCCCAUAAGCCCUAAUCCUUGU
ath-miR159b       GAAGAGCUCC-UUGAAGUUCAAUGGAGGGUUU---------AGCAGGGUGA--AGUAAAGCUGCUAAGCUAUGGAUCCCAUAAGCCUUAUCAAAUUC
ptc-miR159a       GUGGAGCUCC-UUGAAGUCCAAUAGAGGUUCU---------UGCUGGGUAG--A-UUAAGCUGCUAAGCUAUGGAUCC-ACA-GUCCUAUC--UAUC
ptc-miR159c       GUGGAGCUCC-UUGAAGUCCAAUAGAGGUUCU---------UGCUGGGUAG--A-UUAAGCUGCUAAGCUAUGGAUCC-ACA-GUCCUAUC--UAUC
ptc-miR159b       GUGGAGCUCC-UUGAAGUCCAAUAGAAGCUCC---------UGCUGGGUAG--A-UCGAGCUGCUGAGCUAUGAAUCCCACA-GCCCUAUCACCAUC
gma-miR159a       GUGGAGCUCC-UUGAAGUCCAAUUGAGGAUCU---------UACUGGGUGA--A-UUGAGCUGCUUAGCUAUGGAUCCCACAGUUCUACCCAUCAAU
osa-miR159a       GUUGAGCUCC-UUUCGGUCCAAAAAGGGGUGU---------UGCUGUGGGU-CGAUUGAGCUGCUGGGUCAUGGAUCCCGUUAGCCUACUCCAUGUU
                     *                *                                       **        ***  * *
```

3’ arms of precursors of miR319 family

```
                          miR319.2          potential miR319.3        miR319.1
ath-miR319b    ----GGUAAAUGAAUGAAUGAUGCGAGAG-ACAAAUUG--AGUCUUCACUUCUCUAUGCUUGGACUGAAGGGAGCUCCCU-----------------
aqc-miR319     ACUUGGUAAAUGAAUGAAUGAUCCGGGAG-AUUAAUUG--GAUCUUAUGCUUCUUGUACUUGGACUGAAGGGAGCUCCCUAC---------------
gma-miR319b    ACUCAGCAAAUGAGUGAAUGAUGCGGGAG-ACAAAUUG--AUUCUUAAGUUUCCUGUACUUGGACUGAAGGGAGCUCCCUUUUCUUUUGAAUCUUC
mtr-miR319b    ACUCAUCAAAUGAGUGAAUGAUGCGGGAG-ACAAAUUG--AAUCUUAAGUUUCCUAUACUUGGACUGAAGGGAGCUCCCUUUUC-------------
gma-miR319a    ACUCAGCAGAUGAGUGAAUGAUGCGGGAG-ACAAAUUG--AAUCUUAAGUUUCCUGUACUUGGACUGAAGGGAGCUCCCUUUUCCUUUUGUCUCUUA
sly-miR319     -CUCAGCAACUGAGUGAAUGAAGCGGGAGUACAAGUUG--AGUCUUGAGCUUCCUGUACUUGGACUGAAGGGAGCUCC-------------------
ptc-miR319a    ACCCAGUAAAUGAGUGAAUGAUGCGGGAG-ACAAAUUG--AAUCCUAAGCUUCCUGUACUUGGACUGAAGGGAGCUCCCUUUCCUUUU---------
ptc-miR319b    ACCCAGUAAAUGAAUGAGUGAUGCGGGAG-ACAAAUUA--AAUCUUAAACUUCCUAUCAUUGGACUGAAGGGAGCUCCCUUUACUGUU---------
vvi-miR319c    ACCCAGUAAAUGAUUGAAUGAUGCGGGAG-ACAAAUUG--GAUCUUAAGCUCCCUGUGCUUGGACUGAAGGGAGCUCCCUUCACUGCAAUC------
ptc-miR319c    CUAUUGUGAAUGUGUGAAUGAUGCGGGAG-AUAAAUUU-CAUCCUUUUCUUCUCUGUGCUUGGACUGAAGGGAGCUCCCUUUAAUCGU---------
ptc-miR319d    CUAUUGUGAAUGUGUGAAUGACGCGGGAG-AUUAAUUU-CAUCCUUUUCUUCUCUGUGCUUGGACUGAAGGGAGCUCCCUUUAAUUGU---------
vvi-miR319f    CUACUGUGAAUGUGUGAAUGAUGCGGGAG-GUAAAUUU-CAUCCUUUUCUUGUCUGUGCUUGGACUGAAGGGAGCUCCCUUCUACUGUUUU------
mtr-miR319     CUAUUGUGAAUGUGUGAAUGAUGCAGGAG-GUGAAUUU-CUUCCUUUUCUUCUUUG--CUUGGACUGAAGGGAGCUCCCUUUUUCUAUUUAUAAAUU
ptc-miR319f    CUACUGUGAUUGUGUGAAUGAUGCGGGAG-AUAAUUUUACAUCCCCUCUUUUUCUGUGCUUGGACUGAAGGGAGCUCCUUCCUUCUAU---------
ptc-miR319g    CUACUGUGAUUGUGUGAAUGAUGCGGGAG-AUAAUUUUCCAUCCUCUCCUUUUCUGUGCUUGGACUGAAGGGAGCUCCUUCCUUCUCU---------
ath-miR319c    CCGCAGUGACUGUGUGAAUGAUGCGGGAG-AUAUUUUU--GAUCCUUCUUUAUCUGUGUUUGGACUGAAGGGAGCUCCUUCUUUUUCUA--------
ath-miR319a    ACUCGUUAAAUGAAUGAAUGAUGCGGUAG-ACAAAUUG--GAUCAUUGAUUCUCUUUGAUUGGACUGAAGGGAGCUCCCUCU---------------
zma-miR319b    ---UUGCAGAUGAGUGAAUGAAGCGGGAGGUAAAAGCUUCGAUCUCGCACCGUCUUUGCUUGGACUGAAGGGUGCUCCCUCCUCCCU-CGCUCCUUG
zma-miR319d    ---UCGCAGAUGAGUGAAUGAAGCGGGAGGUAAAAGCUUCGAUCUCGCACCGUCUUUGCUUGGACUGAAGGGUGCUCCCUCCGAUC--CUUUCCUUG
sbi-miR319     GCUUUGCAGAUGAGUGAAUGAAGCGGGAGGUAAAAGCUUCGAUCUCGCACCAUCUUUGCUUGGACUGAAGGGUGCUCCCUCCUCCCCUCCUUAAUUG
osa-miR319b    GCUUAGCAGAUGAGUGAAUGAAGCGGGAGGUAAC-GUUCCGAUCUCGCGCCGUCUUUGCUUGGACUGAAGGGUGCUCCCUCCUCCUCGA--------
osa-miR319a    -CUUGGUAGCGGACUGGAUGACGCGGGAGCUAAAA--UUUAGCUCUGCGCCGUUUGUGGUUGGACUGAAGGGUGCUCCCUUGCUCAAGC--------
                          *   **  ***  *    **                     *     ************ *****
```

5’ arms of precursors of miR319 family

miR319.1* | potential miR319.3* | miR319.2*

```
ath-miR319b   ---------AGAGAGCUUUCUUCGGUCCACUCAUGGAGUAAUAU--GUGAGAUU-UAAUUGACUCUCGACUCAUUCAUCCAAAUACCAAAUGAAAGA
aqc-miR319    ---------AGAGAGCUCUCUUCAGUCCACUCAUAG-GUAGUA---GUCAGAUU-CAAUUUGCUGCCGACUCAUUCAUCCAUGUACCCAAGUUAGUA
gma-miR319b   GCGACGGUAAGAGAGCUUUCUUCAGUCCACUUAUGG-GUGACA---AUAAGAUUUCAAUUAGCUGCCGACUCAUUCAUCCAAAUGCUGAG-UGAAA-
mtr-miR319b   -------UAAGAGAGCUUUCUUUAGUCCACUCAUGG-GUGACA---AUAAGAUUUCAAUUAGCUGCUGACUCAUUCAUCCAAAUGUUGAG-UAAAAU
gma-miR319a   CCUAAGGUAAGAGAGCUUUCUUCAGUCCACUCAUGG-GUGACA---GUAAGAUU-CAAUUAGCUGCCGACUCAUUCAUCCAAAUGUUGAG-UGUAA-
sly-miR319    ------------GAGCUU-CUUUAGUCCACACAUGG-GGAACG---AUAGGGUU-CAAUUUGCUGCCGACUCAUUCAUCCAAAUGUUGAGGUCUUGA
ptc-miR319a   UAAUAGCUAAGAGAGCUUUCUUCAGUCCACUCAUGG-GUGGUA---GUAGGAUU-UAAUUAGCUGCCGACUCAUUCAUCCAAAUACUGAGUUAAAG-
ptc-miR319b   UAAUAGAUAAGAGAGCUUUUUUCGGUCCACUUAUAG-AUAGUA---AUAUGAUU-UAAUUAGUUACCGACUCAUUCAUCCAAAUACUGAGUUAUGA-
vvi-miR319c   UUACAUUGAAGAGAGCUUUCUUCAGUCCACUCAUGG-GUGGCA---GUAGGAUU-GAAUUAGCUGCCGACUCAUUCAUCCAAAUACUGUGUUAAG--
ptc-miR319c   AAUGGUUUAAGAGAGCUUUCUUCAGUCCACUCAUGGACGGG----CGAAGGGUUUGGAUUAGCUGCCGACUCAUUCAUUCAAACAC--AGUAGAAAU
ptc-miR319d   AAUGGUUUAACAGAGCUUCCUUCAGUCCACUCAUGGACGGG----CGAAGGGUUUGGAUUAGCUGCCGACUCAUUCAUUCAAACAC--AGUAGACAA
vvi-miR319f   AGUGGUUUAAGAGAGCUUCCUUCAGUCCACUCAUGGAUGGG----UUAGGGGUUUGGAUUAGCUGCCGACUCAUUCAUUCAAACAC--AGUAGAAUG
mtr-miR319    AAUAGAUGAAGAGAGCUUCCUUCAGUCCACUCAUGGAAGGG----UAAGGGGUUUGAAUUACCUGCUGACUCAUUGAUUCAAACAC--AAUAGACAA
ptc-miR319f   AAUGGUGGGAGAGAGCUUCCUUCAGCCCACUCAUGGAUAGGA---GAAAGGGGUUGAAUUAGCUGCCGACUCAUUCAUUCAAGCACC-AGUAGAAAA
ptc-miR319g   AAUCGUGGGAGAGAGCUUCUUUCAGCCCACUCGUGGAUAGGA---CAAAGGGGUUGAACUAGCUGCCGACUCAUUCAUUCAAGCACU-AGUAGAAAA
ath-miR319c   UAGAUAUAGAAGGAGAUUCUUUCAGUCCAGUCAUGGAUAGAAAAAGAAGAGGGUAGAAAUAUCUGCCGACUCAUCCAUCCAAACACU-CGUGGUAGA
ath-miR319a   -------AGAGAGAGCUUCCUUGAGUCCAUUCACAGGUCG-----UGAUAUGAUUCAAUUAGCUUCCGACUCAUUCAUCCAAAUACCGAGUCGCCAA
zma-miR319b   CUGGAUGGAAGAGAGCGUCCUUCAGUCCACUCAGGGGCGGU-----GCUAGGGUCGAAUUAGCUGCCGACUCAUUCACCCACAUGCCAAGCAAACGG
zma-miR319d   CCGGAUGGAAGAGAGCGUCCUUCAGUCCACUCAGGGGCGGU-----GCUAGGGUCGGAUUAGCUGCCGACUCAUUCACCCACAUGCCAAGCAAACGG
sbi-miR319    CCGGAUGGAAGAGAGCGUCCUUCAGUCCACUCAGGGGCGGU-----GCUAGGGUCGAAUUAGCUGCCGACUCAUUCACCCACAUGCCAAGCAAACGG
osa-miR319b   AUGGAUGGAAGAGAGCGUCCUUCAGUCCACUCAUGGGCGGU-----GCUAGGGUCGAAUUAGCUGCCGACUCAUUCACCCACAUGCCAAGCAAGAAA
osa-miR319a   UGUGUAAGAAGAGAGCUCUCUUCAGUCCACUCUCAGAUGGC-----UGUAGGGUUUUAUUAGCUGCCGAAUCAUCCAUUCACCUACCAAGAAAGUUG
                          ***     **  * ***      *                 *   * *   *  ** ****  *  **
```

5’ arms of precursors of miR394 family

```
                                 potential  miR394.2        miR394.1
zma-miR394a    ---------------------GGCGCUUACUGAAGAGUUCCUUGGCAUUCUGUCCACCUCC--UC
zma-miR394b    --------------------UGGCGCUUACUGA-GAGUUCCUUGGCAUUCUGUCCACCUCCCUUC
sbi-miR394a    --------------------CUUUACAUACUGA-GAGUUCCUUGGCAUUCUGUCCACCUCC----
osa-miR394     GUCAUGUGGGCUUAUCAAAGGGGCGCUUACUGA-GAGUUCUUUGGCAUUCUGUCCACCUCCUUGU
ptc-miR394a    -UCAUGUGGGUUUUGCAAAGGGUUUCUUACAGA---GUUUUUUGGCAUUCUGUCCACCUCC----
ptc-miR394b    -UCAUGUGGAUUUAGCAAAGGGUUUCUUACAGA---GUUUAUUGGCAUUCUGUCCACCUCCU---
ath-miR394a    -------------------------CUUACAGU---CAUCUUUGGCAUUCUGUCCACCUCCU---
ath-miR394b    -------------------------CUUACAGA---GAUCUUUGGCAUUCUGUCCACCUCCUCUC
                                        * *** *      *  ********************
```

3’ arms of precursors of miR394 family

```
                          miR394.1*         potential miR394.2*
zma-miR394a    UUUUUGGAGGUGGGCAUACUGCCAAUGGAGCUGUGUAGGCCUCC--------------------
zma-miR394b    UGUUUGGAGGUGGGCAUACUGCCAAUGGAGCUGCGUAGGCCUCC--------------------
sbi-miR394a    --UUUGGAGGUGGACAUACUGCCAAUGGAGCUGUGUAGGCCUCU--------------------
osa-miR394     --CUUGGAGGUGGGCAUACUGCCAAUGGAGCUGUGUAGGCCUCCCUUUGUAAAACCCAUAUGAC
ptc-miR394a    UUUCUGGAGGUGGGCAUACUGCCAACUGAGCUCUGUUGGUCUCUCUUUGUAAAACCCUCGUGA-
ptc-miR394b    CAUAUGGAGGUGGGCAUACUGCCAACCGAGCUCUGUUGGUCUCUCUUUGUAAAACCCUCGUGA-
ath-miR394a    AAGAAGGAGGUGGGUAUACUGCCAAUAGAGAUCUGUUAG-------------------------
ath-miR394b    UAAGAGGAGGUGGGCAUACUGCCAAUAGAGAUCUGUUAG-------------------------
                    ********  **********  *** *  **  *
```
