# Supplementary material for: Viral Infection Induces Expression of Novel Phased MicroRNAs from Conserved Cellular MicroRNA Precursors
Source: PLoS Pathog. 2011 Aug 25;7(8):e1002176. doi: 10.1371/journal.ppat.1002176 (PMC3161970; doi:10.1371/journal.ppat.1002176)
Supplement: Table S1 — Summary of total small RNA reads mapped to known rice miRNA precursors in virus-infected and mock-inoculated rice plants. The footnotes of the table are as follows: a Reads were normalized to one million with the total reads of each library. b Perfect match to sense miRNA precursor sequences from the miRBase database (http://microrna.sanger.ac.uk/sequences, version 12.0). c Encompasses the defined miRNA/miRNA* sequence ±2 nt on each side. d Red indicates the up-regulated small RNAs in RSV infected rice plants, but not in the other plants. (DOC) [file ppat.1002176.s007.doc]

| **Libraries^a^** | **Replicate 1** | | | |  | **Replicate 2** | | | |  | **Replicate 3** | | | |
| --- | --- | --- | --- | --- | --- | --- | --- | --- | --- | --- | --- | --- | --- | --- |
|  | **RDV** | **Mock**  **(RDV)** | **RSV** | **MocK**  **(RSV)** |  | **RDV** | **Mock**  **(RDV)** | **RSV** | **Mock**  **(RSV)** |  | **RDV** | **Mock**  **(RDV)** | **RSV** | **Mock**  **(RSV)** |
| **Precursors^b^** | 182510 | 156816 | 175019 | 132303 |  | 40472 | 75240 | 96379 | 63301 |  | 44126 | 42172 | 85529 | 75039 |
| **MiRNA^c^** | 179176  (98.1%) | 153010  (97.6%) | 168181  (96.1%) | 127321  (96.2%) |  | 34805  (86.0%) | 67547  (89.8%) | 40636  (42.2%) | 53277  (84.2%) |  | 39052  (88.5%) | 34779  (82.5%) | 37108  (43.4%) | 65658  (87.5%) |
| **MiRNA*^c^** | 471  (0.3%) | 531  (0.3%) | 4039^d^  (2.3%) | 349  (0.3%) |  | 1754  (4.3%) | 3471  (4.6%) | 53475^d^  (55.5%) | 2024  (3.2%) |  | 2086  (4.7%) | 1438  (3.4%) | 40288^d^  (47.1%) | 2901  (3.9%) |
| **Others** | 2863  (1.6%) | 3275  (2.1%) | 2799  (1.6%) | 4633  (3.5%) |  | 3913  (9.7%) | 4222  (5.6%) | 2268  (2.4%) | 8000  (12.6%) |  | 2988  (6.8%) | 5955  (14.1%) | 8133  (9.5%) | 6480  (8.6%) |
