# Supplementary material for: Viral Infection Induces Expression of Novel Phased MicroRNAs from Conserved Cellular MicroRNA Precursors
Source: PLoS Pathog. 2011 Aug 25;7(8):e1002176. doi: 10.1371/journal.ppat.1002176 (PMC3161970; doi:10.1371/journal.ppat.1002176)
Supplement: Table S2 — Sequencing reads of known miRNAs and miRNA*s in four libraries from three biological replicates. The footnotes of the table are as follows: a Reads were normalized to one million with the total sequence reads of each library. b The sequences are potential miRNA*s, some of which were not detected during our sequencing. c The miR396f* is also reported as miR396f-3p (miRBase database: http://www.mirbase.org/). d Red and green indicate miRNAs whose reads (> = 50 for miRNA in at least one sample) increased (red) or decreased (green) by at least two-fold in comparison with those from mock-inoculated plants in at least two biological replicates. They also indicate miRNA* sequences whose reads (> = 10 in at least one sample) increased (red) or decreased (green) by at least two-fold in virus-infected plants in comparison with those from mock-infected plants in at least two biological replicates. e These sequences have 20-nt variants which have high reads in RSV-infected rice libraries. (DOC) [file ppat.1002176.s008.doc]

|  | | | | | | | | | | | | | | |  |  |  |  |  |  |  |  |  |  |  |  |
| --- | --- | --- | --- | --- | --- | --- | --- | --- | --- | --- | --- | --- | --- | --- | --- | --- | --- | --- | --- | --- | --- | --- | --- | --- | --- | --- |
|  |  | **Replicate 1^a^** | |  |  | **Replicate 2^a^** | |  |  | **Replicate 3^a^** | |  |  |  | **Replicate 1^a^** | |  |  | **Replicate 2^a^** | |  |  | **Rpeat 3^a^** | |  |  |
| **Name** | **miRNA (5'-3')** | **RDV** | **Mock**  **(RDV)** | **RSV** | **Mock**  **(RSV)** | **RDV** | **Mock**  **(RDV)** | **RSV** | **Mock**  **(RSV)** | **RDV** | **Mock**  **(RDV)** | **RSV** | **Mock**  **(RSV)** | **miRNA* (5'-3')^b^** | **RDV** | **Mock**  **(RDV)** | **RSV** | **Mock**  **(RSV)** | **RDV** | **Mock**  **(RDV)** | **RSV** | **Mock**  **(RSV)** | **RDV** | **Mock**  **(RDV)** | **RSV** | **Mock**  **(RSV)** |
| miR1317 | TAGGGAACCCCATTCCCATAAA | 0 | 1 | 0 | 0 | 21 | 20 | 8 | 37 | 11 | 25 | 8 | 17 | CTATGGGAATAAGGGTTCCCTTA | 0 | 1 | 0 | 0 | 3 | 1 | 7 | 4 | 1 | 2 | 11 | 2 |
| miR1318 | TCAGGAGAGATGACACCGAC | 60 | 62 | 66 | 60 | 11 | 14 | 12 | 3 | 4 | 4 | 14 | 1 | CAGGTGTCATCTCCCCTGAAC | 1 | 0 | 3 | 0 | 5 | 2 | 85^d^ | 2 | 1 | 1 | 62 | 1 |
| miR1319 | AACCGGCATCTGTAATATATTATA | 0 | 0 | 0 | 0 | 0 | 0 | 0 | 0 | 0 | 0 | 0 | 0 | TAATATATTATAAGTGCCGGTTTT | 0 | 0 | 0 | 0 | 0 | 0 | 0 | 0 | 0 | 0 | 0 | 0 |
| miR1320 | TGGAACGGAGGAATTTTATAG | 7 | 4 | 3 | 1 | 5 | 28 | 21 | 3 | 9 | 1 | 27 | 2 | TGTAAAATTCATTCGTTCCAA | 1 | 0 | 1 | 1 | 26 | 70 | 16 | 15 | 25 | 5 | 29 | 16 |
| miR1423 | AGCGCCCAAGCGGTAGTTGTC | 0 | 0 | 0 | 0 | 0 | 0 | 1 | 0 | 0 | 0 | 0 | 0 | CAACTACACGTTGGGCGCTCGA | 0 | 0 | 0 | 0 | 0 | 0 | 0 | 0 | 0 | 0 | 0 | 0 |
| miR1424 | ATGCACACTGATGCTGATTGT | 0 | 0 | 0 | 0 | 0 | 0 | 0 | 0 | 0 | 0 | 0 | 0 | AGGCAGCTCAAAGTGTAGTC | 0 | 0 | 0 | 0 | 0 | 0 | 0 | 0 | 0 | 0 | 0 | 0 |
| miR1425 | TAGGATTCAATCCTTGCTGCT | 38 | 54 | 29 | 45 | 169 | 46 | 66 | 141 | 14 | 141 | 265 | 15 | CAGCAAGAACTGGATCTTAAT^e^ | 32 | 29 | 826 | 5 | 7 | 5 | 727 | 10 | 7 | 6 | 731 | 7 |
| miR1426 | AGAATCTTGATGATGATTAAA | 0 | 0 | 0 | 0 | 0 | 0 | 0 | 0 | 0 | 0 | 0 | 0 | TAATCATATTCGATTTTCAAA | 0 | 0 | 0 | 0 | 0 | 0 | 0 | 0 | 0 | 0 | 0 | 0 |
| miR1427 | TGCGGAACCGTGCGGTGGCGC | 0 | 0 | 0 | 0 | 0 | 0 | 0 | 0 | 0 | 0 | 0 | 0 | GCCACCGCGCGGTTCCGCAGCA | 0 | 0 | 0 | 0 | 0 | 0 | 0 | 0 | 0 | 0 | 0 | 0 |
| miR1428a-3p | TAAGATAAAGCCGTGAATTTG | 0 | 0 | 0 | 0 | 0 | 0 | 0 | 0 | 0 | 0 | 0 | 0 | TGCAAATTCGCAGGCCCTATCT | 0 | 0 | 0 | 0 | 0 | 0 | 0 | 0 | 0 | 0 | 0 | 0 |
| miR1428a-5p | CGTTTTGCAAATTCGCAGGCC | 0 | 0 | 0 | 0 | 0 | 0 | 0 | 0 | 0 | 0 | 1 | 0 | CCGTGAATTTGCAAAACGTT | 0 | 0 | 0 | 0 | 0 | 0 | 0 | 0 | 0 | 0 | 0 | 0 |
| miR1428b | TAAGATAATGCCATGAATTCG | 0 | 0 | 0 | 0 | 0 | 0 | 0 | 0 | 0 | 0 | 0 | 0 | AATTCACGGGTCTTATCTTGTG | 0 | 0 | 0 | 0 | 0 | 0 | 0 | 0 | 0 | 0 | 0 | 0 |
| miR1428c |  |  |  |  |  |  |  |  |  |  |  |  |  | AATTCGCAGGCCCTATCCTGTG | 0 | 0 | 0 | 0 | 0 | 0 | 0 | 0 | 0 | 0 | 0 | 0 |
| miR1428d |  |  |  |  |  |  |  |  |  |  |  |  |  | AATTCACAGGCCCTATATTGTG | 0 | 0 | 0 | 0 | 0 | 0 | 0 | 0 | 0 | 0 | 0 | 0 |
| miR1428e-3p | TAAGATAATGCCATGAATTTG | 0 | 1 | 1 | 1 | 14 | 13 | 15 | 10 | 9 | 3 | 16 | 5 | - | 0 | 0 | 0 | 0 | 0 | 0 | 0 | 0 | 0 | 0 | 0 | 0 |
| miR1428e-5p | AATTCACAGGCCCTATCTTGTG | 0 | 0 | 0 | 0 | 1 | 3 | 1 | 2 | 2 | 1 | 1 | 1 | - |  |  |  |  |  |  |  |  |  |  |  |  |
| miR1428f-5p |  |  |  |  |  |  |  |  |  |  |  |  |  | TAAGATAATGCCGTGAATTCG | 0 | 0 | 0 | 0 | 0 | 0 | 0 | 0 | 0 | 0 | 0 | 0 |
| miR1428g-5p |  |  |  |  |  |  |  |  |  |  |  |  |  | AAAGATAATGCTGTGAATTTG | 0 | 0 | 0 | 0 | 0 | 0 | 0 | 0 | 0 | 0 | 0 | 0 |
| miR1429-3p | GTTGCACGGGTTTGTATGTTG | 0 | 0 | 0 | 0 | 0 | 0 | 0 | 0 | 0 | 0 | 0 | 0 | - |  |  |  |  |  |  |  |  |  |  |  |  |
| miR1429-5p | GTAATATACTAATCCGTGCAT | 0 | 0 | 0 | 0 | 0 | 0 | 0 | 0 | 0 | 0 | 1 | 0 | - |  |  |  |  |  |  |  |  |  |  |  |  |
| miR1430 | TGGTGAGCCTTCCTGGCTAAG | 0 | 0 | 0 | 0 | 0 | 0 | 0 | 0 | 0 | 0 | 0 | 0 | TAGCCAAGAATGGCTTGCCTATC | 0 | 0 | 0 | 1 | 1 | 1 | 3 | 5 | 1 | 5 | 1 | 0 |
| miR1431 | TTTGCGAGTTGGCCCGCTTGC | 1 | 0 | 0 | 0 | 2 | 0 | 4 | 4 | 3 | 1 | 4 | 7 | AAGCGGGTCAACCCGTGAACC | 0 | 0 | 0 | 0 | 0 | 0 | 1 | 0 | 0 | 0 | 1 | 0 |
| miR1432 | ATCAGGAGAGATGACACCGAC | 26 | 20 | 60 | 19 | 11 | 14 | 25 | 5 | 6 | 2 | 30 | 2 | AGGTGTCATCTCCCCTGAACA | 0 | 0 | 0 | 0 | 0 | 0 | 0 | 0 | 0 | 0 | 0 | 0 |
| miR1433 | TGGCAAGTCTCCTCGGCTACC | 0 | 0 | 0 | 0 | 0 | 0 | 2 | 1 | 0 | 4 | 1 | 0 | TAGCCAAGGATGATTTGCCTGT | 0 | 0 | 0 | 0 | 6 | 2 | 7 | 26 | 1 | 37 | 18 | 4 |
| miR1435 | TTTCTTAAGTCAAACTTTTT | 0 | 0 | 0 | 0 | 0 | 0 | 0 | 0 | 0 | 0 | 0 | 0 | GTAGTTTGACTTAAAAAAAG | 0 | 0 | 0 | 0 | 0 | 0 | 0 | 0 | 0 | 0 | 0 | 0 |
| miR1436 | ACATTATGGGACGGAGGGAGT | 0 | 0 | 0 | 0 | 0 | 0 | 0 | 0 | 0 | 0 | 0 | 0 | TCCCTCCGTCCCATAATATTA | 0 | 0 | 0 | 0 | 0 | 0 | 0 | 0 | 0 | 0 | 0 | 0 |
| miR1437 | TCCGGCGCCGCACTAGGCACTG | 0 | 0 | 0 | 0 | 0 | 0 | 0 | 0 | 0 | 0 | 0 | 0 | GTGCCTAGTGCGGCACCGGAG | 0 | 0 | 0 | 0 | 0 | 0 | 0 | 0 | 0 | 0 | 0 | 0 |
| miR1438 | AGGGTAATTTTATCATTTTTA | 0 | 0 | 0 | 0 | 0 | 0 | 0 | 0 | 0 | 0 | 0 | 0 | AAAAATTATAAAATTACCCTAA | 0 | 0 | 0 | 0 | 0 | 0 | 0 | 0 | 0 | 0 | 0 | 0 |
| miR1439 | TTTTGGAACGGAGTGAGTATT | 0 | 0 | 0 | 0 | 0 | 0 | 0 | 0 | 0 | 0 | 0 | 0 | TACTACCTCCGTCCCAAAATA | 0 | 0 | 0 | 0 | 0 | 0 | 0 | 0 | 0 | 0 | 0 | 0 |
| miR1440 | TGCTCAAATACCACTCTCCT | 0 | 0 | 0 | 0 | 0 | 0 | 0 | 0 | 0 | 0 | 0 | 0 | GAGAGTGGTATTTGAGCACT | 0 | 0 | 0 | 0 | 0 | 0 | 0 | 0 | 0 | 0 | 0 | 0 |
| miR1441 | ACCGGATGTCGGAAAAGGTTT | 0 | 0 | 0 | 0 | 0 | 0 | 0 | 0 | 0 | 0 | 0 | 0 | ACTCCTTTTGATATCTGGTCA | 0 | 0 | 0 | 0 | 0 | 0 | 0 | 0 | 0 | 0 | 0 | 0 |
| miR1442 | ATTCATAGTACTAGATGTGT | 0 | 0 | 0 | 0 | 0 | 0 | 0 | 0 | 0 | 0 | 0 | 0 | ACATCCTAGTAAAATGAATCT | 0 | 0 | 0 | 0 | 0 | 0 | 0 | 0 | 0 | 0 | 0 | 0 |
| miR156a | TGACAGAAGAGAGTGAGCAC | 24448 | 23500 | 3667 | 15612 | 225 | 559 | 686 | 768 | 132 | 1274 | 526 | 349 | GCTCACTTCTCTCTCTGTCACC | 0 | 0 | 0 | 0 | 0 | 1 | 1 | 0 | 0 | 1 | 0 | 1 |
| miR156b |  |  |  |  |  |  |  |  |  |  |  |  |  | GCTCACTCTCTATCTGTCAGC | 5 | 4 | 4 | 3 | 53 | 239 | 12 | 2 | 58 | 69 | 20 | 1 |
| miR156c,g |  |  |  |  |  |  |  |  |  |  |  |  |  | GCTCACTTCTCTCTCTGTCAGC | 11 | 8 | 3 | 1 | 53 | 130 | 119 | 108 | 56 | 160 | 235 | 171 |
| miR156d |  |  |  |  |  |  |  |  |  |  |  |  |  | GCTCACTGCTCTTTCTGTCATC | 1 | 0 | 0 | 0 | 1 | 1 | 10 | 1 | 0 | 1 | 15 | 1 |
| miR156e |  |  |  |  |  |  |  |  |  |  |  |  |  | GCTCACTGCTCTTTCTGTCATC | 1 | 0 | 0 | 0 | 1 | 1 | 10 | 1 | 0 | 1 | 15 | 1 |
| miR156f |  |  |  |  |  |  |  |  |  |  |  |  |  | GCTCACTTCTCTTTCTGTCAGC | 2 | 3 | 1 | 1 | 22 | 16 | 137 | 19 | 17 | 288 | 191 | 33 |
| miR156h,j |  |  |  |  |  |  |  |  |  |  |  |  |  | GCTCGCTCCTCTTTCTGTCAGC | 5 | 3 | 10 | 3 | 12 | 25 | 72 | 18 | 18 | 22 | 98 | 32 |
| miR156i |  |  |  |  |  |  |  |  |  |  |  |  |  | GCTCACTGCTCTGTCTGTCATC | 2 | 2 | 2 | 1 | 4 | 12 | 30 | 7 | 4 | 7 | 41 | 9 |
| miR156k | TGACAGAAGAGAGAGAGCACA | 1 | 0 | 0 | 0 | 0 | 0 | 0 | 0 | 0 | 0 | 0 | 0 | TGCTCTCTGATCTATCTGTCATT | 0 | 0 | 0 | 0 | 0 | 0 | 0 | 0 | 0 | 0 | 0 | 0 |
| miR156l | CGACAGAAGAGAGTGAGCATA | 1 | 0 | 0 | 0 | 0 | 0 | 0 | 0 | 0 | 0 | 0 | 0 |  |  |  |  |  |  |  |  |  |  |  |  |  |
| miR159a.1 | TTTGGATTGAAGGGAGCTCTG | 45 | 19 | 14 | 17 | 9938 | 24225 | 1348 | 9270 | 12626 | 4655 | 2057 | 14285 | GAGCTCCTTTCGGTCCAAAA | 1 | 0 | 12 | 0 | 19 | 11 | 1199 | 16 | 24 | 8 | 1255 | 11 |
| miR159a.2 | TTGCATGCCCCAGGAGCTGCA | 15 | 7 | 4 | 6 | 1 | 0 | 2 | 3 | 0 | 2 | 2 | 3 | AGCTGCTGGGTCATGGATC | 7 | 2 | 109 | 2 | 13 | 4 | 170 | 20 | 1 | 2 | 266 | 2 |
| miR159b |  |  |  |  |  |  |  |  |  |  |  |  |  | AGCTGCTTGTTCATGGTTCC | 0 | 0 | 6 | 0 | 0 | 1 | 6 | 0 | 0 | 0 | 10 | 0 |
| miR159c | ATTGGATTGAAGGGAGCTCCA | 0 | 0 | 0 | 0 | 0 | 0 | 0 | 0 | 0 | 0 | 0 | 0 | GAGCTCCTTTCGATCCAATTC | 0 | 0 | 0 | 0 | 0 | 0 | 0 | 0 | 0 | 0 | 0 | 0 |
| miR159d | ATTGGATTGAAGGGAGCTCCG | 0 | 0 | 0 | 0 | 0 | 0 | 0 | 0 | 0 | 0 | 0 | 0 |  |  |  |  |  |  |  |  |  |  |  |  |  |
| miR159e | ATTGGATTGAAGGGAGCTCCT | 0 | 0 | 0 | 0 | 0 | 0 | 0 | 0 | 0 | 0 | 0 | 0 | GAGCTCCCTTTCGATCCAATTC | 0 | 0 | 0 | 0 | 0 | 0 | 0 | 0 | 0 | 0 | 0 | 0 |
| miR159f | CTTGGATTGAAGGGAGCTCTA | 0 | 0 | 0 | 0 | 1 | 1 | 0 | 0 | 0 | 0 | 0 | 0 | GAGCTCCCTTCGATCCAATCCA | 0 | 0 | 0 | 0 | 0 | 0 | 0 | 0 | 0 | 0 | 0 | 0 |
| miR160a,b | TGCCTGGCTCCCTGTATGCCA | 26 | 14 | 5 | 8 | 43 | 4 | 4 | 54 | 3 | 9 | 8 | 7 | GCGTGCAAGGAGCCAAGCATG^e^ | 12 | 3 | 85 | 6 | 0 | 0 | 12 | 1 | 0 | 0 | 22 | 0 |
| miR160c |  |  |  |  |  |  |  |  |  |  |  |  |  | GCGTGCACGGAGCCAAGCATA | 2 | 1 | 349 | 1 | 1 | 1 | 142 | 2 | 0 | 0 | 234 | 0 |
| miR160d |  |  |  |  |  |  |  |  |  |  |  |  |  | GCGTGCGAGGAGCCAAGCATG | 0 | 0 | 110 | 0 | 0 | 0 | 4 | 0 | 0 | 0 | 4 | 0 |
| miR160e | TGCCTGGCTCCCTGTATGCCG | 5 | 3 | 1 | 2 | 17 | 3 | 5 | 44 | 2 | 10 | 11 | 5 | GCGTGCGAGGTGCCAAGCATG | 0 | 0 | 5 | 0 | 0 | 0 | 11 | 0 | 0 | 0 | 16 | 0 |
| miR160f | TGCCTGGCTCCCTGAATGCCA | 10 | 13 | 1 | 3 | 11 | 6 | 3 | 35 | 7 | 21 | 5 | 29 | GCATTGAGGGAGTCATGCAGG | 0 | 0 | 1 | 0 | 2 | 0 | 134 | 9 | 0 | 4 | 99 | 1 |
| miR162a | TCGATAAACCTCTGCATCCAG | 79 | 112 | 28 | 53 | 146 | 139 | 85 | 210 | 172 | 192 | 168 | 255 | GGGCGCAGTGGTTTATCGATC | 0 | 1 | 1 | 0 | 0 | 4 | 7 | 0 | 0 | 0 | 1 | 0 |
| miR162b | TCGATAAGCCTCTGCATCCAG | 13 | 14 | 3 | 3 | 4 | 5 | 3 | 9 | 3 | 6 | 9 | 5 |  |  |  |  |  |  |  |  |  |  |  |  |  |
| miR164a | TGGAGAAGCAGGGCACGTGCA | 591 | 426 | 314 | 307 | 351 | 968 | 47 | 674 | 133 | 133 | 71 | 247 | CACGTGGTCTCCTTCTCCATC | 0 | 0 | 0 | 0 | 0 | 0 | 1 | 0 | 0 | 0 | 2 | 0 |
| miR164b |  |  |  |  |  |  |  |  |  |  |  |  |  | CATGTGCCCGTCTTCTCCACC | 1 | 0 | 3 | 0 | 0 | 0 | 8 | 0 | 0 | 0 | 13 | 0 |
| miR164c | TGGAGAAGCAGGGTACGTGCA | 1 | 2 | 0 | 1 | 2 | 6 | 1 | 3 | 4 | 2 | 1 | 3 | CACGTGCTCCCCTTCTCCACC | 0 | 0 | 0 | 0 | 0 | 0 | 0 | 0 | 0 | 0 | 0 | 0 |
| miR164d | TGGAGAAGCAGGGCACGTGCT | 29 | 18 | 23 | 16 | 13 | 73 | 4 | 19 | 1 | 8 | 10 | 2 | CATGTGCGCTCCTTCTCCAGC | 0 | 0 | 4 | 0 | 0 | 0 | 1 | 0 | 0 | 0 | 1 | 0 |
| miR164e | TGGAGAAGCAGGGCACGTGAG | 36 | 15 | 14 | 50 | 1 | 1 | 0 | 0 | 0 | 0 | 0 | 0 | CATGTGTCCGTCTTCTCCACC | 0 | 0 | 0 | 0 | 0 | 0 | 0 | 0 | 0 | 0 | 0 | 0 |
| miR164f |  |  |  |  |  |  |  |  |  |  |  |  |  | CATGTGCCCTTCTTCTCCACC | 0 | 0 | 2 | 0 | 0 | 0 | 3 | 0 | 0 | 0 | 4 | 0 |
| miR166a | TCGGACCAGGCTTCATTCCCC | 5495 | 3982 | 1530 | 3894 | 376 | 332 | 233 | 428 | 281 | 326 | 402 | 426 | GGAATGTTGTCTGGTTCAAGG | 1 | 0 | 5 | 0 | 14 | 13 | 506 | 9 | 22 | 10 | 497 | 13 |
| miR166b |  |  |  |  |  |  |  |  |  |  |  |  |  | GGAATGTTGTCTGGCTCGGGG^e^ | 5 | 2 | 26 | 2 | 3 | 3 | 156 | 3 | 4 | 2 | 127 | 3 |
| miR166c |  |  |  |  |  |  |  |  |  |  |  |  |  | GGAATGTTGTCTGGTCCGAG | 0 | 0 | 19 | 0 | 0 | 0 | 25 | 0 | 0 | 0 | 22 | 0 |
| miR166d |  |  |  |  |  |  |  |  |  |  |  |  |  | GGAATGTTGTCTGGCTCGAGG^e^ | 7 | 5 | 50 | 6 | 6 | 6 | 124 | 5 | 7 | 4 | 122 | 9 |
| miR166f |  |  |  |  |  |  |  |  |  |  |  |  |  | GGAATGTCGTCTGGCCTGAGA | 0 | 0 | 0 | 0 | 0 | 0 | 1 | 0 | 0 | 0 | 2 | 0 |
| miR166n |  |  |  |  |  |  |  |  |  |  |  |  |  | GAATGACGTCCGGTCTGAAGA^e^ | 1 | 2 | 5 | 1 | 3 | 5 | 228 | 21 | 4 | 23 | 138 | 32 |
| miR166e | TCGAACCAGGCTTCATTCCCC | 5 | 3 | 2 | 6 | 1 | 0 | 1 | 1 | 0 | 1 | 1 | 0 | GGAATGTTGTCTGGTTCAAGG | 1 | 0 | 5 | 0 | 14 | 13 | 506 | 9 | 22 | 10 | 497 | 13 |
| miR166g | TCGGACCAGGCTTCATTCCTC | 671 | 562 | 211 | 351 | 66 | 74 | 36 | 64 | 59 | 37 | 58 | 70 | AATGGAGGCTGATCCAAGATC | 0 | 0 | 4 | 0 | 3 | 1 | 214 | 3 | 3 | 3 | 201 | 3 |
| miR166h |  |  |  |  |  |  |  |  |  |  |  |  |  | GGAATGTTGGCTGGCTCGAGG | 3 | 4 | 10 | 1 | 1 | 0 | 22 | 2 | 1 | 0 | 23 | 0 |
| miR166i | TCGGATCAGGCTTCATTCCTC | 2 | 1 | 1 | 1 | 0 | 0 | 0 | 1 | 0 | 0 | 0 | 0 | GGAATGCAGTTTGATCCAAGA | 0 | 0 | 0 | 0 | 0 | 0 | 0 | 0 | 0 | 0 | 0 | 0 |
| miR166j |  |  |  |  |  |  |  |  |  |  |  |  |  | GGAATGCAGTTTGATCCAAGA | 0 | 0 | 0 | 0 | 0 | 0 | 0 | 0 | 0 | 0 | 0 | 0 |
| miR166k | TCGGACCAGGCTTCAATCCCT | 470 | 408 | 280 | 240 | 42 | 70 | 42 | 66 | 46 | 43 | 53 | 77 | GGTTTGTTGTCTGGCTCGAGG | 0 | 0 | 13 | 1 | 1 | 0 | 35 | 1 | 1 | 0 | 32 | 0 |
| miR166l |  |  |  |  |  |  |  |  |  |  |  |  |  | GGATTGTTGTCTGGTTCAAGG | 0 | 0 | 0 | 0 | 5 | 2 | 243 | 2 | 3 | 1 | 308 | 2 |
| miR166m | TCGGACCAGGCTTCATTCCCT | 45 | 20 | 8 | 23 | 1 | 1 | 1 | 1 | 1 | 1 | 0 | 1 | GGTTTGTTGTCTGGTTCAAGG | 0 | 0 | 0 | 0 | 0 | 0 | 0 | 0 | 0 | 0 | 0 | 0 |
| miR166n |  |  |  |  |  |  |  |  |  |  |  |  |  | GAATGACGTCCGGTCTGAAGA | 0 | 0 | 0 | 0 | 0 | 0 | 0 | 0 | 0 | 0 | 0 | 0 |
| miR167a | TGAAGCTGCCAGCATGATCTA | 913 | 1352 | 369 | 583 | 157 | 359 | 75 | 1287 | 47 | 604 | 127 | 494 | GATCATGCATGACAGCCTCATT | 0 | 0 | 12 | 1 | 0 | 1 | 15 | 1 | 0 | 0 | 18 | 4 |
| miR167b |  |  |  |  |  |  |  |  |  |  |  |  |  | GATCATGCTGTGACAGTTTCACT | 0 | 0 | 0 | 0 | 0 | 0 | 1 | 0 | 0 | 0 | 1 | 0 |
| miR167c |  |  |  |  |  |  |  |  |  |  |  |  |  | GGTCATGCTGCGGCAGCCTCACT | 1 | 0 | 3 | 0 | 0 | 0 | 16 | 1 | 0 | 1 | 39 | 0 |
| miR167d | TGAAGCTGCCAGCATGATCTG | 1393 | 1057 | 547 | 467 | 34 | 91 | 45 | 93 | 16 | 64 | 81 | 39 | GATCATGCTGTGCAGTTTCATC | 1 | 0 | 1 | 0 | 7 | 9 | 38 | 31 | 2 | 9 | 285 | 11 |
| miR167e,i |  |  |  |  |  |  |  |  |  |  |  |  |  | AGATCATGTTGCAGCTTCACT | 28 | 25 | 43 | 13 | 6 | 11 | 60 | 11 | 4 | 2 | 92 | 9 |
| miR167f |  |  |  |  |  |  |  |  |  |  |  |  |  | AGATCATCTGGCAGTTTCATC | 4 | 1 | 1 | 1 | 1 | 4 | 7 | 1 | 0 | 0 | 12 | 0 |
| miR167g |  |  |  |  |  |  |  |  |  |  |  |  |  | AGATCATCCGGCAGCTTCATC | 5 | 3 | 8 | 2 | 0 | 2 | 5 | 1 | 0 | 0 | 9 | 0 |
| miR167h |  |  |  |  |  |  |  |  |  |  |  |  |  | AGGTCATGCTGTAGTTTCATC | 2 | 0 | 14 | 1 | 5 | 4 | 181 | 8 | 7 | 6 | 197 | 22 |
| miR167j |  |  |  |  |  |  |  |  |  |  |  |  |  | GATCGTGCTGCGCAGTTTCATC | 1 | 0 | 8 | 0 | 0 | 0 | 4 | 0 | 0 | 0 | 6 | 0 |
| miR168a | TCGCTTGGTGCAGATCGGGAC | 106022 | 79287 | 139307 | 83166 | 5179 | 9222 | 8385 | 7898 | 6767 | 3421 | 8091 | 12450 | CCCGCCTTGCACCAAGTGAAT | 20 | 34 | 21 | 20 | 102 | 116 | 307 | 134 | 13 | 48 | 457 | 20 |
| miR168b | AGGCTTGGTGCAGCTCGGGAA | 0 | 0 | 0 | 0 | 0 | 0 | 0 | 0 | 0 | 0 | 0 | 0 | CTGTGTTGTGGCATTCCTCCT | 0 | 0 | 0 | 0 | 0 | 0 | 0 | 0 | 0 | 0 | 0 | 0 |
| miR169a | CAGCCAAGGATGACTTGCCGA | 10 | 21 | 6 | 17 | 70 | 146 | 29 | 55 | 53 | 45 | 29 | 39 | GGCAAGTTGTTCTTGGCTACA | 0 | 0 | 0 | 0 | 1 | 2 | 1 | 0 | 0 | 1 | 0 | 0 |
| miR169b | CAGCCAAGGATGACTTGCCGG | 5 | 13 | 5 | 10 | 95 | 35 | 152 | 758 | 27 | 940 | 141 | 249 | GGCAAGTTGTCCTTGGCTACG | 0 | 0 | 0 | 0 | 0 | 0 | 0 | 0 | 0 | 0 | 0 | 0 |
| miR169c |  |  |  |  |  |  |  |  |  |  |  |  |  | GGCAAGTCTGTCCTTGGCTACA | 0 | 0 | 0 | 0 | 0 | 0 | 0 | 0 | 0 | 0 | 0 | 0 |
| miR169d | TAGCCAAGGATGAATTGCCGG | 0 | 0 | 0 | 0 | 0 | 0 | 0 | 0 | 0 | 0 | 0 | 0 | GGCAAGTCATTTCAGGCTACA | 0 | 0 | 0 | 0 | 0 | 0 | 0 | 0 | 0 | 0 | 0 | 0 |
| miR169e | TAGCCAAGGATGACTTGCCGG | 0 | 0 | 0 | 0 | 0 | 2 | 1 | 3 | 1 | 1 | 0 | 1 | GGCAAGTTATTTTCTTTGGCTAC | 0 | 0 | 0 | 0 | 0 | 0 | 0 | 0 | 0 | 0 | 0 | 0 |
| miR169f | TAGCCAAGGATGACTTGCCTA | 0 | 0 | 0 | 1 | 49 | 11 | 78 | 641 | 7 | 21 | 93 | 89 | GGCATGTCTTCCTTGGCTATT | 0 | 0 | 0 | 0 | 0 | 0 | 0 | 0 | 0 | 0 | 0 | 0 |
| miR169g |  |  |  |  |  |  |  |  |  |  |  |  |  | GGCAGTCTCCTTGGCTAGCC | 0 | 0 | 0 | 0 | 0 | 0 | 0 | 0 | 0 | 0 | 0 | 0 |
| miR169h,j,l,m | TAGCCAAGGATGACTTGCCTG | 4 | 6 | 0 | 4 | 64 | 60 | 25 | 342 | 8 | 35 | 32 | 31 |  |  |  |  |  |  |  |  |  |  |  |  |  |
| miR169i,k |  |  |  |  |  |  |  |  |  |  |  |  |  | GGCAGTCTCCTTGGCTAGT | 1 | 1 | 1 | 2 | 48 | 11 | 4 | 12 | 20 | 3 | 1 | 4 |
| miR169n.o | TAGCCAAGAATGACTTGCCTA | 0 | 1 | 0 | 2 | 11 | 2 | 43 | 241 | 6 | 50 | 53 | 93 | GGCCGGTCTTCTTGGCTAGCC | 0 | 0 | 0 | 0 | 0 | 0 | 0 | 0 | 0 | 0 | 0 | 0 |
| miR169p | TAGCCAAGGACAAACTTGCCGG | 0 | 0 | 0 | 0 | 0 | 0 | 0 | 0 | 0 | 0 | 0 | 0 | GGCAAGTCATCCTTGGCTGCA | 0 | 0 | 0 | 0 | 0 | 0 | 0 | 0 | 0 | 0 | 0 | 0 |
| miR169q | TAGCCAAGGAGACTGCCCATG | 0 | 0 | 0 | 0 | 0 | 0 | 0 | 0 | 0 | 0 | 0 | 0 | CAGGCAAGTCATCCTTGGCTAT | 0 | 0 | 0 | 0 | 0 | 0 | 0 | 0 | 0 | 0 | 0 | 0 |
| miR171a | TGATTGAGCCGCGCCAATATC | 0 | 0 | 0 | 0 | 0 | 0 | 1 | 0 | 0 | 1 | 1 | 0 | TATTGGTGAGGTTCAATCCGA | 0 | 0 | 0 | 0 | 0 | 0 | 0 | 0 | 0 | 0 | 0 | 0 |
| miR171b | TGATTGAGCCGTGCCAATATC | 104 | 54 | 29 | 27 | 40 | 35 | 390 | 183 | 6 | 342 | 311 | 25 | TATTGGGGCGGTTCAATCAGA | 0 | 0 | 0 | 0 | 0 | 0 | 0 | 0 | 0 | 0 | 0 | 0 |
| miR171c |  |  |  |  |  |  |  |  |  |  |  |  |  | GGATATTGGTGCGGTTCAATC | 1 | 1 | 15 | 1 | 0 | 0 | 18 | 1 | 0 | 0 | 16 | 1 |
| miR171d |  |  |  |  |  |  |  |  |  |  |  |  |  | TGTTGGCCCGGCTCACTCAGA^e^ | 0 | 0 | 108 | 1 | 0 | 0 | 60 | 1 | 0 | 0 | 109 | 0 |
| miR171e |  |  |  |  |  |  |  |  |  |  |  |  |  | TGTTGGCTCGGCTCACTCAGA^e^ | 1 | 0 | 48 | 1 | 2 | 1 | 678 | 1 | 2 | 1 | 803 | 1 |
| miR171f |  |  |  |  |  |  |  |  |  |  |  |  |  | TGTTGGCATGGTTCAATCAAA^e^ | 1 | 1 | 0 | 1 | 13 | 40 | 242 | 51 | 4 | 30 | 326 | 14 |
| miR171g | GAGGTGAGCCGAGCCAATATC | 0 | 0 | 0 | 0 | 0 | 0 | 0 | 0 | 0 | 0 | 0 | 0 | TATTGACTTGGCTCATCTCAG | 0 | 0 | 0 | 0 | 0 | 0 | 0 | 0 | 0 | 0 | 0 | 0 |
| miR171h | GTGAGCCGAACCAATATCACT | 0 | 0 | 0 | 0 | 0 | 0 | 0 | 0 | 0 | 0 | 0 | 0 | TTTGGTATTGTTTCGGCTCAT | 0 | 0 | 13 | 0 | 0 | 0 | 1 | 0 | 0 | 0 | 1 | 0 |
| miR171i | GGATTGAGCCGCGTCAATATC | 0 | 0 | 0 | 0 | 0 | 0 | 0 | 0 | 0 | 0 | 0 | 0 | AGGTATTGGCGTGCCTCAATC | 0 | 0 | 138 | 1 | 1 | 1 | 92 | 0 | 0 | 0 | 104 | 0 |
| miR172a |  |  |  |  |  |  |  |  |  |  |  |  |  | GTGGCATCATCAAGATTCACA | 0 | 0 | 0 | 0 | 0 | 0 | 0 | 0 | 0 | 0 | 0 | 0 |
| miR172b | GGAATCTTGATGATGCTGCAT | 1 | 0 | 0 | 0 | 0 | 2 | 0 | 1 | 0 | 1 | 1 | 1 |  |  |  |  |  |  |  |  |  |  |  |  |  |
| miR172c | TGAATCTTGATGATGCTGCAC | 1 | 2 | 4 | 3 | 1 | 2 | 0 | 0 | 0 | 0 | 0 | 0 | GCAGCGTCATCAAGATTCACG | 0 | 0 | 0 | 0 | 0 | 0 | 0 | 0 | 0 | 0 | 0 | 0 |
| miR172d | AGAATCTTGATGATGCTGCAT | 1318 | 995 | 557 | 650 | 173 | 742 | 150 | 329 | 29 | 96 | 311 | 78 | GCAGCACCATCAAGATTCAC | 2 | 1 | 20 | 1 | 0 | 1 | 12 | 0 | 0 | 0 | 11 | 0 |
| miR1846a,b,c-3p | TGACCCCGTTCTCCTCGCCGG | 0 | 0 | 0 | 0 | 0 | 0 | 0 | 0 | 0 | 0 | 0 | 0 | - |  |  |  |  |  |  |  |  |  |  |  |  |
| miR1846a,b,c-5p | AGTGAGGAGGCCGGGGCCGCT | 0 | 0 | 1 | 0 | 0 | 0 | 0 | 0 | 0 | 0 | 0 | 0 | - |  |  |  |  |  |  |  |  |  |  |  |  |
| miR1847.1 | TGCAGTTTGCAGTTGTGGCAC | 0 | 0 | 0 | 1 | 0 | 0 | 0 | 0 | 0 | 0 | 0 | 0 | GCCACAACTGCAAACTACATA | 0 | 0 | 0 | 0 | 0 | 0 | 0 | 0 | 0 | 0 | 0 | 0 |
| miR1847.2 | TGGCCCACATGTTAGTGCCACAAC | 0 | 0 | 0 | 0 | 0 | 0 | 0 | 0 | 0 | 0 | 0 | 0 | TGTGGCACTGACATATGGGCCAGA | 2 | 1 | 0 | 1 | 3 | 2 | 4 | 10 | 3 | 8 | 4 | 6 |
| miR1848 | CCTCGCCGGCGCGCGCGTGCA | 0 | 0 | 0 | 0 | 0 | 0 | 0 | 0 | 0 | 0 | 0 | 1 | TGCGCGCGGCCGTCGAGTGG | 0 | 0 | 0 | 0 | 0 | 0 | 0 | 0 | 0 | 0 | 0 | 0 |
| miR1849 | TATCGTATCCTAGGTTGGTTT | 2 | 1 | 0 | 1 | 2 | 3 | 1 | 4 | 2 | 3 | 1 | 3 | GCTAACCTAGGATATGATGGG | 0 | 0 | 0 | 0 | 0 | 0 | 0 | 0 | 0 | 0 | 0 | 0 |
| miR1850.1 | TGGAAAGTTGGGAGATTGGGG | 28 | 76 | 71 | 40 | 5 | 19 | 42 | 19 | 7 | 10 | 35 | 20 | CCAAATTCCCAACTTTTCATC | 0 | 1 | 0 | 0 | 0 | 1 | 1 | 0 | 0 | 0 | 2 | 0 |
| miR1850.2 | TTGTGTGTGAACTAAACGTGG | 0 | 0 | 0 | 0 | 0 | 0 | 0 | 0 | 0 | 0 | 0 | 0 | - |  |  |  |  |  |  |  |  |  |  |  |  |
| miR1850.3 | CTGTTTAGTTCACATCAATCTT | 0 | 0 | 0 | 0 | 0 | 0 | 0 | 0 | 0 | 0 | 0 | 0 | - |  |  |  |  |  |  |  |  |  |  |  |  |
| miR1851 | CGTCTGGGATGGCATTTTGGC | 0 | 2 | 0 | 1 | 0 | 1 | 1 | 1 | 0 | 1 | 1 | 0 | CAAAATGCCATCCCGAACAGA | 0 | 0 | 0 | 0 | 0 | 0 | 0 | 0 | 0 | 0 | 0 | 0 |
| miR1852 | ATATGGATTCAGAATGCAGGT | 0 | 0 | 0 | 0 | 0 | 0 | 0 | 0 | 0 | 0 | 0 | 0 | CTGCATTCAGAATTCATATAG | 0 | 0 | 0 | 0 | 0 | 0 | 0 | 0 | 0 | 0 | 0 | 0 |
| miR1853-3p | TGGTGAAATTTGTAGATTGGA | 0 | 0 | 0 | 0 | 0 | 0 | 0 | 0 | 0 | 0 | 0 | 0 | - |  |  |  |  |  |  |  |  |  |  |  |  |
| miR1853-5p | AGCATTCAAACATTCCCAATTACC | 0 | 0 | 0 | 0 | 0 | 0 | 0 | 0 | 0 | 0 | 0 | 0 | - |  |  |  |  |  |  |  |  |  |  |  |  |
| miR1855 | AGCACTGGAGTAGCCAAGAGA | 0 | 0 | 0 | 0 | 0 | 0 | 0 | 0 | 0 | 0 | 0 | 0 | TCTTGGCCTCCTCTCGCTGCTCC | 0 | 0 | 0 | 0 | 0 | 0 | 0 | 0 | 0 | 0 | 0 | 0 |
| miR1856 | TATGCGTAAGACGGATTCGTA | 7 | 2 | 6 | 5 | 25 | 19 | 8 | 58 | 30 | 26 | 7 | 81 | CGAATCCGTCGGACGCATAGC | 0 | 0 | 1 | 0 | 0 | 0 | 2 | 1 | 0 | 0 | 5 | 2 |
| miR1857-3p | TCATGCTCCAAGAAAACCAGG | 1 | 1 | 0 | 0 | 3 | 4 | 1 | 4 | 5 | 1 | 2 | 5 | - |  |  |  |  |  |  |  |  |  |  |  |  |
| miR1857-5p | TGGTTTTTTTGGAGCATGAGG | 2 | 0 | 1 | 0 | 1 | 0 | 2 | 1 | 0 | 0 | 2 | 0 | - |  |  |  |  |  |  |  |  |  |  |  |  |
| miR1858a,b | GAGAGGAGGACGGAGTGGGGC | 0 | 0 | 0 | 0 | 0 | 0 | 0 | 0 | 0 | 0 | 0 | 0 | CCCAATCCGTCCTCCTCTCCT | 0 | 0 | 0 | 0 | 0 | 0 | 0 | 0 | 0 | 0 | 0 | 0 |
| miR1859 | TTTCCTATGACGTCCATTCCAA | 5 | 10 | 8 | 8 | 4 | 12 | 2 | 1 | 7 | 1 | 2 | 1 | GGAATGGATGCGTAGAGAAAGA | 0 | 0 | 6 | 0 | 0 | 0 | 11 | 0 | 0 | 0 | 8 | 0 |
| miR1860-3p | ATCTGGAAGCTAGGTTTTCTCT | 1 | 1 | 0 | 0 | 0 | 1 | 2 | 1 | 0 | 2 | 5 | 1 | - |  |  |  |  |  |  |  |  |  |  |  |  |
| miR1860-5p | AGAAAACCAGCTTCCAGATCT | 1 | 1 | 0 | 0 | 0 | 1 | 1 | 0 | 0 | 0 | 2 | 0 | - |  |  |  |  |  |  |  |  |  |  |  |  |
| miR1861a | TGATCTTGAGGCAGAAACTGAG | 2 | 0 | 0 | 2 | 0 | 0 | 0 | 0 | 0 | 0 | 0 | 0 | CGGTTCCTGTCCCAAGATCGAG | 1 | 0 | 0 | 0 | 1 | 1 | 1 | 0 | 0 | 0 | 0 | 0 |
| miR1861b,f,i | CGATCTTGAGGCAGGAACTGAG | 2 | 0 | 0 | 2 | 1 | 0 | 0 | 1 | 0 | 0 | 0 | 0 |  |  |  |  |  |  |  |  |  |  |  |  |  |
| miR1861c | CGATCTTGTAGCAAGAACTGAG | 0 | 0 | 0 | 0 | 0 | 0 | 1 | 0 | 0 | 0 | 1 | 1 | CGGTTCTTGTCACAAGACCGAG | 0 | 0 | 0 | 0 | 0 | 0 | 0 | 0 | 0 | 0 | 0 | 0 |
| miR1861d | TGGTCTTGAGGCAGGAACTGAG | 0 | 0 | 0 | 0 | 0 | 0 | 0 | 0 | 0 | 0 | 0 | 0 | CGGTTCCTGTCCCAAGACCGAG | 0 | 0 | 0 | 0 | 0 | 0 | 0 | 0 | 0 | 0 | 0 | 0 |
| miR1861e,k,m | CGGTCTTGTGGCAAGAACTGAG | 1 | 0 | 0 | 0 | 0 | 0 | 1 | 1 | 0 | 0 | 1 | 0 |  | 0 | 0 | 0 | 0 | 0 | 0 | 0 | 0 | 0 | 0 | 0 | 0 |
| miR1861g | CGATCTTGAGGCAGGAACTGAG | 2 | 0 | 0 | 2 | 1 | 0 | 0 | 1 | 0 | 0 | 0 | 0 | CGGTTCCTGTCCCAAGATCGGG | 0 | 0 | 0 | 0 | 0 | 0 | 0 | 0 | 0 | 0 | 0 | 0 |
| miR1861h | CGGTCTTGAGGCAGGAACTGAG | 6 | 2 | 2 | 2 | 5 | 4 | 1 | 6 | 3 | 3 | 2 | 5 | CGGTTCCTGTCCCAAGACTGAG | 1 | 0 | 0 | 0 | 1 | 0 | 1 | 1 | 0 | 1 | 1 | 0 |
| miR1861j |  |  |  |  |  |  |  |  |  |  |  |  |  | CAGTTCCTGTCCCAAGGCTAAG | 0 | 0 | 0 | 0 | 0 | 0 | 0 | 0 | 0 | 0 | 0 | 0 |
| miR1861n | CGATCTTGTGGCAGGAGCTGAG | 0 | 1 | 1 | 3 | 0 | 0 | 0 | 0 | 0 | 0 | 1 | 0 |  |  |  |  |  |  |  |  |  |  |  |  |  |
| miR1862a |  |  |  |  |  |  |  |  |  |  |  |  |  | TCCCAAAATAAATTAATCTTATAC | 0 | 0 | 0 | 0 | 0 | 0 | 0 | 0 | 0 | 0 | 0 | 0 |
| miR1862c |  |  |  |  |  |  |  |  |  |  |  |  |  | TCCCAAAATAAGTCAATCCCGTAC | 0 | 0 | 0 | 0 | 0 | 0 | 1 | 0 | 0 | 0 | 0 | 0 |
| miR1862d | ACTAGGTTTGTTTATTTTGGGACG | 42 | 39 | 11 | 22 | 56 | 87 | 27 | 66 | 31 | 69 | 32 | 42 | TCCCAAAATAAACAAAGCTAGTAC | 0 | 1 | 0 | 0 | 0 | 0 | 2 | 0 | 0 | 0 | 3 | 0 |
| miR1862e | CTAGATTTGTTTATTTTGGGACGG | 105 | 147 | 173 | 99 | 10 | 12 | 4 | 14 | 10 | 9 | 6 | 13 | ATCCCAAAATGAAAAAATCTAGTA | 0 | 1 | 1 | 0 | 14 | 22 | 8 | 10 | 18 | 13 | 7 | 12 |
| miR1863 | AGCTCTGATACCATGTTAGATTAG | 107 | 119 | 11 | 48 | 296 | 850 | 167 | 604 | 44 | 426 | 227 | 91 | AGTCTAATATGGTATCCGAGCTTA | 0 | 0 | 0 | 0 | 17 | 10 | 5 | 9 | 3 | 5 | 13 | 2 |
| miR1864 | TTGTAGTAACGTGATGGTCAATGT | 10 | 12 | 4 | 8 | 1 | 2 | 0 | 1 | 4 | 3 | 1 | 5 | ATTGACCATCGCGTTACTACGCCA | 0 | 0 | 0 | 0 | 1 | 0 | 0 | 1 | 2 | 0 | 0 | 3 |
| miR1865-3p | CGAAGAATCGCAGTCACTAGTTGT | 1 | 0 | 0 | 1 | 0 | 0 | 0 | 1 | 0 | 1 | 1 | 1 | - |  |  |  |  |  |  |  |  |  |  |  |  |
| miR1865-5p | TGCTAGTGATGGTGATTCTTCGAC | 11 | 11 | 6 | 4 | 2 | 1 | 1 | 2 | 0 | 1 | 1 | 1 | - |  |  |  |  |  |  |  |  |  |  |  |  |
| miR1866-3p | TGAAATTCCTGTAAAATTCTTG | 0 | 0 | 0 | 0 | 0 | 0 | 0 | 0 | 0 | 0 | 0 | 0 | - |  |  |  |  |  |  |  |  |  |  |  |  |
| miR1866-5p | GAGGGATTTTGCGGGAATTTCACG | 0 | 0 | 0 | 0 | 0 | 0 | 0 | 0 | 0 | 0 | 0 | 0 | - |  |  |  |  |  |  |  |  |  |  |  |  |
| miR1867 | TTTTTTTTCTAGGACAGAGGGAGT | 66 | 63 | 45 | 61 | 63 | 100 | 33 | 79 | 96 | 68 | 47 | 119 | TCCCTCTATCCCAGAAAAAAACA | 0 | 1 | 0 | 0 | 0 | 1 | 0 | 1 | 0 | 1 | 2 | 0 |
| miR1868 | TCACGGAAAACGAGGGAGCAGCCA | 29 | 21 | 20 | 23 | 0 | 0 | 0 | 1 | 0 | 1 | 1 | 0 | GCTACTTCCTCGTTTTCCGTAAAC | 0 | 0 | 0 | 0 | 0 | 0 | 0 | 0 | 0 | 0 | 0 | 0 |
| miR1869 | TGAGAACAATAGGCATGGGAGGTA | 0 | 0 | 0 | 0 | 0 | 0 | 0 | 0 | 0 | 0 | 0 | 0 | GTTCCCATGCCTAATGCTCTTAGG | 0 | 0 | 0 | 0 | 0 | 0 | 0 | 0 | 0 | 0 | 0 | 0 |
| miR1870 | TGCTGAATTAGACCTAGTGGGCAT | 79 | 120 | 283 | 161 | 5 | 4 | 9 | 4 | 6 | 6 | 8 | 6 | GCCCTTTAGGGCTAATTCAGCATG | 1 | 0 | 0 | 0 | 0 | 0 | 0 | 0 | 0 | 0 | 0 | 0 |
| miR1871 | ATGGCTCTGATATCATGTTGGTTT | 3 | 1 | 0 | 3 | 100 | 239 | 14 | 73 | 148 | 71 | 31 | 129 | TCTAACATGGTATCGGATCCATAG | 4 | 1 | 0 | 1 | 4 | 11 | 1 | 7 | 3 | 2 | 4 | 3 |
| miR1872 | GAACTGTAAGTCTGTGACGGGTAA | 1 | 1 | 0 | 1 | 0 | 0 | 0 | 0 | 0 | 0 | 0 | 0 | GCTTGTGGAGACTTATGGTTGAA | 0 | 0 | 0 | 0 | 0 | 0 | 0 | 0 | 0 | 0 | 0 | 0 |
| miR1873 | TCAACATGGTATCAGAGCTGGAAG | 27 | 24 | 16 | 18 | 24 | 49 | 12 | 35 | 16 | 17 | 20 | 18 | TCTAGCTCTGATACCATGTTGAGT | 1 | 1 | 0 | 0 | 11 | 25 | 8 | 12 | 17 | 11 | 17 | 19 |
| miR1874-3p | TATGGATGGAGGTGTAACCCGATG | 0 | 0 | 0 | 0 | 2 | 1 | 1 | 1 | 0 | 1 | 3 | 0 | - |  |  |  |  |  |  |  |  |  |  |  |  |
| miR1874-5p | TAGGGCTACTACACCATCCATAAG | 0 | 0 | 0 | 0 | 0 | 0 | 0 | 0 | 0 | 0 | 0 | 0 | - |  |  |  |  |  |  |  |  |  |  |  |  |
| miR1875 | ACAATGGAGTGAAGTGCAACAGAA | 1 | 0 | 0 | 1 | 0 | 1 | 0 | 1 | 0 | 0 | 0 | 0 | TCATTGCACTTTGCTCCATTGTGG | 0 | 0 | 0 | 0 | 0 | 0 | 0 | 0 | 0 | 0 | 0 | 0 |
| miR1876 | ATAAGTGGGTTTGTGGGCTGGCCC | 14 | 17 | 2 | 8 | 15 | 23 | 14 | 28 | 11 | 21 | 10 | 24 | GCTGGCTTTTGAACCCATTTATGG | 0 | 0 | 0 | 0 | 1 | 0 | 1 | 1 | 0 | 0 | 1 | 1 |
| miR1877 | AGATGACATGTGAATGATGAGGGG | 0 | 0 | 0 | 0 | 0 | 0 | 0 | 0 | 0 | 0 | 0 | 0 | TCTCATTGCTTGCATGTCATCCAA | 0 | 0 | 0 | 0 | 0 | 0 | 0 | 0 | 0 | 0 | 0 | 0 |
| miR1878 | ACTTAATCTGGACACTATAAAAGA | 9 | 29 | 2 | 5 | 10 | 15 | 1 | 13 | 14 | 17 | 2 | 26 | ATTTGTAGTGTTCAGATTGAGTTT | 2 | 2 | 1 | 1 | 49 | 125 | 17 | 70 | 58 | 67 | 17 | 101 |
| miR1879 | GTGTTTGGTTTAGGGATGAGGTGG | 9 | 11 | 2 | 6 | 9 | 10 | 6 | 10 | 3 | 7 | 4 | 2 | ACCTCGTCCCCAAACCAAACACAT | 0 | 0 | 0 | 0 | 0 | 0 | 0 | 0 | 0 | 0 | 0 | 0 |
| miR1880 | TTCCAAGCGGGCCACTTAAGCATT | 1 | 2 | 1 | 1 | 0 | 0 | 1 | 0 | 0 | 0 | 1 | 0 | CGCTTAAGTGGTCCGCCTGGATAA | 0 | 1 | 0 | 0 | 1 | 1 | 0 | 1 | 0 | 0 | 0 | 1 |
| miR1881 | AATGTTATTGTAGCGTGGTGGTGT | 0 | 0 | 0 | 0 | 0 | 0 | 0 | 0 | 0 | 0 | 0 | 0 | ACCACTACGCTACAATAACGTTGA | 0 | 0 | 0 | 0 | 0 | 0 | 0 | 0 | 0 | 0 | 0 | 0 |
| miR1882a | AGATTGCTTTCAAGGTCATTTCTT | 8 | 5 | 3 | 2 | 27 | 87 | 8 | 23 | 62 | 23 | 22 | 72 | GAAATGATCTTGGATGCAATCTAG | 0 | 0 | 0 | 0 | 0 | 0 | 0 | 0 | 0 | 0 | 0 | 0 |
| miR1882b,c,d,g,h |  |  |  |  |  |  |  |  |  |  |  |  |  | GAAATGATCTTGGACGCAATCTAG | 0 | 0 | 0 | 0 | 0 | 1 | 0 | 0 | 0 | 0 | 0 | 0 |
| miR1882e |  |  |  |  |  |  |  |  |  |  |  |  |  | GAAATGATCTTGGACGTAATCTAG | 20 | 15 | 5 | 14 | 118 | 256 | 44 | 139 | 126 | 48 | 27 | 143 |
| miR1882f |  |  |  |  |  |  |  |  |  |  |  |  |  | GAAATGATCTTGGATGCAATCTTG | 0 | 0 | 0 | 0 | 0 | 0 | 0 | 0 | 0 | 0 | 0 | 0 |
| miR1883a | ACCTGTGACGGGCCGAGAATGGAA | 7 | 12 | 2 | 4 | 5 | 8 | 11 | 8 | 5 | 11 | 7 | 14 | GGGTTCCATTCTCGATCCGTCACA | 23 | 28 | 25 | 25 | 0 | 0 | 1 | 1 | 0 | 1 | 1 | 0 |
| miR1883b |  |  |  |  |  |  |  |  |  |  |  |  |  | CTACTTCCTGCCCATCACAGGTGA | 0 | 0 | 0 | 0 | 0 | 0 | 0 | 0 | 0 | 0 | 0 | 0 |
| miR1884a | TGTGACGCCGTTGACTTTTCAT | 1 | 2 | 0 | 0 | 1 | 1 | 0 | 1 | 1 | 2 | 0 | 2 | AAAAAGTCAACGGTGTCATACA | 0 | 0 | 0 | 0 | 0 | 0 | 0 | 0 | 0 | 0 | 0 | 0 |
| miR1884b | AATGTATGACGCTGTTGACTTTTA | 3 | 4 | 2 | 3 | 80 | 146 | 58 | 187 | 127 | 222 | 67 | 267 | AAAGTCAACGGTGTCATATATTTA | 19 | 25 | 20 | 3 | 12 | 13 | 19 | 21 | 2 | 25 | 15 | 3 |
| miR2055 | TTTCCTTGGGAAGGTGGTTTC | 2 | 1 | 2 | 1 | 2 | 1 | 2 | 1 | 0 | 0 | 1 | 0 | AGGCACCAGCCCAAGGAAACA | 0 | 0 | 0 | 0 | 0 | 0 | 0 | 0 | 0 | 0 | 0 | 0 |
| miR2090 | AACTCTGATTCTAGAATTTTTG | 0 | 0 | 0 | 0 | 1 | 1 | 4 | 0 | 0 | 0 | 2 | 1 | AAAATTCCAGAATCGGATCCTT | 0 | 0 | 0 | 0 | 0 | 0 | 0 | 0 | 0 | 0 | 0 | 0 |
| miR2091-3p | CATACATTGCCTCCTAGGCTTG | 0 | 0 | 0 | 0 | 0 | 0 | 0 | 0 | 0 | 0 | 0 | 0 | - |  |  |  |  |  |  |  |  |  |  |  |  |
| miR2091-5p | TCAACCGAGCCGAGGAGGAGG | 0 | 0 | 0 | 0 | 0 | 0 | 0 | 0 | 0 | 0 | 0 | 0 | - | 0 | 0 | 0 | 0 | 0 | 0 | 0 | 0 | 0 | 0 | 0 | 0 |
| miR2092-3p | ACCAGCATTCCATTGGCAGAGG | 0 | 0 | 0 | 0 | 0 | 0 | 0 | 0 | 0 | 0 | 0 | 0 | - |  |  |  |  |  |  |  |  |  |  |  |  |
| miR2092-5p | CAACTGAAGTCGGTGTTTACT | 0 | 0 | 0 | 0 | 0 | 0 | 0 | 0 | 0 | 0 | 0 | 0 | - |  |  |  |  |  |  |  |  |  |  |  |  |
| miR2093-3p | ACATCTTCCAATTAATGCAT | 0 | 0 | 0 | 0 | 0 | 0 | 0 | 0 | 0 | 0 | 0 | 0 | - |  |  |  |  |  |  |  |  |  |  |  |  |
| miR2093-5p | GTGCATTAATTGGAAGAACA | 0 | 0 | 0 | 0 | 0 | 0 | 0 | 0 | 0 | 0 | 0 | 0 | - |  |  |  |  |  |  |  |  |  |  |  |  |
| miR2094-3p | CAGAGCTGTGGCATCCACGTCG | 0 | 0 | 0 | 0 | 0 | 0 | 0 | 0 | 0 | 0 | 0 | 0 | - |  |  |  |  |  |  |  |  |  |  |  |  |
| miR2094-5p | TGGCTGCTAGGCTCCTGGGTG | 0 | 0 | 0 | 0 | 0 | 0 | 0 | 0 | 0 | 0 | 0 | 0 | - |  |  |  |  |  |  |  |  |  |  |  |  |
| miR2095-3p | CTTCCATTTATGATAAGTAT | 0 | 0 | 0 | 0 | 0 | 0 | 0 | 0 | 0 | 0 | 0 | 0 | - |  |  |  |  |  |  |  |  |  |  |  |  |
| miR2095-5p | CTGATAATTTTACGATGAATAG | 0 | 0 | 0 | 0 | 0 | 0 | 0 | 0 | 0 | 0 | 0 | 0 | - |  |  |  |  |  |  |  |  |  |  |  |  |
| miR2096-3p | CCTGAGGGGAAATCGGCGGGA | 0 | 0 | 0 | 0 | 0 | 0 | 0 | 0 | 0 | 0 | 0 | 0 | - |  |  |  |  |  |  |  |  |  |  |  |  |
| miR2096-5p | TGCCGATTTCCCCCTCGGGCG | 0 | 0 | 0 | 0 | 0 | 0 | 0 | 0 | 0 | 0 | 0 | 0 | - |  |  |  |  |  |  |  |  |  |  |  |  |
| miR2097-3p | TTCTCTTCTTCGTGTCGCATTT | 0 | 0 | 0 | 0 | 0 | 0 | 0 | 0 | 0 | 0 | 0 | 0 | - |  |  |  |  |  |  |  |  |  |  |  |  |
| miR2097-5p | AGAGATGGGACGGGCAGGGAAG | 0 | 0 | 0 | 0 | 0 | 0 | 0 | 0 | 0 | 0 | 0 | 0 | - |  |  |  |  |  |  |  |  |  |  |  |  |
| miR2098-3p | CGGTTTGTCAAGCGGAGTGC | 0 | 0 | 0 | 0 | 0 | 0 | 0 | 0 | 0 | 0 | 0 | 0 | - |  |  |  |  |  |  |  |  |  |  |  |  |
| miR2098-5p | TCCCGTGGAGGCAGCCGATG | 0 | 0 | 0 | 0 | 0 | 0 | 0 | 0 | 0 | 0 | 0 | 0 | - |  |  |  |  |  |  |  |  |  |  |  |  |
| miR2099-3p | ACAAAGCTGTAGCGTTATTC | 0 | 0 | 0 | 0 | 0 | 0 | 0 | 0 | 0 | 0 | 0 | 0 | - |  |  |  |  |  |  |  |  |  |  |  |  |
| miR2099-5p | TGAATATGTTTGTACAAGCTTT | 0 | 0 | 0 | 0 | 0 | 0 | 0 | 0 | 0 | 0 | 0 | 0 | - |  |  |  |  |  |  |  |  |  |  |  |  |
| miR2100-3p | AACCGCTGTTTAGGCGGAGTGG | 0 | 0 | 0 | 0 | 0 | 0 | 0 | 0 | 0 | 0 | 0 | 0 | - |  |  |  |  |  |  |  |  |  |  |  |  |
| miR2100-5p | TTCTCTCAAGTTGCCAAACAAG | 0 | 0 | 0 | 0 | 0 | 0 | 0 | 0 | 0 | 0 | 0 | 0 | - |  |  |  |  |  |  |  |  |  |  |  |  |
| miR2101-3p | ATTTAACTCAAGTGAGCATTGT | 0 | 0 | 0 | 0 | 0 | 0 | 0 | 0 | 0 | 0 | 0 | 0 | - |  |  |  |  |  |  |  |  |  |  |  |  |
| miR2101-5p | ACATGTTTACAAGTTAAAATGT | 0 | 0 | 0 | 0 | 0 | 0 | 0 | 0 | 0 | 0 | 0 | 0 |  |  |  |  |  |  |  |  |  |  |  |  |  |
| miR2102-3p | CATGGTGCCGGTTCCGGTGGCG | 0 | 0 | 0 | 0 | 0 | 0 | 0 | 0 | 0 | 0 | 0 | 0 |  |  |  |  |  |  |  |  |  |  |  |  |  |
| miR2102-5p | GGGCAAGCCGCCGCCGCCAC | 0 | 0 | 0 | 0 | 0 | 0 | 0 | 0 | 0 | 0 | 0 | 0 |  |  |  |  |  |  |  |  |  |  |  |  |  |
| miR2103 | TTTCCCTCTCCGTGCGCGCTCG | 0 | 0 | 0 | 0 | 0 | 0 | 0 | 0 | 0 | 0 | 0 | 0 | AGGATGGATGCCGGGAGGGAGAGA | 0 | 0 | 0 | 0 | 0 | 0 | 0 | 0 | 0 | 0 | 0 | 0 |
| miR2104 | GCGGCGAGGGGATGCGAGCGTG | 0 | 0 | 0 | 0 | 0 | 0 | 0 | 0 | 0 | 0 | 0 | 0 | CGGTGGCTTCGACCCTCGTCTCG | 0 | 0 | 0 | 0 | 0 | 0 | 0 | 0 | 0 | 0 | 0 | 0 |
| miR2105 | TTGTGATGTGAATGATTCAT | 0 | 0 | 0 | 0 | 0 | 0 | 0 | 0 | 0 | 0 | 0 | 0 | GATGTATTTCATTAAGAAT | 0 | 0 | 0 | 0 | 0 | 0 | 0 | 0 | 0 | 0 | 0 | 0 |
| miR2106 | CCGAGGTTTTCTGGATACATT | 0 | 0 | 0 | 0 | 0 | 0 | 0 | 0 | 0 | 0 | 0 | 0 | TGTTCCGAGAATCTCGTACGCC | 0 | 0 | 0 | 0 | 0 | 0 | 0 | 0 | 0 | 0 | 0 | 0 |
| miR319a | TTGGACTGAAGGGTGCTCCC | 0 | 0 | 0 | 0 | 6 | 6 | 4 | 0 | 10 | 2 | 5 | 1 | GAGAGCTCTCTTCAGTCCACTC | 0 | 0 | 0 | 0 | 0 | 0 | 0 | 0 | 0 | 0 | 0 | 0 |
| miR319b |  |  |  |  |  |  |  |  |  |  |  |  |  | GAGAGCGTCCTTCAGTCCACTC | 0 | 0 | 0 | 0 | 0 | 0 | 0 | 0 | 0 | 0 | 0 | 0 |
| miR390 | AAGCTCAGGAGGGATAGCGCC | 41 | 41 | 163 | 29 | 30 | 22 | 118 | 30 | 3 | 29 | 169 | 2 | CGCTATCTATCCTGAGCTCC | 0 | 0 | 20 | 0 | 0 | 1 | 50 | 1 | 0 | 1 | 53 | 1 |
| miR393 | TCCAAAGGGATCGCATTGATC | 32 | 22 | 11 | 9 | 473 | 119 | 22 | 1417 | 8 | 55 | 41 | 23 | TCATGCGATCCTTTTGGAGG | 0 | 0 | 0 | 0 | 0 | 0 | 0 | 0 | 0 | 0 | 0 | 0 |
| miR393b |  |  |  |  |  |  |  |  |  |  |  |  |  | TCAGTGCAATCCCTTTGGAAT | 26 | 22 | 11 | 7 | 542 | 1865 | 283 | 969 | 1137 | 614 | 342 | 1933 |
| miR394 | TTGGCATTCTGTCCACCTCC | 14 | 13 | 4 | 5 | 2 | 1 | 5 | 3 | 3 | 4 | 2 | 4 | AGGTGGGCATACTGCCAATG | 0 | 0 | 4 | 0 | 0 | 0 | 3 | 0 | 0 | 0 | 4 | 0 |
| miR395a | GTGAAGTGCTTGGGGGAACTC | 0 | 0 | 0 | 0 | 0 | 0 | 0 | 0 | 0 | 0 | 0 | 0 | GTTCTCCTCAATCCACTTCAG | 0 | 0 | 0 | 0 | 0 | 0 | 0 | 0 | 0 | 0 | 0 | 0 |
| miR395b | GTGAAGTGTTTGGGGGAACTC | 0 | 0 | 0 | 0 | 0 | 0 | 0 | 0 | 0 | 0 | 0 | 0 | GTTCCTTTCAAGCACTTTACGA | 0 | 0 | 0 | 0 | 0 | 0 | 0 | 0 | 0 | 0 | 0 | 0 |
| miR395c | GTGAAGTGTTTGGAGGAACTC | 0 | 0 | 0 | 0 | 0 | 0 | 0 | 0 | 0 | 0 | 0 | 0 | GTTCTCTTTAAGCACTTCATAC | 0 | 0 | 0 | 0 | 0 | 0 | 0 | 0 | 0 | 0 | 0 | 0 |
| miR395d,p,s |  |  |  |  |  |  |  |  |  |  |  |  |  | GTTCCCTTCAAGCACTTCACGT | 0 | 0 | 0 | 0 | 0 | 0 | 0 | 0 | 0 | 0 | 0 | 0 |
| miR395e,r |  |  |  |  |  |  |  |  |  |  |  |  |  | GTTCCCTTCAACCACTTCACGT | 0 | 0 | 0 | 0 | 0 | 0 | 0 | 0 | 0 | 0 | 0 | 0 |
| miR395f | GTGAATTGTTTGGGGGAACTC | 0 | 0 | 0 | 0 | 0 | 0 | 0 | 0 | 0 | 0 | 0 | 0 | GTTCCCTTCAATCACTTCACAT | 0 | 0 | 0 | 0 | 0 | 0 | 0 | 0 | 0 | 0 | 0 | 0 |
| miR395g |  |  |  |  |  |  |  |  |  |  |  |  |  | GTTCCCTTCGAACACTTCACGT | 0 | 0 | 0 | 0 | 0 | 0 | 0 | 0 | 0 | 0 | 0 | 0 |
| miR395h |  |  |  |  |  |  |  |  |  |  |  |  |  | GTTTCCTCAACACACTTCACAT | 0 | 0 | 0 | 0 | 0 | 0 | 0 | 0 | 0 | 0 | 0 | 0 |
| miR395i |  |  |  |  |  |  |  |  |  |  |  |  |  | GTTCTCTTCAAGCACTTCACGT | 0 | 0 | 0 | 0 | 0 | 0 | 0 | 0 | 0 | 0 | 0 | 0 |
| miR395j |  |  |  |  |  |  |  |  |  |  |  |  |  | GTTCCTTGCAAGCACTTCACAT | 0 | 0 | 0 | 0 | 0 | 0 | 0 | 0 | 0 | 0 | 0 | 0 |
| miR395k |  |  |  |  |  |  |  |  |  |  |  |  |  | GTTTCCTTCAAGCACTTCACGT | 0 | 0 | 0 | 0 | 0 | 0 | 0 | 0 | 0 | 0 | 0 | 0 |
| miR395l |  |  |  |  |  |  |  |  |  |  |  |  |  | GTTCCTTCCAAGCACTTCACAC | 0 | 0 | 0 | 0 | 0 | 0 | 0 | 0 | 0 | 0 | 0 | 0 |
| miR395m |  |  |  |  |  |  |  |  |  |  |  |  |  | GTTCTCCTCAAATCACTTCAGTA | 0 | 0 | 0 | 0 | 0 | 0 | 0 | 0 | 0 | 0 | 0 | 0 |
| miR395n |  |  |  |  |  |  |  |  |  |  |  |  |  | GTTCCCTTCAAGCACTTCACGA | 0 | 0 | 0 | 0 | 0 | 0 | 0 | 0 | 0 | 0 | 0 | 0 |
| miR395o | ATGAAGTGTTTGGAGGAACTC | 0 | 0 | 0 | 0 | 0 | 0 | 0 | 0 | 0 | 0 | 0 | 0 |  |  |  |  |  |  |  |  |  |  |  |  |  |
| miR395q |  |  |  |  |  |  |  |  |  |  |  |  |  | GTTCCCTTCAAGCACTTCACAT | 0 | 0 | 0 | 0 | 0 | 0 | 0 | 0 | 0 | 0 | 0 | 0 |
| miR395t | GTGAAGTGTTTGGGGAAACTC | 0 | 0 | 0 | 0 | 0 | 0 | 0 | 0 | 0 | 0 | 0 | 0 | GTTCTCCTCAAACCACTTCAGC | 0 | 0 | 0 | 0 | 0 | 0 | 0 | 0 | 0 | 0 | 0 | 0 |
| miR395u | GTGAAGCGTTTGGGGGAAATC | 0 | 0 | 0 | 0 | 0 | 0 | 0 | 0 | 0 | 0 | 0 | 0 | GTTCCCTTCAAGCACTTCACGA | 0 | 0 | 0 | 0 | 0 | 0 | 0 | 0 | 0 | 0 | 0 | 0 |
| miR395v | GTGAAGTATTTGGCGGAACTC | 0 | 0 | 0 | 0 | 0 | 0 | 0 | 0 | 0 | 0 | 0 | 0 | ATTCTCTTTAAGCACTTCATAC | 0 | 0 | 0 | 0 | 0 | 0 | 0 | 0 | 0 | 0 | 0 | 0 |
| miR395w | GTGAAGTGTTTGGGGGATTCTC | 0 | 0 | 0 | 0 | 0 | 0 | 0 | 0 | 0 | 0 | 0 | 0 | GAGTTCTCTTTAATCATTTCACAT | 0 | 0 | 0 | 0 | 0 | 0 | 0 | 0 | 0 | 0 | 0 | 0 |
| miR396a,b | TTCCACAGCTTTCTTGAACTG | 1 | 1 | 0 | 1 | 12 | 18 | 7 | 11 | 11 | 26 | 8 | 9 | GTTCAATAAAGCTGTGGGAAA^e^ | 0 | 1 | 4 | 0 | 0 | 0 | 109 | 0 | 0 | 0 | 103 | 1 |
| miR396c | TTCCACAGCTTTCTTGAACTT | 5 | 7 | 4 | 1 | 608 | 973 | 328 | 1259 | 604 | 1186 | 608 | 1650 | GGTCAAGAAAGCTGTGGGAAG | 4 | 6 | 35 | 4 | 28 | 8 | 1606 | 16 | 34 | 8 | 1375 | 12 |
| miR396d | TCCACAGGCTTTCTTGAACTG | 470 | 474 | 49 | 140 | 8554 | 14853 | 2139 | 10103 | 10352 | 11223 | 2190 | 16585 | GAAAAGAAAGCTGAATTGTCGA | 0 | 0 | 0 | 0 | 0 | 0 | 0 | 0 | 0 | 0 | 0 | 0 |
| miR396e |  |  |  |  |  |  |  |  |  |  |  |  |  | GTTCAAGAAAGCCCATGGAA | 0 | 0 | 10 | 0 | 24 | 21 | 3819 | 24 | 21 | 11 | 2779 | 9 |
| miR396f |  |  |  |  |  |  |  |  |  |  |  |  |  | GTTCAAGAAAGTCCTTGGAA^c^ | 0 | 0 | 411 | 0 | 470 | 257 | 38230 | 112 | 264 | 93 | 28126 | 54 |
| miR397a | TCATTGAGTGCAGCGTTGATG | 5 | 2 | 2 | 3 | 2 | 32 | 3 | 13 | 2 | 11 | 3 | 15 | TCAGCGCTTCACTCAATCATG | 0 | 0 | 0 | 0 | 0 | 0 | 0 | 0 | 0 | 0 | 0 | 0 |
| miR397b | TTATTGAGTGCAGCGTTGATG | 0 | 0 | 0 | 0 | 0 | 1 | 0 | 0 | 0 | 0 | 0 | 0 | CCAGCACTGCACCCAATCACG | 0 | 0 | 0 | 0 | 0 | 0 | 0 | 0 | 0 | 0 | 0 | 0 |
| miR398a | TGTGTTCTCAGGTCACCCCTT | 0 | 0 | 0 | 0 | 0 | 6 | 1 | 1 | 0 | 0 | 3 | 0 | GGAGTGGTACTGAGAACACAGG | 0 | 0 | 0 | 0 | 0 | 1 | 2 | 6 | 0 | 0 | 2 | 8 |
| miR398b | TGTGTTCTCAGGTCGCCCCTG | 1 | 0 | 0 | 0 | 12 | 51 | 30 | 32 | 2 | 20 | 40 | 6 | GGGCGAGCTGGGAACACACGG | 3 | 4 | 6 | 10 | 1 | 1 | 1 | 3 | 1 | 0 | 1 | 3 |
| miR399a | TGCCAAAGGAGAATTGCCCTG | 0 | 0 | 0 | 0 | 0 | 0 | 1 | 0 | 0 | 2 | 0 | 0 | GGGCAGTTCACCTTTGGCACA | 0 | 0 | 0 | 0 | 0 | 0 | 0 | 0 | 0 | 0 | 0 | 0 |
| miR399b |  |  |  |  |  |  |  |  |  |  |  |  |  | GTGCGATTCTCCTCTGGCATG | 0 | 0 | 0 | 0 | 0 | 0 | 0 | 0 | 0 | 0 | 0 | 0 |
| miR399c |  |  |  |  |  |  |  |  |  |  |  |  |  | GGGCGGTTTCTCCTTTGGCACG | 0 | 0 | 0 | 0 | 0 | 0 | 0 | 0 | 0 | 0 | 0 | 0 |
| miR399d | TGCCAAAGGAGAGTTGCCCTG | 0 | 1 | 0 | 0 | 3 | 1 | 2 | 23 | 0 | 6 | 2 | 1 | GGCAGCTCTCCTCTGGCAGG | 0 | 0 | 0 | 0 | 1 | 0 | 1 | 1 | 0 | 0 | 5 | 2 |
| miR399e |  |  |  |  |  |  |  |  |  |  |  |  |  | GGGTGAGTCTTCCTTGGCAGT | 0 | 0 | 0 | 0 | 0 | 0 | 0 | 0 | 0 | 0 | 0 | 0 |
| miR399f |  |  |  |  |  |  |  |  |  |  |  |  |  | GGGCCATGTCTCCTTGGGCAGA | 0 | 0 | 0 | 0 | 0 | 0 | 0 | 0 | 0 | 0 | 0 | 0 |
| miR399g |  |  |  |  |  |  |  |  |  |  |  |  |  | GGGCAACTACTCCATTGGCAGA | 0 | 0 | 0 | 0 | 0 | 0 | 0 | 0 | 0 | 0 | 0 | 0 |
| miR399h | TGCCAAAGGAGACTTGCCCAG | 0 | 0 | 0 | 0 | 0 | 0 | 0 | 0 | 0 | 0 | 0 | 0 | GGGCAGGTCTCCCTTGGCAGT | 0 | 0 | 0 | 0 | 0 | 0 | 0 | 0 | 0 | 0 | 0 | 0 |
| miR399i | TGCCAAAGGAGAGCTGCCCTG | 0 | 0 | 0 | 0 | 0 | 0 | 0 | 0 | 0 | 0 | 0 | 0 | GTGCAGTTCTCCTCTGGCATG | 0 | 0 | 0 | 0 | 0 | 0 | 0 | 0 | 0 | 0 | 0 | 0 |
| miR399j | TGCCAAAGGAGAGTTGCCCTA | 0 | 0 | 0 | 0 | 0 | 0 | 0 | 5 | 0 | 8 | 1 | 0 | GGGCTCCTCTCTCTTGGCAGG | 0 | 0 | 0 | 0 | 0 | 0 | 0 | 0 | 0 | 0 | 0 | 0 |
| miR399k | TGCCAAAGGAAATTTGCCCCG | 0 | 0 | 0 | 0 | 0 | 0 | 0 | 0 | 0 | 0 | 0 | 0 | GGGCAAGTTGTCCTTTGGCAGA | 0 | 0 | 0 | 0 | 0 | 0 | 0 | 0 | 0 | 0 | 1 | 0 |
| miR408 | CTGCACTGCCTCTTCCCTGGC | 2 | 1 | 1 | 1 | 4 | 3 | 6 | 36 | 3 | 16 | 15 | 29 | CAGGGATGAGGCAGAGCATGG | 13 | 17 | 48 | 41 | 2 | 2 | 44 | 15 | 1 | 12 | 58 | 19 |
| miR413 | CTAGTTTCACTTGTTCTGCAC | 0 | 0 | 0 | 0 | 0 | 0 | 0 | 0 | 0 | 0 | 0 | 0 | GCATTACAAAGAAGTCAGAC | 0 | 0 | 0 | 0 | 0 | 0 | 0 | 0 | 0 | 0 | 0 | 0 |
| miR414 | TCATCCTCATCATCATCGTCC | 0 | 0 | 0 | 0 | 0 | 0 | 0 | 0 | 0 | 0 | 0 | 0 | GGGAAGGGGAGGGGGAGGGGTCGG | 0 | 0 | 0 | 0 | 0 | 0 | 0 | 0 | 0 | 0 | 0 | 0 |
| miR415 | AACAGAACAGAAGCAGAGCAG | 0 | 0 | 0 | 0 | 0 | 0 | 0 | 0 | 0 | 0 | 0 | 0 | - |  |  |  |  |  |  |  |  |  |  |  |  |
| miR416 | TGTTCGTCCGTACACTGTTCA | 0 | 0 | 0 | 0 | 0 | 0 | 0 | 0 | 0 | 0 | 0 | 0 | - |  |  |  |  |  |  |  |  |  |  |  |  |
| miR417 | GAATGTAGTGAATTTGTTCCA | 0 | 0 | 0 | 0 | 0 | 0 | 0 | 0 | 0 | 0 | 0 | 0 | - |  |  |  |  |  |  |  |  |  |  |  |  |
| miR418 | TAATGTGATGATGAAATGACG | 0 | 0 | 0 | 0 | 0 | 0 | 0 | 0 | 0 | 0 | 0 | 0 | CATTCTTATCATCGCACATT | 0 | 0 | 0 | 0 | 0 | 0 | 0 | 0 | 0 | 0 | 0 | 0 |
| miR419 | TGATGAATGCTGACGATGTTG | 0 | 0 | 0 | 0 | 0 | 0 | 0 | 0 | 0 | 0 | 0 | 0 | ATGAATGCTTGTTGTCCTGGA | 0 | 0 | 0 | 0 | 0 | 0 | 0 | 0 | 0 | 0 | 0 | 0 |
| miR420 | TAAATTAATCACGGAAATGAT | 0 | 0 | 0 | 0 | 0 | 0 | 0 | 0 | 0 | 0 | 0 | 0 | CATTTAATGATTAATTTAAT | 0 | 0 | 0 | 0 | 0 | 0 | 0 | 0 | 0 | 0 | 0 | 0 |
| miR426 | TTTTGGAAGTTTGTCCTTACG | 0 | 0 | 0 | 0 | 0 | 0 | 0 | 0 | 0 | 0 | 0 | 0 | - |  |  |  |  |  |  |  |  |  |  |  |  |
| miR435 | TTATCCGGTATTGGAGTTGA | 29 | 19 | 6 | 9 | 39 | 38 | 28 | 64 | 34 | 30 | 21 | 58 | - |  |  |  |  |  |  |  |  |  |  |  |  |
| miR437 | AAAGTTAGAGAAGTTTGACTT | 0 | 0 | 0 | 0 | 0 | 0 | 1 | 0 | 0 | 0 | 0 | 0 | GTCAAAATTCTTTTACTTTGA | 0 | 0 | 0 | 0 | 0 | 0 | 0 | 0 | 0 | 0 | 0 | 0 |
| miR438 | TTCCCACGCGTTATAGTGAAA | 0 | 0 | 0 | 0 | 0 | 0 | 0 | 0 | 0 | 0 | 0 | 0 | TCACTTTAACGCGTGGGATAT | 0 | 0 | 0 | 0 | 0 | 0 | 0 | 0 | 0 | 0 | 0 | 0 |
| miR438a,c,e,f,g,i | TGTCGAACCGCGGTTGTTCGA | 0 | 0 | 0 | 0 | 1 | 3 | 1 | 3 | 4 | 2 | 1 | 5 | GAACTGACGCAGTTCGACATG | 0 | 0 | 0 | 0 | 0 | 0 | 0 | 0 | 0 | 0 | 0 | 0 |
| miR438b |  |  |  |  |  |  |  |  |  |  |  |  |  | GAATTGTGGTTGTTCGATAGG | 0 | 0 | 0 | 0 | 0 | 0 | 0 | 0 | 0 | 0 | 0 | 0 |
| miR438d |  |  |  |  |  |  |  |  |  |  |  |  |  | GAACTCACGTAGTTCGACATG | 0 | 0 | 0 | 0 | 0 | 0 | 0 | 0 | 0 | 0 | 0 | 0 |
| miR438h |  |  |  |  |  |  |  |  |  |  |  |  |  | GAACTCACGCAGTTCGACATG | 0 | 0 | 0 | 0 | 0 | 0 | 0 | 0 | 0 | 0 | 0 | 0 |
| miR438j |  |  |  |  |  |  |  |  |  |  |  |  |  | CAACGGGCAGTGTTCGATAGG | 0 | 0 | 0 | 0 | 0 | 0 | 0 | 0 | 0 | 0 | 0 | 0 |
| miR440 | AGTGTCTCCTGATGATCGGGACAA | 13 | 11 | 5 | 8 | 8 | 8 | 9 | 21 | 4 | 10 | 8 | 11 | GTCCTGATCACTAGGAGACTCTGA | 1 | 0 | 0 | 1 | 1 | 1 | 0 | 0 | 0 | 0 | 0 | 0 |
| miR441a | TACCATCAATATAAATGTGGGAAA | 0 | 0 | 0 | 0 | 0 | 0 | 0 | 0 | 0 | 0 | 0 | 0 | TCACACATTCATATTGATCTAGAT | 0 | 0 | 0 | 0 | 0 | 0 | 0 | 0 | 0 | 0 | 0 | 0 |
| miR441b |  |  |  |  |  |  |  |  |  |  |  |  |  | TTTCATATTCATATTGATGTTAA | 0 | 0 | 0 | 0 | 0 | 0 | 0 | 0 | 0 | 0 | 0 | 0 |
| miR441c | TACCATCAATATAAATGTGGGAAA | 0 | 0 | 0 | 0 | 0 | 0 | 0 | 0 | 0 | 0 | 0 | 0 | TTTCATATTTATATTGATGGTAAT | 0 | 0 | 0 | 0 | 0 | 0 | 0 | 0 | 0 | 0 | 0 | 0 |
| miR442 | TGACGTGTAAATTGCGAGACGAAT | 0 | 0 | 0 | 0 | 0 | 0 | 0 | 0 | 0 | 0 | 0 | 0 | TCGTCTCGCAGTTTACAGACGGATT | 0 | 0 | 0 | 0 | 0 | 0 | 0 | 0 | 0 | 0 | 0 | 0 |
| miR443 | ATCACAATACAATAAATCTGGA | 0 | 0 | 0 | 0 | 0 | 0 | 0 | 0 | 0 | 0 | 0 | 0 | TAGATTTATTGTACTGGGATAT | 0 | 0 | 0 | 0 | 0 | 0 | 0 | 0 | 0 | 0 | 0 | 0 |
| miR444a.1 | TTGCTGCCTCAAGCTTGCTGC | 1 | 0 | 0 | 1 | 0 | 0 | 0 | 0 | 0 | 0 | 0 | 0 | AGCAAGCTAGAGGTGGCAACT | 0 | 0 | 0 | 0 | 0 | 0 | 0 | 0 | 0 | 0 | 0 | 0 |
| miR444a.2 | TGCAGTTGCTGCCTCAAGCTT | 53 | 39 | 18 | 28 | 8 | 12 | 12 | 23 | 3 | 10 | 14 | 6 | GCTAGAGGTGGCAACTGCATA | 10 | 8 | 29 | 12 | 3 | 8 | 2 | 4 | 2 | 1 | 3 | 3 |
| miR444b.1 | TGTTGTCTCAAGCTTGCTGCC | 28 | 19 | 15 | 7 | 75 | 65 | 33 | 180 | 7 | 141 | 62 | 20 | TGACAAGCTTGTGGCAGCAA | 0 | 0 | 0 | 0 | 0 | 0 | 0 | 0 | 0 | 0 | 0 | 0 |
| miR444b.2 | TGCAGTTGTTGTCTCAAGCTT | 471 | 284 | 194 | 98 | 203 | 204 | 199 | 300 | 53 | 321 | 183 | 63 | GCTTGTGGCAGCAACTGCACA | 0 | 0 | 0 | 0 | 6 | 25 | 21 | 25 | 7 | 9 | 18 | 24 |
| miR444c.1 |  |  |  |  |  |  |  |  |  |  |  |  |  | CGGCAAGCTAGAGACAGCAAC | 106 | 143 | 686 | 69 | 27 | 29 | 134 | 53 | 22 | 58 | 194 | 43 |
| miR444c.2 |  |  |  |  |  |  |  |  |  |  |  |  |  | GCTAGAGACAGCAACTGCATA | 0 | 1 | 1 | 1 | 0 | 0 | 1 | 0 | 0 | 0 | 0 | 1 |
| miR444d.1 |  |  |  |  |  |  |  |  |  |  |  |  |  | CTTGCAAGAAAGGCACAAAA | 0 | 0 | 0 | 0 | 0 | 0 | 0 | 0 | 0 | 0 | 0 | 0 |
| miR444d.2 |  |  |  |  |  |  |  |  |  |  |  |  |  | GCATGAGGCAACAACTGCATT | 1 | 1 | 0 | 0 | 0 | 0 | 2 | 1 | 0 | 0 | 2 | 0 |
| miR444d.3 | TTGTGGCTTTCTTGCAAGTTG | 1 | 1 | 1 | 1 | 50 | 41 | 24 | 109 | 35 | 77 | 23 | 76 | ACTTGCAAGAAAGGCACAAAA | 0 | 0 | 0 | 0 | 2 | 3 | 1 | 6 | 1 | 1 | 1 | 4 |
| miR444e |  |  |  |  |  |  |  |  |  |  |  |  |  | GCTAGAGGCACCAACTGCATA | 0 | 0 | 0 | 0 | 0 | 0 | 0 | 0 | 0 | 0 | 0 | 0 |
| miR444f | TGCAGTTGTTGCCTCAAGCTT | 1 | 0 | 1 | 0 | 2 | 1 | 2 | 6 | 1 | 1 | 3 | 1 | AAGCATGAGGTTACAACTGCATT | 0 | 0 | 0 | 0 | 0 | 0 | 0 | 0 | 0 | 0 | 0 | 0 |
| miR445a,h | TAAATTAGTGTATAAACATCCGAT | 0 | 0 | 0 | 0 | 0 | 0 | 0 | 0 | 0 | 0 | 0 | 0 | TGAATGTTTGACACTAATTTGGA | 0 | 0 | 0 | 0 | 0 | 0 | 0 | 0 | 0 | 0 | 0 | 0 |
| miR445b,i |  |  |  |  |  |  |  |  |  |  |  |  |  | CGAATGTTTGACGCTAATTTAGA | 0 | 0 | 0 | 0 | 0 | 0 | 0 | 0 | 0 | 0 | 0 | 0 |
| miR445c,e,f,g |  |  |  |  |  |  |  |  |  |  |  |  |  | CGAATGTTTGACGCTAATTTGGA | 0 | 0 | 0 | 0 | 0 | 0 | 0 | 0 | 0 | 0 | 0 | 0 |
| miR445d |  |  |  |  |  |  |  |  |  |  |  |  |  | CAGATGTTATGACACTAATTAAAAG | 0 | 0 | 0 | 0 | 0 | 0 | 0 | 0 | 0 | 0 | 0 | 0 |
| miR446 | CATCAATATGAATATGGGAAATGG | 0 | 0 | 0 | 0 | 0 | 0 | 0 | 0 | 0 | 0 | 0 | 0 | ATTTCCCACATTTATATTGATGTT | 0 | 0 | 0 | 0 | 0 | 0 | 0 | 0 | 0 | 0 | 0 | 0 |
| miR528 | TGGAAGGGGCATGCAGAGGAG | 631 | 849 | 415 | 469 | 75 | 120 | 79 | 783 | 24 | 159 | 124 | 235 | CCTGTGCTTGCCTCTTCCATT | 0 | 0 | 0 | 0 | 0 | 0 | 13 | 4 | 0 | 1 | 36 | 5 |
| miR529a | CTGTACCCTCTCTCTTCTTC | 0 | 0 | 0 | 0 | 0 | 0 | 0 | 0 | 0 | 0 | 0 | 0 | AGAAGAGAGAGAGTACAGCCT | 3 | 2 | 6 | 2 | 4 | 10 | 23 | 4 | 1 | 8 | 23 | 1 |
| miR529b | AGAAGAGAGAGAGTACAGCTT | 3 | 2 | 5 | 1 | 1 | 1 | 6 | 2 | 0 | 3 | 6 | 1 | TGTACGCTCCCTCTTCTTCT | 0 | 0 | 0 | 0 | 0 | 0 | 0 | 0 | 0 | 0 | 0 | 0 |
| miR530-3p | AGGTGCAGAGGCAGATGCAAC | 0 | 0 | 1 | 1 | 3 | 87 | 3 | 8 | 1 | 0 | 8 | 1 | - |  |  |  |  |  |  |  |  |  |  |  |  |
| miR530-5p | TGCATTTGCACCTGCACCTA | 0 | 0 | 0 | 0 | 0 | 0 | 0 | 0 | 0 | 0 | 0 | 0 | - |  |  |  |  |  |  |  |  |  |  |  |  |
| miR531 | CTCGCCGGGGCTGCGTGCCGCCAT | 0 | 0 | 0 | 0 | 0 | 0 | 0 | 0 | 0 | 0 | 0 | 0 | AGCTGGCGCGCATCCCCGTCGAGC | 0 | 0 | 0 | 0 | 0 | 0 | 0 | 0 | 0 | 0 | 0 | 0 |
| miR531b | CTCGCCGGGGCTGCGTGCCG | 0 | 0 | 0 | 0 | 0 | 0 | 0 | 0 | 0 | 0 | 0 | 0 | GGTGCGCATCCCCGTCGAGCG | 0 | 0 | 0 | 0 | 0 | 0 | 0 | 0 | 0 | 0 | 0 | 0 |
| miR535 | TGACAACGAGAGAGAGCACGC | 1152 | 713 | 2669 | 630 | 93 | 149 | 138 | 306 | 77 | 99 | 138 | 241 | GTGCTTTCTCCCGTTGTCACT | 4 | 1 | 3 | 1 | 10 | 10 | 44 | 14 | 9 | 5 | 66 | 14 |
| miR806a | ATGTGCTAAAAAGTCAACGGTG | 0 | 0 | 0 | 0 | 0 | 0 | 0 | 0 | 0 | 0 | 0 | 0 | CCGTTGACTTTTTAAATACGTGT | 0 | 0 | 0 | 0 | 0 | 0 | 0 | 0 | 0 | 0 | 0 | 0 |
| miR806b |  |  |  |  |  |  |  |  |  |  |  |  |  | CCGTTGACTTTTTAAGTACGTGT | 0 | 0 | 0 | 0 | 0 | 0 | 0 | 0 | 0 | 0 | 0 | 0 |
| miR806c |  |  |  |  |  |  |  |  |  |  |  |  |  | CCGTTGACTTTTTAGTACGTAT | 0 | 0 | 0 | 0 | 0 | 0 | 0 | 0 | 0 | 0 | 0 | 0 |
| miR806d |  |  |  |  |  |  |  |  |  |  |  |  |  | CCGTTGAGTTTTTAATACGTGT | 0 | 0 | 0 | 0 | 0 | 0 | 0 | 0 | 0 | 0 | 0 | 0 |
| miR806e |  |  |  |  |  |  |  |  |  |  |  |  |  | CCGTTGACTTTTTAGCATATGT | 0 | 0 | 0 | 0 | 0 | 0 | 0 | 0 | 0 | 0 | 0 | 0 |
| miR806f |  |  |  |  |  |  |  |  |  |  |  |  |  | CCGTTGACTTTTTATTACATGT | 0 | 0 | 0 | 0 | 0 | 0 | 0 | 0 | 0 | 0 | 0 | 0 |
| miR806g |  |  |  |  |  |  |  |  |  |  |  |  |  | CCGCTGACTTTTTAGTACGTGT | 0 | 0 | 0 | 0 | 0 | 0 | 0 | 0 | 0 | 0 | 0 | 0 |
| miR806h |  |  |  |  |  |  |  |  |  |  |  |  |  | CCGTTGACTTTTTATCATATGT | 0 | 0 | 0 | 0 | 0 | 0 | 0 | 0 | 0 | 0 | 0 | 0 |
| miR807a | CGTCATCTCACAGGTGAATCC | 0 | 0 | 0 | 0 | 0 | 0 | 0 | 0 | 0 | 0 | 0 | 0 | ATTCACCCGTGAGATGACTTG | 0 | 0 | 0 | 0 | 0 | 0 | 0 | 0 | 0 | 0 | 0 | 0 |
| miR807b |  |  |  |  |  |  |  |  |  |  |  |  |  | ATTCACCTGTGATATGGCGTG | 0 | 0 | 0 | 0 | 0 | 0 | 0 | 0 | 0 | 0 | 0 | 0 |
| miR807c |  |  |  |  |  |  |  |  |  |  |  |  |  | ATTCACCCGTGATATGACGTG | 0 | 0 | 0 | 0 | 0 | 0 | 0 | 0 | 0 | 0 | 0 | 0 |
| miR808 | ATGAATGTGGGAAATGTAAGAA | 0 | 0 | 0 | 0 | 0 | 0 | 0 | 0 | 0 | 0 | 0 | 0 | CTAGCATTTTCCACATTCATAT | 0 | 0 | 0 | 0 | 0 | 0 | 0 | 0 | 0 | 0 | 0 | 0 |
| miR809a | TGAATGTGAGAAATGTTAGAAT | 0 | 0 | 0 | 0 | 0 | 0 | 0 | 0 | 0 | 0 | 0 | 0 | TATAGCATTTTTCAAATTCATA | 0 | 0 | 0 | 0 | 0 | 0 | 0 | 0 | 0 | 0 | 0 | 0 |
| miR809b.d |  |  |  |  |  |  |  |  |  |  |  |  |  | TCTAGCATTTCCCACATTCATA | 0 | 0 | 0 | 0 | 0 | 0 | 0 | 0 | 0 | 0 | 0 | 0 |
| miR809c |  |  |  |  |  |  |  |  |  |  |  |  |  | TCTAGCATATCCTATATTCATA | 0 | 0 | 0 | 0 | 0 | 0 | 0 | 0 | 0 | 0 | 0 | 0 |
| miR809e |  |  |  |  |  |  |  |  |  |  |  |  |  | TCTAACATTTCCTACATTTATA | 0 | 0 | 0 | 0 | 0 | 0 | 0 | 0 | 0 | 0 | 0 | 0 |
| miR809f |  |  |  |  |  |  |  |  |  |  |  |  |  | TCTAACATTTCCCACATTCATA | 0 | 0 | 0 | 0 | 0 | 0 | 0 | 0 | 0 | 0 | 0 | 0 |
| miR810a | TCATAAGCCCACCACATGTGG | 0 | 0 | 0 | 0 | 1 | 1 | 2 | 4 | 0 | 4 | 2 | 1 | ACATGTGGTGAGCTTACGATG | 0 | 0 | 0 | 0 | 0 | 0 | 1 | 0 | 0 | 1 | 0 | 0 |
| miR810b.1 | TGAACACCGATATGCGTCATC | 4 | 1 | 1 | 1 | 7 | 7 | 6 | 26 | 6 | 11 | 8 | 35 | ACGACGTATATCGGTGTTCGC | 5 | 4 | 4 | 2 | 22 | 9 | 24 | 33 | 10 | 44 | 16 | 26 |
| miR810b.2 | AAGTGATTTAATTATGCCGTT | 0 | 0 | 0 | 0 | 1 | 0 | 1 | 3 | 0 | 3 | 1 | 0 | CGGCATAATTAGATCACTTGAT | 14 | 35 | 3 | 5 | 1 | 1 | 1 | 1 | 0 | 2 | 1 | 1 |
| miR811a | ACCGTTAGATCGAGAAATGGACGT | 0 | 0 | 0 | 0 | 0 | 0 | 0 | 0 | 0 | 0 | 0 | 0 | GTCCATTTCTCGATCCAACGTTAG | 0 | 0 | 0 | 0 | 0 | 0 | 0 | 0 | 0 | 0 | 0 | 0 |
| miR811b | ACCGTTAGATCGAGAAATGGACGT | 0 | 0 | 0 | 0 | 0 | 0 | 0 | 0 | 0 | 0 | 0 | 0 | GTTCGTTTCTGGATCCAACGGTAG | 0 | 0 | 0 | 0 | 0 | 0 | 0 | 0 | 0 | 0 | 0 | 0 |
| miR811c |  |  |  |  |  |  |  |  |  |  |  |  |  | GTCTATTTCTCGATCCAACGGT | 0 | 0 | 0 | 0 | 0 | 0 | 0 | 0 | 0 | 0 | 0 | 0 |
| miR812a | GACGGACGGTTAAACGTTGGAC | 0 | 0 | 0 | 0 | 0 | 0 | 0 | 0 | 0 | 0 | 0 | 0 | CCAAAGTTTGATCGTTCGTCTT | 0 | 0 | 0 | 0 | 0 | 0 | 0 | 0 | 0 | 0 | 0 | 0 |
| miR812b |  |  |  |  |  |  |  |  |  |  |  |  |  | CTACGTTTGACCGTTCGTCTT | 0 | 0 | 0 | 0 | 0 | 0 | 0 | 0 | 0 | 0 | 0 | 0 |
| miR812c |  |  |  |  |  |  |  |  |  |  |  |  |  | CTAATGTTTGACCGTCCATCTT | 0 | 0 | 0 | 0 | 0 | 0 | 0 | 0 | 0 | 0 | 0 | 0 |
| miR812d |  |  |  |  |  |  |  |  |  |  |  |  |  | CCAACATTTGATCATCTGCCTT | 0 | 0 | 0 | 0 | 0 | 0 | 0 | 0 | 0 | 0 | 0 | 0 |
| miR812e |  |  |  |  |  |  |  |  |  |  |  |  |  | CCAACTTTGACCGTCCGTCTT | 0 | 0 | 0 | 0 | 0 | 0 | 0 | 0 | 0 | 0 | 0 | 0 |
| miR812f |  |  |  |  |  |  |  |  |  |  |  |  |  | GTGTCTAACTTTGATCGTCCGTTT | 0 | 0 | 0 | 0 | 0 | 0 | 0 | 0 | 0 | 0 | 0 | 0 |
| miR812g |  |  |  |  |  |  |  |  |  |  |  |  |  | CCCAACTTTAATCATCTGTTTTAT | 0 | 0 | 0 | 0 | 0 | 0 | 0 | 0 | 0 | 0 | 0 | 0 |
| miR812h |  |  |  |  |  |  |  |  |  |  |  |  |  | CTCAACTTTAATCGTCCGTCTTAT | 0 | 0 | 0 | 0 | 0 | 0 | 0 | 0 | 0 | 0 | 0 | 0 |
| miR812i |  |  |  |  |  |  |  |  |  |  |  |  |  | TTCAACTTAGATCATCTGTCTTAT | 0 | 0 | 0 | 0 | 0 | 0 | 0 | 0 | 0 | 0 | 0 | 0 |
| miR812j |  |  |  |  |  |  |  |  |  |  |  |  |  | TCCAACTTTAATTGTTCGTCTTAT | 0 | 1 | 1 | 0 | 0 | 0 | 0 | 0 | 0 | 0 | 0 | 0 |
| miR813 | GGGTTATGGAATGGGTTTTACC | 0 | 0 | 0 | 0 | 0 | 0 | 0 | 0 | 0 | 0 | 0 | 0 | CAAAACCAATTCCATAACCCCT | 0 | 0 | 0 | 0 | 0 | 0 | 0 | 0 | 0 | 0 | 0 | 0 |
| miR814a | CACTTCATAGTACAACGAATCT | 0 | 0 | 0 | 0 | 0 | 0 | 0 | 0 | 0 | 0 | 0 | 0 | ATTCGTTGTACTATGAAGTGTC | 0 | 0 | 0 | 0 | 0 | 0 | 0 | 0 | 0 | 0 | 0 | 0 |
| miR814b |  |  |  |  |  |  |  |  |  |  |  |  |  | ATTCGTTGTACTATAAAGTGTC | 0 | 0 | 0 | 0 | 0 | 0 | 0 | 0 | 0 | 0 | 0 | 0 |
| miR814c |  |  |  |  |  |  |  |  |  |  |  |  |  | ATTCGTTATACTATAAAGTGTC | 0 | 0 | 0 | 0 | 0 | 0 | 0 | 0 | 0 | 0 | 0 | 0 |
| miR815a | AAGGGGATTGAGGAGATTGGG | 0 | 0 | 0 | 0 | 0 | 0 | 0 | 0 | 0 | 0 | 0 | 0 | TAGACTCTTAATTTCTTTTT | 0 | 0 | 0 | 0 | 0 | 0 | 0 | 0 | 0 | 0 | 0 | 0 |
| miR815b |  |  |  |  |  |  |  |  |  |  |  |  |  | CAATCTTCTCTATTTCCTTCT | 0 | 0 | 0 | 0 | 0 | 0 | 0 | 0 | 0 | 0 | 0 | 0 |
| miR815c |  |  |  |  |  |  |  |  |  |  |  |  |  | - | 0 | 0 | 0 | 0 | 0 | 0 | 0 | 0 | 0 | 0 | 0 | 0 |
| miR816 | GTGACATATTTTACTACAAC | 0 | 0 | 0 | 0 | 0 | 0 | 0 | 0 | 0 | 0 | 0 | 0 | AGTATTAGGATGTGTCACAT | 0 | 0 | 0 | 0 | 0 | 0 | 0 | 0 | 0 | 0 | 0 | 0 |
| miR817 | TCCAACTTGAGGCCCGATTGA | 0 | 0 | 0 | 0 | 0 | 0 | 0 | 0 | 0 | 0 | 0 | 0 | AATCAGGCCTCAAGTTGGTGT | 0 | 0 | 0 | 0 | 0 | 0 | 0 | 0 | 0 | 0 | 0 | 0 |
| miR818a | AATCCCTTATATTATGGGACGG | 0 | 0 | 0 | 0 | 0 | 0 | 0 | 0 | 0 | 0 | 1 | 0 | ATCCCATAGTATAAGGGATTTG | 0 | 0 | 0 | 0 | 0 | 0 | 0 | 0 | 0 | 0 | 0 | 0 |
| miR818b |  |  |  |  |  |  |  |  |  |  |  |  |  | GTCCCATAATATAAGAGATTTT | 0 | 0 | 0 | 0 | 0 | 0 | 0 | 0 | 0 | 0 | 0 | 0 |
| miR818c |  |  |  |  |  |  |  |  |  |  |  |  |  | GTCCTATAATATAAGGGATTTT | 0 | 0 | 0 | 0 | 0 | 0 | 0 | 0 | 0 | 0 | 0 | 0 |
| miR818d |  |  |  |  |  |  |  |  |  |  |  |  |  | ATCCCATAATATAAGGGATTTT | 0 | 0 | 0 | 0 | 0 | 0 | 0 | 0 | 0 | 0 | 0 | 0 |
| miR818e |  |  |  |  |  |  |  |  |  |  |  |  |  | GTCCTATAATATAAGGGATTTT | 0 | 0 | 0 | 0 | 0 | 0 | 0 | 0 | 0 | 0 | 0 | 0 |
| miR819a,b,e,g,k | TCAGGTTATAAGACTTTCTAGC | 0 | 0 | 0 | 0 | 0 | 0 | 0 | 0 | 0 | 0 | 0 | 0 | TAGAAAGTCTTATAATATGAAA | 0 | 0 | 0 | 0 | 0 | 1 | 0 | 0 | 0 | 1 | 0 | 0 |
| miR819c |  |  |  |  |  |  |  |  |  |  |  |  |  | TAGAAAGCCTTATAATATGAAA | 0 | 0 | 0 | 0 | 0 | 0 | 0 | 0 | 0 | 0 | 0 | 0 |
| miR819d |  |  |  |  |  |  |  |  |  |  |  |  |  | TAGAAAGTCTTATAACCTGAAA | 0 | 0 | 0 | 0 | 0 | 0 | 0 | 0 | 0 | 0 | 0 | 0 |
| miR819f |  |  |  |  |  |  |  |  |  |  |  |  |  | TAGAAAATTTTATAATATGAAA | 0 | 0 | 0 | 0 | 0 | 0 | 0 | 0 | 0 | 0 | 0 | 0 |
| miR819h |  |  |  |  |  |  |  |  |  |  |  |  |  | GAGAAAGTCTTATAACCTGAAA | 0 | 0 | 0 | 0 | 0 | 0 | 0 | 0 | 0 | 0 | 0 | 0 |
| miR819i |  |  |  |  |  |  |  |  |  |  |  |  |  | TAGAAAATCTTATAATATGAAA | 0 | 0 | 0 | 0 | 0 | 0 | 0 | 0 | 0 | 0 | 0 | 0 |
| miR819j |  |  |  |  |  |  |  |  |  |  |  |  |  | TAGAAAGTTTTATAATATGAAA | 0 | 0 | 0 | 0 | 0 | 0 | 0 | 0 | 0 | 0 | 0 | 0 |
| miR820a | TCGGCCTCGTGGATGGACCAG | 4 | 3 | 8 | 2 | 6 | 7 | 51 | 21 | 4 | 9 | 67 | 13 | AGGTCCGTCCAGGAGGACG | 0 | 0 | 0 | 0 | 0 | 0 | 0 | 0 | 0 | 0 | 0 | 0 |
| miR820b |  |  |  |  |  |  |  |  |  |  |  |  |  | AGGTCCGTCCACGAGGACGAC | 0 | 0 | 0 | 0 | 0 | 0 | 0 | 0 | 0 | 0 | 0 | 0 |
| miR820c |  |  |  |  |  |  |  |  |  |  |  |  |  | AGGTCCGTCCACGAGGACG | 0 | 0 | 0 | 0 | 0 | 0 | 0 | 0 | 0 | 0 | 0 | 0 |
| miR821a,b,c | AAGTCATCAACAAAAAAGTTGAAT | 0 | 0 | 0 | 0 | 1 | 0 | 0 | 0 | 0 | 0 | 0 | 0 | TCAACTTTGTTGTTGATGACTTTT | 0 | 0 | 0 | 0 | 0 | 0 | 0 | 0 | 0 | 0 | 0 | 0 |
| miR827 | TAAGATGACCATCAGCGAAAA | 0 | 0 | 0 | 0 | 0 | 0 | 0 | 0 | 0 | 0 | 0 | 0 | TTCGCTGACGGCCGATTTAAC | 0 | 0 | 0 | 0 | 0 | 0 | 0 | 0 | 0 | 0 | 0 | 0 |
