# Supplementary material for: Viral Infection Induces Expression of Novel Phased MicroRNAs from Conserved Cellular MicroRNA Precursors
Source: PLoS Pathog. 2011 Aug 25;7(8):e1002176. doi: 10.1371/journal.ppat.1002176 (PMC3161970; doi:10.1371/journal.ppat.1002176)
Supplement: Table S3 — Newly-identified miRNAs from the known miRNA precursors and their target genes. The footnotes of the table are as follows: a Reads was average values of three-repeat reads of each libraries, which normallized to 1 million with the total sequencing reads. b The precursor of miR394 is longer than the one in miRBase database, and miR394.2 have higher reads than miR394 only in RSV-infected rice library. c From the same position,other varient sequences with different length was produced. d The newly-identified miRNAs only have high reads in RSV-infected rice library, and form the phased-miRNA with the reported miRNA before . e The newly-identified miRNA are in tandem configuration with the reported miRNAs. f The sequences have much higher reads than the reported ones. g The miRNA sequences, reads number of which were outstanding shown in red and blue, were greatly accumulated and reduced only during RSV infection, respectively. (DOC) [file ppat.1002176.s009.doc]

| **miRNA** | **Sequence (5’-3’)** | **Length** | **NO. of**  **loci** | **No. of reads (/million)^a^** | | | | **Location** | **Predicted**  **target(s)** |
| --- | --- | --- | --- | --- | --- | --- | --- | --- | --- |
|  |  |  |  | **RDV** | **Mock**  **(RDV)** | **RSV^g^** | **Mock**  **(RSV)** |  |  |
| miR156d-21^c^ | UUGACAGAAGAGAGUGAGCAC | 21 | 4 | 5890 | 7041 | 1933 | 4202 | Chr2, intergenic | Os04g46580 |
| miR156f-21 |  |  |  |  |  |  |  | Chr8, intergenic | Os07g32170 |
| miR156h-21 |  |  |  |  |  |  |  | Chr6, intergenic |  |
| miR156j-21 |  |  |  |  |  |  |  | Chr2, intergenic |  |
| miR159a.2 ^d^ | UUGCAUGCCCCAGGAGCUGC | 20 | 1 | 30 | 24 | 76 | 23 | Chr1, intergenic | Os03g02240 |
| miR159a.2*^d^, | AGCUGCUGGGUCAUGGAUCCC | 21 | 1 | 2 | 4 | 219 | 2 | Chr1, intergenic | - |
| miR159a.3^d^, | AUCAACCCUACAUGGACCCUC | 21 | 1 | 4 | 2 | 361 | 2 | Chr1, intergenic | Os12g03530 |
| miR159a.3*^d^ | GGGGUGUUGCUGUGGGUCGAUU | 22 | 1 | 1 | 1 | 63 | 0 | Chr1, intergenic | Os08g39300 |
| miR159b.2^d^ | UUUGCAUGACCAAGGAGCCGA | 21 | 1 | 1 | 0 | 118 | 0 | Chr1, intergenic | - |
| miR159c.2-21^d^ | UUUGCAUGACCCGGGAGAUGA | 21 | 2 | 5 | 3 | 6 | 2 | Chr1, intergenic | Os07g32060 |
| miR159f.2-21 |  |  |  |  |  |  |  | Chr1, intergenic | Os11g09620 |
| miR159c.2-20 ^d^ | UUUGCAUGACCCGGGAGAUG | 20 | 2 | 0 | 0 | 19 | 0 | Chr1, intergenic | Os09g36090 |
| miR159f.2-20 |  |  |  |  |  |  |  | Chr1, intergenic |  |
| miR167d-22^c^ | UGAAGCUGCCAGCAUGAUCUGA | 22 | 6 | 4260 | 6070 | 1910 | 6777 | Chr7, intergenic | Os02g06910 |
| miR167f-22 |  |  |  |  |  |  |  | Chr10, intergenic | Os06g46410 |
| miR167g-22 |  |  |  |  |  |  |  | Chr3, intergenic | Os12g41950 |
| miR167h-22 |  |  |  |  |  |  |  | Chr12, intergenic |  |
| miR167i-22 |  |  |  |  |  |  |  | Chr6, intergenic |  |
| miR167j-22 |  |  |  |  |  |  |  | Chr1, intergenic |  |
| miR171i.2 ^e^ | UUGAGCCGCGUCAAUAUCUCU | 21 | 1 | 32 | 44 | 85 | 102 | Chr3, intergenic | Os02g44360 |
|  |  |  |  |  |  |  |  |  | Os04g46860 |
|  |  |  |  |  |  |  |  |  | Os02g44370 |
|  |  |  |  |  |  |  |  |  | Os06g01620 |
|  |  |  |  |  |  |  |  |  | Os10g40390 |
| miR394.2^b,d^ | AAGGGGCGCUUACUGAGAGU | 20 | 1 | 0 | 0 | 18 | 0 | Chr2, intergenic | Os02g47000 |
| miR397a.2 ^e^ | UUGAGUGCAGCGUUGAUGAAC | 21 | 2 | 115 | 171 | 155 | 400 | Chr6, intergenic | Os05g38410 |
| miR397b.2 ^e^ |  |  |  |  |  |  |  | Chr2, intergenic | Os05g38420 |
| miR1318-22^f^ | AUCAGGAGAGAUGACACCGACA | 22 | 2 | 219 | 247 | 121 | 130 | Chr7, intergenic | - |
| miR1432-22^f^ |  |  |  |  |  |  |  | Chr1, intergenic |  |
| miR1429-5p-24^c^ | GUAAUAUACUAAUCCGUGCAUCCA | 24 | 1 | 36 | 18 | 17 | 35 | Chr8, intergenic | - |
| miR1868.2 ^e^ | GCGUGCUCACGGAAAACGAGGGAG | 24 | 1 | 40 | 43 | 15 | 57 | Chr4, intergenic | Os04g57670 |
|  |  |  |  |  |  |  |  |  | Os03g27120 |
|  |  |  |  |  |  |  |  |  | Os04g57670 |
