# Supplementary material for: Viral Infection Induces Expression of Novel Phased MicroRNAs from Conserved Cellular MicroRNA Precursors
Source: PLoS Pathog. 2011 Aug 25;7(8):e1002176. doi: 10.1371/journal.ppat.1002176 (PMC3161970; doi:10.1371/journal.ppat.1002176)
Supplement: Table S4 — Primers and probes used in this paper. * indicates the corresponding miRNA star sequences. (DOC) [file ppat.1002176.s010.doc]

| **Primers used for quantitative real-time RT-PCR** | |
| --- | --- |
| **Primer** | **Sequence (5’-3’)** |
| OsDCL1a-F | ATCCAATGGTGACGCCAGAG |
| OsDCL1a-R | CATTCCTCGCTGCCAACTTC |
| OsDCL1b-F | AACCTTGACGGCCAGATTAC |
| OsDCL1b-R | GGATGCTTGCCTAATGTCTC |
| OsDCL1c-F | AAGCAAGAGCCAGAGGATGA |
| OsDCL1c-R | GTCACAGGTTGCTCGTCTATTG |
| OsDCL2-F | GCGGCTATTGGAACCTCTTG |
| OsDCL2-R | CGAGCAATGACCGATGAGAC |
| OsDCL3a-F | AGCATTGTGGCAGCACTAATCC |
| OsDCL3a-R | ATGCAATGCGTGGTCCTCCT |
| OsDCL3b-F | ATTGCCACCTTACCGTGAGTTG |
| OsDCL3b-R | CTTGTGCTACCAGCAGCTCATC |
| SHO1-F | GCTTGCTTATGCGGCGGTTG |
| SHO1-R | GCACTGCGCCAATACAAGATTC |
| OsRDR1-F | ATTCCGCCTCGTATGGTGAC |
| OsRDR1-R | TGCCTTGAGGTCTTCCTTATCC |
| OsRDR2-F | GACTCGCTTGGTGCCATCTC |
| OsRDR2-R | CGTCTTGGCGAAGTCAACTG |
| OsRDR4-F | CGCAGTCCTTCGTACCATTC |
| OsRDR4-R | TAGCGGTGTTGCCAGAGTTG |
| SHL2-F | TAAGAACAGCAGCAAGAAGCAAG |
| SHL2-R | ATAGACAGCCCTCAACTGAAACG |
| OsAGO1a-F | TTGGTCTCGCAATGCTGTCT |
| OsAGO1a-R | TCGTTGGCCTATCACTCACA |
| OsAGO1b-F | GTCCAGAAGCGGCATCACAC |
| OsAGO1b-R | TAATGAGCAGGACGGCTTGTTC |
| OsAGO1c-F | TAGCCATGCTGGCATTAAGG |
| OsAGO1c-R | GAAGGCAGCCAGATGAGCATAA |
| OsAGO1d-F | CGCCACCACACGAGATTATT |
| OsAGO1d-R | GCATGGCTGCACAAGAAGAA |
| OsAGO2-F | ACCAGCAGCAGACCAAGAAC |
| OsAGO2-R | GCTCGTCCAGAAGGCTGTAG |
| OsAGO3-F | AGGAACCAGCGGCAGACCAA |
| OsAGO3-R | GCGAAGACGAAGCAGAGGTTGT |
| OsAGO11-F | GATGCAATTCGGAAGGCTTGTG |
| OsAGO11-R | CCTCGCGTGATGATCTTCAGGA |
| OsAGO13-F | CATGGTAGGCGAGTTATTGC |
| OsAGO13-R | CGCCAATCGTATCATTGC |
| OsAGO16-F | TTCACATGGTTCGCCAGGTC |
| OsAGO16-R | TTCCATCGTCCAGTGGCTTA |
| OsAGO18-F | TGTTCGTCCAGGCACAGTAG |
| OsAGO18-R | GCGGTGAAGTTGTTGTCGTC |
| Os02g49240-F | TTGCCGGATCAAGCACCACC |
| Os02g49240-R | ATCCAAGCTCGACGCGAGAG |
| Os03g43930-F | GGCTGATCCTGGTGTTGATG |
| Os03g43930-R | ATCATGGCAGAGGCTGTGTC |
| Os03g52630-F | ACAAGCTTCCAGGAGCTCAG |
| Os03g52630-R | ATACTGAAGTGGCTGAGGCC |
| Os11g38140-F | GGCGACCATAGTTGGACAAG |
| Os11g38140-R | AGACTTGCAACATATCGCCG |
| Os03g60430-F | GGCGTCACCTTCTACAGGAG |
| Os03g60430-R | CAGATCGTCCTCGTAGTCGC |
| Os04g57610-F | GGCTCCATCCATCCTTACTG |
| Os04g57610-R | ATCTGTTGCTGGCTTGCTTG |
| Os12g41680-F | TTCTCCTCCAAGGAAGACTG |
| Os12g41680-R | CTGTTGCTGAAGCTGGAGAA |
| Os03g02240-F | CTACGGCAACGACAGAATGG |
| Os03g02240-R | ACGACGACGTTCTGGTTCTG |
| Os12g03530-F | GCTTGACCATCCTGCTGATT |
| Os12g03530-R | TCAGCCAGTGAAGCAAGAAG |
| Os02g47000-F | GGAATCTTCTCCAGGTTCTC |
| Os02g47000-R | CAATGCCATGCGGACTATCT |
| **Probes used for small RNA northern blots** | |
| **DNA oligo probe** | **Sequence (5’-3’)** |
| miR156 | GTGCTCACTCTCTTCTGTCA |
| miR159.1 | CAGAGCTCCCTTCAATCCAAA |
| miR159.2 | CGGCTCCTTGGTCATGCAAA |
| miR159.2* | GGATCCATGACCCAGCAGCT |
| miR159.3 | GAGGGTCCATGTAGGGTTGAT |
| miR160 | TGGCATACAGGGAGCCAGGCA |
| miR160* | TATGCTTGGCTCCGTGCACGC |
| miR164 | TGCACGTGCCCTGCTTCTCCA |
| miR166 | GGGGAATGAAGCCTGGTCCGA |
| miR167 | CAGATCATGCTGGCAGCTTCA |
| miR168 | GTCCCGATCTGCACCAAGCGA |
| miR171 | GATATTGGCACGGCTCAATCA |
| miR171* | TCTGAGTGAGCCGGGCCAACA |
| miR172 | ATGCAGCATCATCAAGATTCT |
| miR394.2 | ACTCTCAGTAAGCGCCCCTT |
| miR1425 | AGCAGCAAGGATTGAATCCTA |
| miR1425* | ATTAAGATCCAGTTCTTGCTG |
| AK120922-P5 | TAGCATTTCCTCCGATGTCTC |
| AK120922-P10 | TGTTACATTGCTTCATGCCTT |
| U6 | TTCCCGATCGGTCACCCATCCCAAAATTGCT  CCAAGCCAAGCACGCTTAACCTAAGAGTTCT |
| **Primers used for 5’RACE** | |
| **Primer** | **Sequence (5’-3’)** |
| Os04g47870-outer | GCCAACTTTCCAAACCCTCAC |
| Os04g47870-inner | TCCTTCCATCATAGGCAGGTAGAC |
| Os11g15060-outer | TCCCGCAGTGTGAGTGGCTGAAATGC |
| Os11g15060-inner | AGTCCAGCTTCTCCTTGGCAACAC |
| **Primers used for PCR of mRNA probes** | |
| **Primer** | **Sequence (5’-3’)** |
| Os11g15060-F | GAGGCGATCACCACAATATGTGAG |
| Os11g15060-R | TGGTGGTGTAGTCGCTCCTATGTG |
