# Supplementary material for: Viral Infection Induces Expression of Novel Phased MicroRNAs from Conserved Cellular MicroRNA Precursors
Source: PLoS Pathog. 2011 Aug 25;7(8):e1002176. doi: 10.1371/journal.ppat.1002176 (PMC3161970; doi:10.1371/journal.ppat.1002176)
Supplement: Table S5 — Expressional analysis of RNA silencing pathway genes by microarray. The footnote of the table is as follows: a Fold change = (normalized signal intensity of virus-infected rice samples)/ (normalized signal intensity of mock-inoculated rice samples). (DOC) [file ppat.1002176.s011.doc]

| **Probe Set ID** | **Genes** | **Fold change^a^ (RDV infection)** | | | |  | **Fold change^a^ (RSV infection)** | | | |
| --- | --- | --- | --- | --- | --- | --- | --- | --- | --- | --- |
|  |  | **Ave.** | **Slide1** | **Slide2** | **Slide3** |  | **Ave.** | **Slide1** | **Slide2** | **Slide3** |
| Os.13488.1.S1_at | LOC_Os03g02970,OsDCL1a | 1.08 | 0.94 | 0.84 | 1.67 |  | 1.35 | 1.40 | 1.83 | 1.00 |
| OsAffx.24947.1.S1_at | LOC_Os03g02970,OsDCL1a | 1.00 | 0.86 | 0.77 | 1.51 |  | 1.02 | 1.98 | 0.64 | 0.77 |
| Os.28186.1.S1_s_at | LOC_Os03g38740,OsDCL2a | 1.45 | 1.22 | 1.51 | 1.70 |  | 1.87 | 1.64 | 1.94 | 2.18 |
| Os.28186.2.S1_s_at | LOC_Os03g38740,OsDCL2a | 1.43 | 1.33 | 1.35 | 1.65 |  | 2.45 | 2.96 | 1.92 | 2.67 |
| Os.50406.1.S1_x_at | LOC_Os03g38740,OsDCL2a | 1.38 | 1.21 | 1.37 | 1.64 |  | 2.17 | 2.42 | 1.73 | 2.52 |
| Os.28186.1.S1_s_at | LOC_Os09g14610,OsDCL2b | 1.45 | 1.22 | 1.51 | 1.70 |  | 1.87 | 1.64 | 1.94 | 2.18 |
| Os.28186.2.S1_s_at | LOC_Os09g14610,OsDCL2b | 1.43 | 1.33 | 1.35 | 1.65 |  | 2.45 | 2.96 | 1.92 | 2.67 |
| Os.50406.1.S1_x_at | LOC_Os09g14610,OsDCL2b | 1.38 | 1.21 | 1.37 | 1.64 |  | 2.17 | 2.42 | 1.73 | 2.52 |
| Os.49417.1.S1_at | LOC_Os01g68120,OsDCL3a | 0.85 | 0.77 | 0.84 | 0.96 |  | 0.78 | 0.89 | 0.75 | 0.70 |
| OsAffx.8007.1.S1_at | LOC_Os10g34430,OsDCL3b | 0.87 | 0.69 | 0.94 | 1.02 |  | 1.11 | 1.48 | 0.86 | 1.07 |
| Os.27988.1.A1_at | LOC_Os04g43050,SHO1 | 1.20 | 1.12 | 1.08 | 1.47 |  | 1.20 | 1.31 | 1.37 | 0.94 |
| OsAffx.26439.1.S1_x_at | LOC_Os04g43050,SHO1 | 1.28 | 0.98 | 1.00 | 2.56 |  | 0.82 | 1.30 | 0.55 | 0.62 |
| OsAffx.26948.1.S1_at | LOC_Os05g18850,OsDCL1c | 0.87 | 0.98 | 0.80 | 0.84 |  | 0.64 | 0.60 | 0.60 | 0.72 |
| Os.55064.1.S1_at | LOC_Os06g25250,OsDCL1b | 1.00 | 1.01 | 1.00 | 0.99 |  | 1.02 | 1.06 | 1.24 | 0.80 |
| Os.8293.1.S1_at | LOC_Os02g45070,OsAGO1a | 1.17 | 1.24 | 1.09 | 1.18 |  | 1.76 | 1.87 | 1.55 | 1.87 |
| Os.10753.1.S1_a_at | LOC_Os04g47870,OsAGO1b | 1.48 | 1.29 | 1.17 | 2.10 |  | 2.64 | 3.97 | 1.83 | 2.54 |
| Os.10753.1.S2_a_at | LOC_Os04g47870,OsAGO1b | 1.35 | 1.35 | 1.16 | 1.56 |  | 2.29 | 2.77 | 2.00 | 2.13 |
| Os.7419.1.S1_a_at | LOC_Os02g58490,OsAGO1c | 1.02 | 1.14 | 0.83 | 1.10 |  | 1.04 | 1.31 | 0.95 | 0.90 |
| Os.18590.1.S1_a_at | LOC_Os06g51310,OsAGO1d | 1.61 | 1.89 | 1.25 | 1.70 |  | 1.76 | 1.58 | 1.52 | 2.34 |
| Os.50333.1.S1_at | LOC_Os04g52540,OsAGO2 | 1.16 | 0.97 | 1.08 | 1.58 |  | 1.90 | 1.95 | 2.07 | 1.76 |
| Os.55120.1.S1_at | LOC_Os04g52550,OsAGO3 | 1.07 | 1.06 | 1.05 | 1.10 |  | 1.08 | 1.00 | 0.96 | 1.31 |
| Os.12351.1.S1_at | LOC_Os01g16870,OsAGO4a | 1.08 | 1.21 | 0.98 | 1.05 |  | 0.89 | 1.04 | 0.76 | 0.84 |
| Os.10217.1.S1_a_at | LOC_Os04g06770,OsAGO4b | 0.90 | 0.94 | 0.85 | 0.91 |  | 0.76 | 0.99 | 0.60 | 0.70 |
| Os.53826.1.S1_at | LOC_Os07g09020,OsAGO14 | 1.08 | 0.98 | 1.20 | 1.04 |  | 1.07 | 0.91 | 0.75 | 1.77 |
| Os.40026.1.S1_at | LOC_Os03g58600,OsMEL1 | 1.09 | 0.96 | 1.22 | 1.09 |  | 1.05 | 1.09 | 0.97 | 1.08 |
| Os.50897.1.S1_at | LOC_Os03g57560,OsAGO13 | 1.14 | 1.00 | 1.49 | 0.96 |  | 1.17 | 1.43 | 1.24 | 0.85 |
| OsAffx.13526.1.S1_x_at | LOC_Os03g57560,OsAGO13 | 1.06 | 0.88 | 1.21 | 1.10 |  | 1.14 | 1.58 | 1.04 | 0.90 |
| Os.19635.1.S1_at | LOC_Os07g16224,OsAGO16 | 1.06 | 1.04 | 1.03 | 1.13 |  | 0.91 | 1.03 | 0.86 | 0.81 |
| OsAffx.5347.1.S1_at | LOC_Os07g16224,OsAGO16 | 1.02 | 0.88 | 1.48 | 0.88 |  | 1.19 | 1.00 | 1.14 | 1.72 |
| OsAffx.25355.1.S1_at | LOC_Os03g33650,SHL4 | 0.79 | 0.76 | 0.73 | 0.90 |  | 0.62 | 0.54 | 0.75 | 0.61 |
| Os.9351.1.S1_at | LOC_Os06g39640,OsPNH1 | 0.96 | 1.01 | 1.02 | 0.83 |  | 0.44 | 0.58 | 0.49 | 0.26 |
| Os.38098.1.S1_at | LOC_Os06g39640,OsPNH1 | 0.96 | 1.01 | 0.83 | 1.09 |  | 0.94 | 1.03 | 0.78 | 1.02 |
| Os.54612.1.A1_at | LOC_Os02g07310,OsAGO17 | 1.05 | 0.96 | 0.99 | 1.19 |  | 0.99 | 1.08 | 0.99 | 0.93 |
| Os.56162.1.A1_x_at | LOC_Os03g47820,OsAGO12 | 0.94 | 0.93 | 0.93 | 0.97 |  | 0.76 | 0.86 | 0.74 | 0.66 |
| OsAffx.25614.1.S1_at | LOC_Os03g47820,OsAGO12 | 0.89 | 0.93 | 0.82 | 0.93 |  | 1.42 | 1.39 | 1.35 | 1.53 |
| OsAffx.3547.1.S1_at | LOC_Os03g47830,OsAGO11 | 1.09 | 1.35 | 1.12 | 0.89 |  | 1.09 | 1.34 | 0.93 | 1.08 |
| Os.7338.1.S1_at | LOC_Os07g28850,OsAGO18 | 1.60 | 1.17 | 1.25 | 2.89 |  | 5.59 | 3.48 | 6.64 | 8.61 |
| Os.7338.2.A1_at | LOC_Os07g28850,OsAGO18 | 1.11 | 1.09 | 1.09 | 1.16 |  | 0.77 | 0.68 | 0.92 | 0.73 |
| OsAffx.28651.1.S1_x_at | LOC_Os07g28850,OsAGO18 | 1.67 | 1.34 | 0.99 | 3.06 |  | 4.41 | 4.07 | 3.27 | 6.56 |
| Os.30747.1.S1_at | LOC_Os01g34350,SHL2 | 0.63 | 0.66 | 0.69 | 0.54 |  | 0.72 | 0.73 | 0.75 | 0.68 |
| Os.51949.1.S1_at | LOC_Os04g39160,OsRDR2 | 0.72 | 0.78 | 0.70 | 0.67 |  | 0.54 | 0.58 | 0.49 | 0.53 |
| Os.24653.1.A1_s_at | LOC_Os04g39160,OsRDR2 | 0.91 | 0.99 | 0.88 | 0.86 |  | 1.03 | 1.44 | 0.83 | 1.00 |
| Os.2202.3.S1_x_at | LOC_Os01g10140,OsRDR4 | 1.16 | 1.00 | 1.23 | 1.30 |  | 1.19 | 1.90 | 0.72 | 1.02 |
| Os.2202.2.S1_x_at | LOC_Os01g10140,OsRDR4 | 1.24 | 1.48 | 1.30 | 1.02 |  | 1.14 | 1.26 | 0.91 | 1.28 |
| Os.54038.1.S1_at | LOC_Os02g50330,OsRDR1 | 1.62 | 1.51 | 1.47 | 1.90 |  | 1.60 | 1.82 | 1.48 | 1.49 |
| OsAffx.12575.1.S1_at | LOC_Os02g50330,OsRDR1 | 2.98 | 2.58 | 2.16 | 4.62 |  | 2.25 | 2.91 | 4.66 | 1.37 |
| Os.32212.1.A1_at | LOC_Os01g10130,OsRDR3 | 1.40 | 0.97 | 1.29 | 2.25 |  | 1.77 | 2.56 | 1.45 | 1.36 |
| Os.32212.2.S1_x_at | LOC_Os01g10130,OsRDR3 | 1.42 | 1.22 | 1.74 | 1.32 |  | 1.33 | 1.79 | 1.04 | 1.21 |
